# Seedling development traits in *Brassicanapus* examined by gene expression analysis and association mapping

**DOI:** 10.1186/s12870-015-0496-3

**Published:** 2015-06-09

**Authors:** Niklas Körber, Anja Bus, Jinquan Li, Janet Higgins, Ian Bancroft, Erin Eileen Higgins, Isobel Alison Papworth Parkin, Bertha Salazar-Colqui, Rod John Snowdon, Benjamin Stich

**Affiliations:** Max Planck Institute for Plant Breeding Research, Carl-von-Linné-Weg 10, Köln, 50829 Germany; Institute of Crop Science and Resource Conservation, Plant Breeding and Biotechnology, University of Bonn, Katzenburgweg 5, Bonn, 53115 Germany; The Genome Analysis Centre, Norwich Research Park, Norwich, NR4 7UH UK; John Innes Centre, Norwich Research Park, NR4 7UH Norwich, UK; Department of Biology, Wentworth Way, University of York, Heslington, York, YO41 5DD UK; Agriculture and Agri-Food Canada, 107 Science Place, Saskatoon, SK S7N OX2 Canada; Department of Plant Breeding, Research Centre for BioSystems, Land Use and Nutrition, Justus Liebig University, Heinrich-Buff-Ring 26-32, Giessen, 35392 Germany

**Keywords:** *Brassica napus*, Seedling development, RT-qPCR, Candidate genes, Genome-wide association mapping, Digital gene expression analysis (DGE-seq), Weighted gene co-expression network analysis (WGCNA), Plant breeding, Ribulose 1, 5-bisphosphate carboxylase/oxygenase small subunit, Fructose-1, 6-bisphosphatase, Linkage disequilibrium (LD)

## Abstract

**Background:**

An optimal seedling development of *Brassica napus* plants leads to a higher yield stability even under suboptimal growing conditions and has therefore a high importance for plant breeders. The objectives of our study were to (i) examine the expression levels of candidate genes in seedling leaves of *B. napus* and correlate these with seedling development as well as (ii) detect genome regions associated with gene expression levels and seedling development traits in *B. napus* by genome-wide association mapping.

**Results:**

The expression levels of the 15 candidate genes examined in the 509 *B. napus* inbreds showed an averaged standard deviation of 5.6 across all inbreds and ranged from 3.2 to 8.8. The gene expression differences between the 509 *B. napus* inbreds were more than adequate for the correlation with phenotypic variation of seedling development. The average of the absolute value correlations of the correlation coefficients of 0.11 were observed with a range from 0.00 to 0.39. The candidate genes *GER1*, *AILP1*, *PECT*, and *FBP* were strongly correlated with the seedling development traits. In a genome-wide association study, we detected a total of 63 associations between single nucleotide polymorphisms (SNPs) and the seedling development traits and 31 SNP-gene associations for the candidate genes with a *P*-value < 0.0001. For the projected leaf area traits we identified five different association hot spots on the chromosomes A2, A7, C3, C6, and C7.

**Conclusion:**

A total of 99.4% of the adjacent SNPs on the A genome and 93.0% of the adjacent SNPs on the C genome had a distance smaller than the average range of linkage disequilibrium. Therefore, this genome-wide association study is expected to result on average in 14.7% of the possible power. Compared to previous studies in *B. napus*, the SNP marker density of our study is expected to provide a higher power to detect SNP-trait/-gene associations in the *B. napus* diversity set. The large number of associations detected for the examined 14 seedling development traits indicated that these are genetically complex inherited. The results of our analyses suggested that the studied genes ribulose 1,5-bisphosphate carboxylase/oxygenase small subunit (*RBC*) on the chromosomes A4 and C4 and fructose-1,6-bisphosphatase precursor (*FBP*) on the chromosomes A9 and C8 are *cis*-regulated.

**Electronic supplementary material:**

The online version of this article (doi:10.1186/s12870-015-0496-3) contains supplementary material, which is available to authorized users.

## Background

Well-developed seedlings lead to a higher yield stability even under suboptimal growing conditions like reduced nutrient input or drought stress [1]. Therefore, variation during early developmental stages of *Brassica napus* plants is important for selection decisions of plant breeders. Up to now, however, the genetics of seedling development of *B. napus* had been poorly understood.

In comparison to linkage mapping, association mapping studies could achieve a higher mapping resolution due to the fact that in a diversity set linkage disequilibrium (LD) decays faster than in segregating populations used for linkage mapping [2]. Furthermore, association mapping studies benefit from the broader array of genetic diversity represented compared to linkage mapping studies [3,4]. Hasan et al. [5] identified in an association mapping study in *B. napus* simple sequence repeat (SSR) markers which were physically linked to candidate genes for glucosinolate biosynthesis in *Arabidopsis thaliana*, to be associated with variation of the seed glucosinolate content in *B. napus*. For traits, for which less preinformation is available, a high number of markers would be necessary to detect phenotype-marker associations on a genome-wide level. The number of SSR markers available in the *B. napus* genome is expected to be too low for this purpose [6]. Furthermore, the genotyping of such a high number of markers is very expensive. To overcome this problem, Honsdorf et al. [7] tested the association between 684 genome-wide distributed amplified fragment-length polymorphism (AFLP) markers and 14 traits in a set of 84 canola quality winter rapeseed cultivars. They identified between one and 22 putative quantitative trait loci (QTL) which explained between 15 and 53% of the phenotypic variance for ten of the 14 traits. The results of LD analyses suggested, however, that more than 2,000 evenly distributed markers will be required for detecting marker-phenotype associations with a reasonable power in rapeseed [2]. However, it is difficult to obtain a higher number of markers with the AFLP technique in rapeseed [7]. Furthermore, due to the fact that the sequence information of AFLPs can not be easily inferred, their use in marker-assisted selection programs is difficult. Hence, single nucleotide polymorphisms (SNPs) would be the most suitable marker type to cover a complex genome like that of *B. napus* in the required density for genome-wide association studies (GWAS). Therefore, a custom SNP array was used in this study to genotype the entire diversity set.

Differential expression of genes during seedling development stage has the potential to be an important reason for phenotypic variation [8,9]. In our study, genes were selected based on a co-expression network analysis. The gene expression of these genes as well as candidate genes from the literature was examined in the entire diversity set and correlated with the phenotypic observations.

The objectives of our study were to (i) examine the expression levels of candidate genes in seedling leaves of *B.napus* and correlate these with seedling development as well as (ii) identify genome regions associated with different gene expression levels and seedling development traits in *B. napus*.

## Methods

### Plant material and assessment of seedling development traits

A set of 509 rapeseed inbred lines (doi:10.1007/s00122-012-1912-9), assembled to maximize genotypic variation, was used in this study [2,10]. In short, according to available information from genebanks, plant breeders, and our own observations, the accessions were assigned to eight different germplasm types, namely winter oilseed rape (OSR) (183), winter fodder (22), swede (73), semi-winter OSR (7), spring OSR (204), spring fodder (4), vegetable (10), and so far unspecified rapeseed genotypes (6).

The multiplication of the genotypes was done in a way such that maternal environmental effects were minimized. The genotypes were grown in six replicates, for 30 days in an *α*-lattice design with 24 blocks of 24 pots in a greenhouse experiment. As described in detail earlier [10], a large number of seedling development traits were assessed to cover a wide range of aspects as well as developmental stages during seedling growth which could be measured with high throughput methods (Table 1).

**Table 1:** **Seedling development traits assessed in the rapeseed diversity set, where** ***h*** ^**2**^ **is the repeatability and** ***R*** ^**2**^ **the proportion of the phenotypic variance explained by population structure**

**Traits**	**Abbreviation**	**Unit of measurement**	***h*** ^***2***^	***R*** ^***2***^ ** (MCLUST)**
Projected leaf area at day 8	LA08	cm^2^	0.82	0.48
Projected leaf area at day 10	LA10	cm^2^	0.84	0.44
Projected leaf area at day 12	LA12	cm^2^	0.81	0.43
Projected leaf area at day 14	LA14	cm^2^	0.76	0.41
Projected leaf area at day 16	LA16	cm^2^	0.73	0.39
Parameter a	PRA	cm^2^	0.51	0.24
Parameter k	PRK	1/day	0.28	0.00
Plant perimeter length	PER	cm	0.80	0.41
Plant major axis of the best fitting ellipse	MAJ	cm	0.82	0.43
Plant minor axis of the best fitting ellipse	MIN	cm	0.78	0.36
Plant aspect ratio: major axis/minor axis	ASR		0.56	0.17
Maximum plant diameter	MAD	cm	0.82	0.43
Minimum plant diameter	MID	cm	0.81	0.38
Plant circularity: 4 *π*(area/perimeter^2^)	CIR		0.33	0.09
Plant roundness: 4*area/(π*major axis^2^)	ROU		0.52	0.16
Plant solidity: area/convex area	SOY		0.52	0.14
Fresh mass	FHM	g	0.69	0.27
Dry mass	DYM	g	0.72	0.35
SPAD measurement	SPD		0.77	0.33
H_2_O content	H2O	% of fresh mass	0.39	0.27

### Plant material for weighted gene co-expression network analysis

The doubled haploid (DH) winter oilseed rape mapping population ExV8-DH which segregates for multiple seed quality, developmental and performance traits was the basis for the weighted gene co-expression network analysis (WGCNA). Pooled seedling developmental traits from 250 lines of the ExV8-DH population, described previously by Basunanda et al. [11], were measured in replicated greenhouse trials in 2007, and field trials at four locations from 2005-2007 were used to select two groups of 47 ExV8-DH lines with the highest and lowest respective mean performance for developmental and yield-related traits.

### Digital gene expression analysis

For digital gene expression analysis, the 94 pre-selected DH lines, the two parents Express 617 and V8, and their F_1_ (Express 617 x V8), were germinated in Jacobsen vessels under controlled conditions in a climate chamber at 20°C for 16 h (day) and 15°C for 8 h (night) with 55% relative humidity. Two experimental replications were performed. At two time points (eight and twelve days after sowing) 100 seedlings from each line were harvested for ribonucleic acid (RNA) extraction within one hour to prevent circadian clock effects during transcriptome analysis. All samples were immediately shock-frozen in liquid nitrogen and stored at -80°C until RNA extraction. Extraction of messenger RNA (mRNA) and digital gene expression sequencing (DGE-seq) was conducted on all as described by Obermeier et al. [12]. WGCNA was performed to identify gene networks correlated to developmental and yield-related traits. Within trait-correlated network modules, hub genes showing the highest interconnectivity to other genes in the module were selected as potential regulatory candidates for reverse transcription quantitative polymerase chain reaction (RT-qPCR) in thediversity set.

### RNA extraction, cDNA synthesis, and RT-qPCR

A total of 100 ng of the leaf apex of the second leaf of each of the 509 genotypes of each of the six replicates was collected after 30 days of growing in the greenhouse trial as explained in detail by Körber et al. [10]. After harvest, the sample was directly frozen in liquid nitrogen. The leaf samples were ground to a fine powder in liquid nitrogen. Total RNA was isolated from the fine powder using Trizol reagent following the manufacturer’s protocol (Invitrogen, Karlsruhe, Germany). The total RNA was treated with RNase-free DNase I (Fermentas) (final volume 100 *μ*l) to remove genomic deoxyribonucleic acid (DNA) contamination. RNA concentration was determined using the NanoDrop ND-1000 spectrophotometer (Thermo Fisher Scientific Inc., Waltham, MA, USA). All samples were diluted to an RNA concentration of 100 ng/ *μ*l and the samples from the six replicates of each inbred were pooled to equal amounts in order to reduce error variance. First-strand complementary DNA (cDNA) was synthesized from 15 *μ*l of total RNA using Maxima First Strand cDNA Synthesis Kit for RT-qPCR (Invitrogen, Karlsruhe, Germany) following the manufacturer’s recommendations. The resulting cDNA was diluted to 25 ng/ *μ*l. Gene-specific primers (10 pmol/ *μ*l) for 15 candidate genes as well as the control gene *Actin* (Table 2) were used for the RT-qPCRs performed on the cDNA samples. Amplifications were performed using 5 *μ*l of cDNA, 7 *μ*l of DyNAmo ColorFlash SYBR Green (Biozym), and 1.5 *μ*l of each primer. To minimize pipetting inaccuracy, the pipetting of the cDNA was done using the pipetting robot Biomek FX (Biomek). The following amplification conditions were used for the RT-qPCR on a LightCycler480 (Roche): Preincubation with 95°C for 3 min and amplification with 45 (*APL* = 55) cycles of 95°C (10 sec), and 60°C (1 min). At the end of each run, a dissociation analysis was performed to confirm the specificity of the reaction. In each 384-well plate used for RT-qPCR reaction, non-template controls and cDNA of the two trial standards were included. The RT-qPCR products of each of the 15 genes (eight from WGCNA (see below) and seven from literature) for five inbreds of the diversity set were Sanger sequenced at the Max Planck Genome Center Cologne to confirm the specific amplifications.

**Table 2:** **Details of 15 genes and the housekeeping gene** ***Actin*** **which were studied with qRT-PCR in seedling leaves harvested from the greenhouse trial in the 509** ***B. napus*** **inbreds**

**Abb. ** ^***a***^	**Gene name**	**Amplicon size**	**Organism ** ^***b***^	**Reference**	**Start position**	**Primer sequence**	**No. of qRT-PCR**
					**of primer sequence**		**cycles**
*CEL16*	Endo-1,4-beta-D-glucanase	112	*B. napus*	AJ242807.1	147	5‘-GGCTTCTGCATCCATTGTCT-3‘	45
	(cellulase)				258	5‘-TGCACTGTATCTGCCTCTCCT-3‘	
*FBP*	Fructose-1,6-bisphosphatase	111	*B. napus* mRNA	AF081796.1	81	5‘-GCCTCATCTCAGCCACAAAT-3‘	45
	precursor				191	5‘-AACCGCCATACACCTCACTC-3‘	
*SPS*	Sucrose-phosphate synthase	106	*B. rapa* subsp.	AY184484.1	194	5‘-CAGATGGGAACGAGGAACAT-3‘	45
			*pekinensis* mRNA		299	5‘-CTCGCAAGGGCAAGTATCAT-3‘	
*RBC*	Ribulose 1,5-bisphosphate carboxy-	96	*B. napus* mRNA	X07367.1	378	5‘-ACTACGATGGCCGTTACTGG-3‘	45
	lase/oxygenase small subunit				473	5‘-CCGTTTTGCACTCTTGGACT-3‘	
*PK*	Pyruvate kinase	89	*B. napus* mRNA	DQ539628.1	22	5‘-CCAAGGTTGTGGTGCTGAT-3‘	45
					110	5‘-CTGATGCGGTGATAATGGAAT-3‘	
*PECT*	Ethanolamine-phosphate	192	*A. thaliana* mRNA	NM_129424.4	483	5‘-CTGTGAAGTGGGTGGATGAA-3‘	45
	cytidylyltransferase				674	5‘-CAGTGCGCTTAATCTGCTTG-3‘	
*APL*	Glucose-1-phosphate adenylyl-	195	*A. thaliana* mRNA	NM_121927.3	581	5‘-CACTCAAACGCCAGGAGAAT-3‘	55
	transferase large subunit 1				775	5‘-CTTATATCCGCGCCACTCTG-3‘	
*AILP1*	Aluminum-induced protein	123	*B. napus* mRNA	JCVI_24	663	5‘-CTTGCTAAAAGGGGCTTGTG-3‘	45
					785	5‘-GCAGGAATGGCAGTGATCTT-3‘	
*GER1*	Germin-like protein subfamily 3	189	*B. napus* mRNA	JCVI_391	385	5‘-ATCACCGCTGGATTCATCTC-3‘	45
	member 1				573	5‘-AGCAAATAGCGCAAAGTCAAG-3‘	
*NOI*	nitrate-induced domain protein	198	*B. napus* mRNA	JCVI_10152	249	5‘-CCAGCATCAGCAGAAGGTTT-3‘	45
					446	5‘-TTGTGTCCGTCAAGAGTCCA-3‘	
*MyAP*	myrosinase-associated protein	131	*B. napus* mRNA	JCVI_1353	34	5‘-AAAATGGCACCCAACTTCAG-3‘	45
					164	5‘-TTGGAATCTCCGAATGTGAAC-3‘	
*GRF1*	Growth-regulating factor 1	101	*B. napus* mRNA	JCVI_41561	121	5‘-CTTGGAGTTCGGTTTGAAGG-3‘	45
					221	5‘-CCGGATCTTTCTTGCTTGTT-3‘	
*VPS2*	ESCRT ^*c*^ III complex	123	*B. napus* mRNA	JCVI_31511	148	5‘-TTTGCACCAGCAAGAGAGG-3‘	45
					270	5‘-GGCTTGTTTCATGTGTGACG-3‘	
*UBP15*	Ubiquitin carboxyl-terminal	117	*B. napus* mRNA	JCVI_5013	676	5‘-TGAGAGGCAACTGGTTCAGA-3‘	45
	hydrolase 15				792	5‘-TTAGAGGACGCGGATACGAT-3‘	
*GF14*	G box factor 14-3-3 omega	145	*B. napus* mRNA	JCVI_22791	621	5‘-TTGCCCATTCGCTTTTATTC-3‘	45
	protein				765	5‘-AAGGTTCGATGGCAGACACT-3‘	
*ACT*	Actin	104	*B. napus*	GQ339782	156	5‘-TCAGGCCGTTCTTTCTCTTTAC-3‘	45
					259	5‘-GAGCATAACCCTCGTAGATTGG-3‘	

^a^Abbreviation.

^b^Organism of the used reference sequence.

^c^Endosomal sorting complex required for transport.

### Genotyping of SNP markers

For the GWAS, the 509 *B. napus* inbred lines were assayed at Agriculture and Agri-Food Canada using a customized *Brassica napus* 6K Illumina Infinium SNP array (http://aafc-aac.usask.ca/ASSYST/). This array was designed from next generation sequence (NGS) data from Illumina short read (100 bp paired-end) genomic sequence data from seven *B. napus* cultivars and three *B. rapa* cultivars, from 3’ captured cDNA Roche 454 sequence data from seven *B. napus* cultivars and four *B. oleracea* cultivars as well as Illumina short read (80 bp single-end) RNA-Seq data from 42 *B. napus* cultivars [13]. It contained 5,506 successful bead types representing the same number of potential SNPs. Samples were prepared and assayed as per the Infinium HD Assay Ultra Protocol (Infinium HD Ultra User Guide 11328087_RevB, Illumina, Inc. San Diego, CA). The Brassica 6K BeadChips were imaged using an Illumina HiScan system, and the SNP alleles were called using the Genotyping Module v1.9.4, within the GenomeStudio software suite v2011.1 (Illumina, Inc. San Diego, CA). SNP data were available for 505 inbreds of the diversity set and only SNPs with a percentage of missing data < 30% across all genotypes and a minor allele frequency > 0.05 as well as genotypes with a percentage of missing data < 20% across all SNPs were used for the following statistical analysis. From these 3,910 SNPs, 3,828 could be assigned to a physical map position derived from the reference information of *B. rapa* [14] and *B. oleracea* [15].

### Statistical analyses

#### Weighted gene co-expression network analysis

WGCNA was performed using the WGCNA R package as described by Langfelder and Horvath [16]. Normalized tagcounts (per ten million reads) were obtained for 154,790 probes (86,908 probes mapping to *B. rapa* and 67,882 probes to *B. oleracea* reference unigene sequences) using Illumina sequencing of 3’EST digital gene expression tags. Probes were kept if they had a normalized tagcount of at least five in six or more samples. Replicate probes for each unigene were averaged and the 91,048 unigenes present in both datasets were used for the WGCNA consensus analysis. A total of 108 modules were obtained using the automatic network construction function “blockwiseConsensusModules” with the following settings; power = 5, minModuleSize = 50, deepSplit = 2, maxBlockSize = 35000, reassignThreshold = 0, mergeCutHeight = 0.25, minKMEtoJoin = 1, minKMEtoStay = 0. Using the WGCNA function “chooseTopHubInEachModule”, the top hub unigenes were identified from 15 modules which were highly conserved between the two datasets and eight of these top hub unigenes could be amplified as functional candidate genes by RT-qPCR in the 509 rapeseed inbred lines.

The network of unigenes with an edge weight of ≥ 0.1 was visualized in Cytoscape [17] and the function of the modules position was determined using Gene Ontology Singular Enrichment Analysis (p < 0.001) [18].

#### Normalization and differences of gene expression data

The C _*p*_-value for which the fluorescence rose above the background fluorescence was calculated for each inbred-gene combination using the LightCycler 480 Software (Roche; version 1.5). The C _*p*_-value, which was designated in the following as gene expression level of the different genes, was normalized to the percentage of the expression level of the housekeeping gene *Actin* for the corresponding inbred.

Associations among inbreds and genes were revealed by a heatmap analysis and grouped with the complete linkage clustering method.

#### Genome positions of the candidate genes

A basic local alignment search tool (BLAST) search [19] was performed between the reference sequences of the candidate genes and the reference sequences of *B. rapa* (v1.2) [14] and *B. oleracea* (v1) [15]. All positions were used which had a BLAST identity ≥ 85%.

#### Calculation of adjusted entry means

The adjusted entry mean *M* of each genotype-trait/-gene combination, which was the basis for all further analyses, were calculated for the seedling development traits and the gene expression data using different mixed-models. For the former, these were calculated as described in detail by Körber et al. [10]. The calculations for the gene expression data were based on the following model:

$$y_{ij} = {\mu + g_{i} + t_{j} + e_{ij},} $$

where *y*_*ij*_ was the observation of the *i*th genotype of the *j*th technical replication, *μ* an intercept term, *g*_*i*_ the genotypic effect of the *i*th genotype, *t*_*j*_ the effect of the *j*th technical replicate, and *e*_*ij*_ the residual. For calculating the adjusted entry means, *g*_*i*_ was regarded as fixed and all other effects as random.

#### Principal component analysis and the assessment of linkage disequilibrium

The 509 rapeseed inbreds of our study were assigned to three clusters (MCLUST) using a principal component analysis (PCA) of 89 SSR markers as described by Buset al. [2].

In order to determine the physical map distance in which LD decays in our *B. napus* diversity set, *r*^2^ (the square of the correlation of the allele frequencies between all pairs of linked SNP loci) was calculated, where linked loci were defined as loci located on the same chromosome, and plotted against the physical distance in megabase pairs. The overall decay of LD was evaluated by nonlinear regression of *r*^2^ according to Hill and Weir [20]. The percentage of linked loci in significant LD was determined with the significance threshold of the 95% quantile of the *r*^2^ value among unlinked loci pairs, where unlinked loci were defined as loci located on different chromosomes. Pairwise modified Roger’s distance (MRD) estimates between all inbreds and the MCLUST groups 1-3 were calculated according to Wright [21].

#### Genome-wide association analyses

The genome-wide association analyses of the seedling development traits and the gene expression data were performed as an single marker analysis using the *PK* method [22]:

$$M_{lm} = \mu + a_{m} + \sum_{u=1}^{z} P_{lu} v_{u} + g_{l}^{*} + e_{lm}, $$

where *M*_*lm*_ was the adjusted entry mean of the *l*th inbred carrying allele *m*, a _*m*_ the effect of the *m*th allele, *v *_*u*_ the effect of the *u*th column of the population structure matrix *P*, $g_{l}^{*}$ the residual genetic effect of the *l*th entry, and *e*_*lm*_ the residual. The first and second principal component calculated based on the 89 SSR markers [2] were used as *P* matrix. The variance of the random effect $g^{*} = \left \{g^{*}_{1},...,g^{*}_{509}\right \}$ was assumed to be Var($g^{*}) = 2K\sigma _{g^{*}}^{2}$, where $\sigma _{g^{*}}^{2}$ was the residual genetic variance. The kinship coefficient *K*_*ij*_ between inbreds *i* and *j* were calculated based on the above mentioned SSR markers according to:

$$ K_{ij} = \frac{S_{ij-1}}{1 + T} + 1, $$

where *S *_*ij*_ was the proportion of marker loci with shared variants between inbreds *i* and *j* and *T* the average probability that a variant from one parent of inbred *i* and *a* variant from one parent of inbred *j* are alike in state, given that they are not identical by descent [23]. The optimum *T* value was calculated according to Stich et al. [22] for each trait. To perform the above outlined association analysis, the R package EMMA [24] was used. We chose the significance threshold of *P*-value =0.0001 and the threshold after Bonferroni correction (*P*-value =0.05). The association analysis was performed for all inbreds and for each of the three MCLUST groups. For the separate association analyses of the three MCLUST groups, only the kinship matrix *K* but no *P* matrix was considered. SNPs which are associated for multiple traits are defined as hot spots for these traits.

If not stated differently, all analyses were performed with the statistical software R [25].

## Results

### Linkage disequilibrium and allele frequency

**Figure 1:**
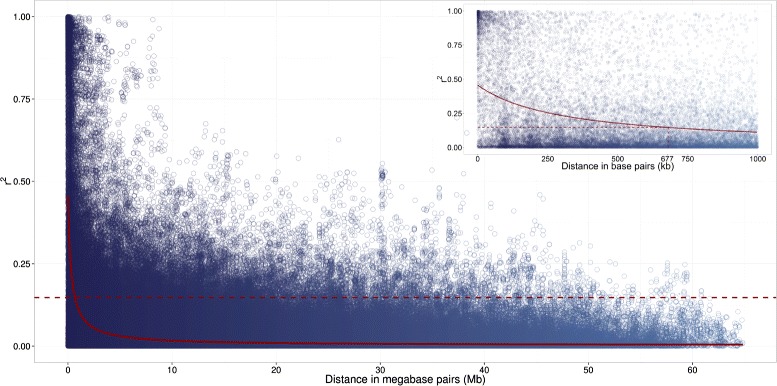
Linkage disequilibrium of the *B. napus* diversity set. Plot of linkage disequilibrium measured by squared allele frequency correlations (*r* ^2^, dots) versus physical map distance (Mb) between linked single nucleotide polymorphism (SNP) marker loci in the *B. napus* diversity set. The solid line represents the nonlinear regression trend line of *r* ^2^ versus the physical map distance, whereas the dashed line indicates the threshold of the 95% quantile of *r* ^2^ between unlinked loci pairs. The inset gives an enhanced view of the *r* ^2^ decay over smaller physical map distances (kb).

The nonlinear regression trend line of the LD measure *r*^2^ vs. the physical distance intersected the Q_95_ of *r*^2^ among unlinked loci pairs (0.145) at 676,992 bp (Figure 1). The allele frequencies of the 3,828 SNPs of all 509 inbreds ranged from 0.05 to 0.95.

### Gene expression data

The expression levels of the 15 candidate genes examined in the 509 *B. napus* inbreds showed an averaged standard deviation (SD) of 5.6 across all inbreds and ranged from 3.2 to 8.8. The average MRD (±standard error) of the MCLUST groups 1 to 3 vs. the other two MCLUST groups were 0.32 (±0.01), 0.34 (±0.01), and 0.28 (±0.01), respectively.

**Figure 2:**
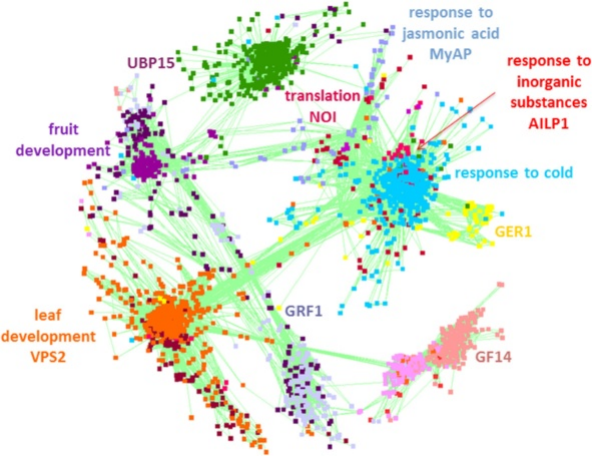
Co-expression network. Co-expression correlation network of 3340 genes for the 8DAS dataset showing the relationship of the modules in different colors and the names of the eight regulatory candidate genes. The position of the eight candidate genes is shown in the network together with the function of each module.

The consensus WGCNA for the two datasets allocated 83,262 unigenes into 108 modules, where 7,776 unigenes were unassigned. Each module comprised between 53 and 10,285 unigenes. The candidate genes were selected as the top hub genes from 15 modules which were highly conserved between the two datasets, and for eight of them amplification via qRT-PCR was successful (Figure 2). Seven further candidate genes were selected from main metabolic pathways.

**Figure 3:**
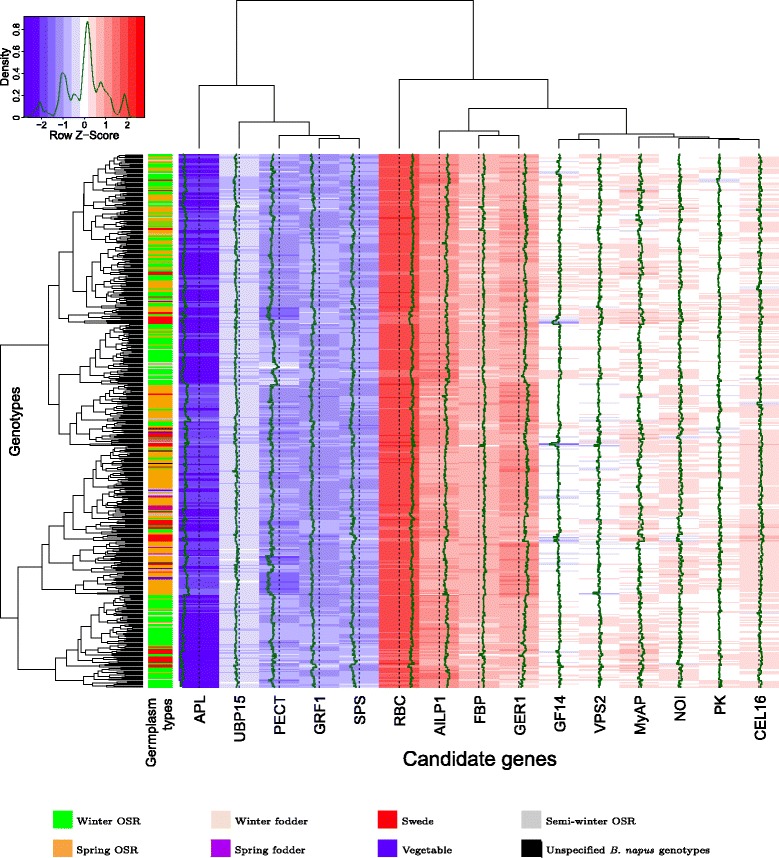
Heatmap of the expression levels. Heatmap of the expression levels of the studied candidate genes in seedling leaves of *B. napus* relative to the expression levels of the housekeeping gene *Actin* for the 509 inbreds of the diversity set. On the x-axis, the 15 candidate genes are plotted and the y-axis shows the 509 *B. napus* inbreds with their corresponding germplasm type. The dendrogram of the 509 *B. napus* inbreds is based on the gene expression data. Genes with a blue mark have an expression level lower than the expression levels of the housekeeping gene *Actin* and red marked genes have an higher expression level.

Across the examined 15 candidate genes, the gene *APL* was expressed on average lowest relative to *Actin*, whereas the gene *RBC* was expressed highest (Figure 3). The genes *APL*, *UBP15*, *PECT*, *GRF1*, and *SPS* were assigned to a cluster of genes which had a lower expression compared to *Actin*, whereas all the other genes clustered to a group of highly expressed genes. Furthermore, based on the expression levels of the 15 genes, the 509 inbreds were clustered in five different subgroups comprising different germplasm types.

**Figure 4:**
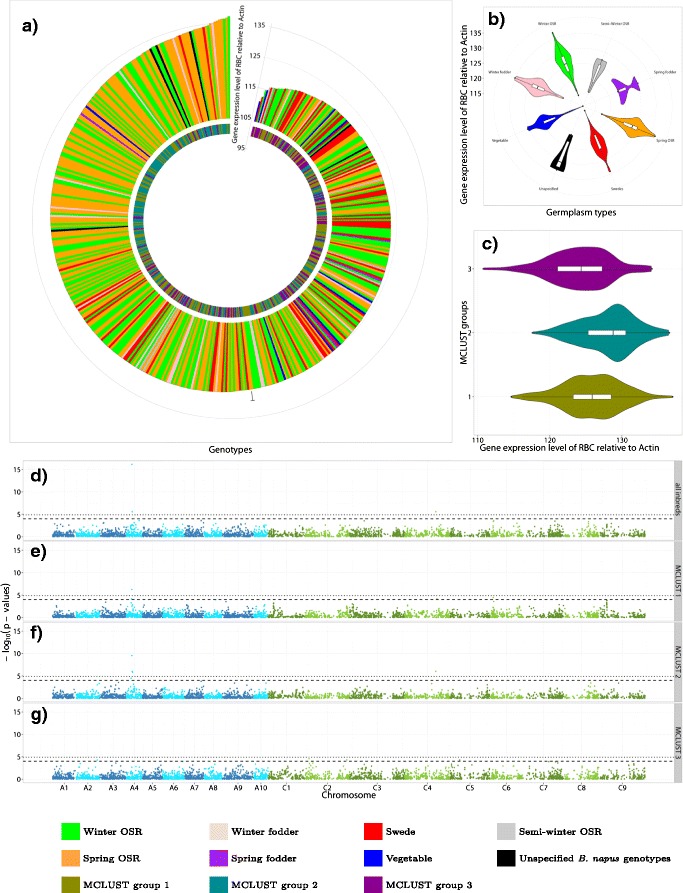
Candidate gene ribulose 1,5-bisphosphate carboxylase/oxygenase small subunit.**(a)** Distribution of the expression level of the gene *RBC* relative to the housekeeping gene *Actin* across all 509 inbreds ordered by the gene expression level. **(b)** Violinplot of the gene expression level of *RBC* for the eight different germplasm types and **(c)** for the three MCLUST groups. **(d)** *P*-value profile from genome-wide association mapping for the gene expression level of the *RBC* gene for all 509 inbreds, **(e)** for the inbreds of the MCLUST group 1, **(f)** for the inbreds of the MCLUST group 2, and **(g)** for the inbreds of the MCLUST group 3. The x-axis shows physical map positions of the SNPs along the 19 chromosomes, the y-axis gives the -log_10_ *P*-value of the association test. The horizontal dashed and dotted lines indicate the *P*-value = 0.0001 threshold and the threshold after Bonferroni correction (*P*-value = 0.05), respectively.

The expression levels of the analysed genes differed between the eight germplasm subsets and the three MCLUST groups. Across all 509 inbreds, the expression levels of the genes *FBP*, *SPS*, *RBC*, *PK*, *UBP15*, *PECT*, *APL*, *AILP1*, *GER1*, *NOI*, *GRF1*, and *GF14* were significantly higher (*P*-value = 0.05) in the mainly modern winter OSR and spring OSR germplasm types compared to the remaining subsets. In contrast, the expression levels of the genes *CEL16* and *MyAP* showed the opposite trend (Figure 4a-c and Additional file 6: Figure S1a-c - 14a-c). The genes *SPS*, *UBP15*, *PECT*, *AILP1*, *MyAP*, *GRF1*, *VPS2*, and *GF14* were significantly (*α* = 0.05) higher expressed in the inbreds of the MCLUST group 1 than in the inbreds of the MCLUST groups 2 and 3. On the other hand, the genes *FBP*, *RBC*, *APL*, *GER1*, and *NOI* were significantly higher expressed in the inbreds of the MCLUST group 2 and the genes *CEL16* and *MyAP* for the inbreds of the MCLUST group 3 (Figure 4a-c and Additional file 6: Figure S1a-c - 14a-c).

The absolute value of the correlation coefficient between the expression of the 15 candidate genes with the 20 seedling development traits for all 509 inbreds was on average 0.11 with a range from 0.00 to 0.39 (MCLUST 1-3: 0.09, 0.13, and 0.10). The candidate genes *GER1* and *FBP* were mostly negatively correlated with the seedling development traits with a correlation coefficient down to -0.39. In contrast, the candidate genes *AILP1* and *PECT* were mostly positively correlated with the seedling development traits with a correlation coefficient up to 0.26 (Additional file 8: Figure S35–38).

### Genome-wide association mapping

In the GWAS with 3,910 SNPs for all 509 *B. napus* inbreds, we observed a total of 63 SNP-trait associations with a *P*-value < 0.0001 for 14 of the 20 seedling development traits. A total of 20.6% of these SNP-trait associations were detected for the A genome and more than half of them were located on the chromosomes A10 and A3. In contrast, 76.2% of the associations were detected for the C genome and most of them were located on the chromosomes C7 and C2. In addition, two SNP-trait associations could not be mapped to the genome of *B. napus* (Table 3). The 63 associations explained individually from 3.0 to 4.9% of the phenotypic variance. Furthermore, between one and 21 SNP-trait associations were associated with the same trait and these explained in simultaneous fits between 3.3 and 20.3% of the phenotypic variance (Table 3).

**Table 3:** **Single nucleotide polymorphism (SNP)-trait/-gene associations with** ***P*** **< 0.0001 across all inbreds**

**Trait**	**SNP array code**	**Chr. ** ^***a***^	**Position (bp)**	**Allele 1/2**	***P*** ** value**	**Effect allele**	***P***_***V***_^***b***^ ** (%)**
						**1/2**	
*APL*	Bn-ctg7180014739201-p14151	C3	23542236	C/T	1.59e-05	4.16	4.10
	Bn-ctg7180014739201-p15057	C3	23543142	G/A	3.63e-05	3.93	3.54
Simultaneous fit						3.56	
*CEL16*	Bn-Scaffold000020-p837037	A8	18471964	G/A	3.21e-05	2.45	3.52
*FBP*	Bn-ctg7180014767990-p1760	A9	27053307	G/T	9.56e-05	1.33	2.96
	p8_2822_snp11	C2	2008764	G/A	3.33e-05	1.56	3.89
	Bn-ctg7180014769003-p8702	C7	23881203	C/A	8.25e-07	-1.88	5.56
	Bn-ctg7180014750397-p3915	C8	28994818	G/A	7.42e-05	1.56	3.37
	Bn-ctg7180014767990-p1760	C8	29012996	G/T	9.56e-05	1.33	2.96
	Bn-ctg7180014767990-p516	C8	29014240	C/T	8.61e-05	-1.30	3.03
Simultaneous fit						10.37	
*GER1*	Bn-ctg7180014741426-p3290	C1	40791705	C/A	3.44e-05	2.54	3.53
	Bn-ctg7180014744563-p4387	C2	45592086	C/T	6.28e-06	2.43	4.38
	Bn-ctg7180014744563-p4646	C2	45592345	G/A	2.92e-06	2.54	4.72
Simultaneous fit						7.50	
*GRF1*	Bn-Scaffold000031-p1055544	A2	24176291	C/T	2.68e-05	4.44	3.58
*MyAP*	Bn-Scaffold000033-p1183872	A1	23083128	G/A	4.32e-05	2.54	3.46
	p5_8783_snp47	C2	16695448	G/A	3.99e-05	-4.28	4.01
	Bn-ctg7180014755468-p3376	C5	45992213	G/A	4.66e-05	-3.04	3.60
Simultaneous fit						9.66	
*NOI*	Bn-ctg7180014731124-p2983	C9	52425308	C/A	7.91e-06	-3.30	4.92
*PECT*	Bn-ctg7180014744894-p12663	C4	5903754	G/T	2.75e-05	3.95	3.49
	Bn-ctg7180014772336-p17139	C7	38391732	C/A	2.93e-05	5.11	3.62
Simultaneous fit						7.03	
*PK*	Bn-Scaffold000031-p1055544	A2	24176291	C/T	5.64e-05	2.55	3.29
	Bn-Scaffold000005-p1543856	A3	1543856	G/A	9.96e-05	1.85	3.14
	p5_8783_snp47	C2	16695448	G/A	9.42e-05	-2.83	3.63
	Bn-ctg7180014730936-p7448	C7	43801848	G/T	6.03e-05	-2.36	3.25
Simultaneous fit						10.04	
*RBC*	Bn-Scaffold000060-p721650	A4	6466112	C/T	7.19e-17	-3.36	13.53
	BN051924-0307	A4	6928299	C/T	2.66e-06	-2.67	4.46
	BN051924-0307	C4	36645724	C/T	2.66e-06	-2.67	4.46
Simultaneous fit						13.68	
*SPS*	BN043454-0504	A9	27982958	G/A	4.52e-05	2.88	3.36
	p5_8783_snp47	C2	16695448	G/A	8.09e-05	-4.10	3.68
Simultaneous fit						6.57	
*UBP15*	Bn-Scaffold000003-p3164670	A7	14616879	G/T	9.71e-06	2.34	3.93
*VPS2*	Bn-Scaffold000004-p4962830	A5	4962830	G/A	8.30e-05	-5.07	3.13
	Bn-ctg7180014738809-p1661	C9	46132950	C/T	6.46e-05	-2.76	3.22
Simultaneous fit						5.95	
*ASR*	UQ10A0010802	A10	11497630	C/T	7.41e-05	-0.10	3.67
	Bn-ctg7180014733191-p16993	C2	11324627	G/A	8.80e-05	-0.10	3.02
	Bn-ctg7180014737642-p1736	C2	11521447	C/T	4.55e-05	-0.11	3.48
	Bn-ctg7180014722959-p1362	C2	11538841	G/A	7.27e-05	-0.10	3.08
	Bn-ctg7180014722959-p3680	C2	11541159	C/T	9.48e-05	0.10	3.62
	Bn-ctg7180014761021-p763	C2	11545317	G/T	8.56e-05	0.10	3.00
	Bn-ctg7180014761021-p1313	C2	11545867	G/T	6.38e-05	0.11	3.82
	Bn-ctg7180014746007-p654	C2	11575760	G/A	8.93e-05	-0.11	3.86
	Bn-ctg7180014747519-p4930	C2	11581331	C/A	6.89e-05	-0.10	3.77
	Bn-ctg7180014733329-p2936	C2	50724670	C/T	5.31e-05	0.08	3.45
	Bn-ctg7180014744693-p3728	C3	13670419	C/T	3.76e-05	0.09	3.77
	Bn-ctg7180014758876-p10173	C5	41416666	C/T	4.60e-05	0.08	3.67
	Bn-ctg7180014715862-p5107	C7	20535180	C/T	5.26e-06	0.09	4.57
	Bn-ctg7180014711252-p832	C7	20577577	G/A	1.82e-05	0.08	3.78
	Bn-ctg7180014711906-p3935	C7	21407038	C/T	3.44e-05	0.10	4.21
	Bn-ctg7180014711906-p4070	C7	21407173	C/T	2.12e-05	0.10	4.42
	Bn-ctg7180014769003-p8702	C7	23881203	C/A	9.20e-05	0.09	3.42
	Bn-ctg7180014760120-p14495	C7	37894126	G/A	8.06e-05	-0.08	4.23
	Bn-ctg7180014765154-p10081	C9	1282177	C/T	1.57e-05	-0.08	4.28
	Bn-ctg7180014758656-p7160	C9	2835355	C/A	2.53e-06	0.09	4.87
	Bn-ctg7180014722956-p4266	Scaffold01930	3005	G/T	3.40e-05	-0.11	3.63
Simultaneous fit						20.31	
*CIR*	UQ10A0010802	A10	11497630	C/T	1.75e-05	0.03	4.16
	BN064849-0420	C5	12385920	C/T	5.14e-05	0.04	3.32
Simultaneous fit						7.65	
*H2O*	Bn-Scaffold000005-p1543856	A3	1543856	G/A	7.66e-05	0.31	3.68
*LA08*	UQ10C0016384	C7	32496339	C/T	4.04e-06	-0.31	4.34
*LA10*	UQ10C0016384	C7	32496339	C/T	2.18e-06	-0.60	4.46
*LA12*	UQ10C0016384	C7	32496339	C/T	4.16e-05	-0.83	3.36
*MAD*	BN054115-0054	A3	2852962	G/A	8.42e-05	0.27	3.79
	BN054115-0054	C3	3505618	G/A	8.42e-05	0.27	3.79
	UQ10C0016384	C7	32496339	C/T	3.16e-05	-0.23	3.44
Simultaneous fit						6.75	
*MAJ*	BN054115-0054	A3	2852962	G/A	5.11e-05	0.26	3.96
	Bn-Scaffold000004-p5075668	A5	5075668	C/T	8.74e-05	0.23	3.40
	Bn-Scaffold000058-p205581	A9	9133931	G/A	7.34e-05	0.19	3.27
	BN054115-0054	C3	3505618	G/A	5.11e-05	0.26	3.96
	UQ10C0016384	C7	32496339	C/T	5.59e-05	-0.21	3.24
Simultaneous fit						10.14	
*MID*	UQ10C0016384	C7	32496339	C/T	5.63e-05	-0.13	3.25
*PER*	UQ10C0016384	C7	32496339	C/T	1.93e-05	-0.93	3.57
*PRA*	UQ10C0204660	C4	12991242	C/T	3.28e-05	-0.08	3.64
*ROU*	UQ10A0010802	A10	11497630	C/T	1.49e-05	0.04	4.46
	Bn-ctg7180014761021-p1313	C2	11545867	G/T	9.46e-05	-0.04	3.83
	Bn-ctg7180014715862-p5107	C7	20535180	C/T	1.02e-05	-0.03	4.38
	Bn-ctg7180014711252-p832	C7	20577577	G/A	4.92e-05	-0.03	3.39
	Bn-ctg7180014711906-p3935	C7	21407038	C/T	3.21e-05	-0.04	4.57
	Bn-ctg7180014711906-p4070	C7	21407173	C/T	2.00e-05	-0.04	4.79
	Bn-ctg7180014765154-p10081	C9	1282177	C/T	1.78e-05	0.03	4.45
	Bn-ctg7180014758656-p7160	C9	2835355	C/A	1.49e-05	-0.03	4.44
	Bn-ctg7180014722956-p4266	Scaffold01930	3005	G/T	9.35e-05	0.03	3.33
Simultaneous fit						13.23	
*SOY*	Bn-Scaffold000024-p2080233	A6	10852718	G/T	7.56e-05	0.02	3.42
	UQ10A0010802	A10	11497630	C/T	4.70e-05	0.02	3.99
	Bn-ctg7180014715862-p5107	C7	20535180	C/T	4.27e-06	-0.02	4.48
	Bn-ctg7180014711252-p832	C7	20577577	G/A	6.05e-06	-0.02	4.21
	Bn-ctg7180014760120-p14233	C7	37893864	C/T	2.33e-05	0.02	3.78
	Bn-ctg7180014760120-p14495	C7	37894126	G/A	3.73e-05	0.02	4.53
	Bn-ctg7180014765154-p10081	C9	1282177	C/T	5.69e-05	0.02	3.76
Simultaneous fit						14.07	
*SPD*	Bn-Scaffold000034-p1847442	A1	24476947	G/A	9.46e-05	1.68	3.21
	Bn-Scaffold000017-p525526	A7	5241142	C/T	2.47e-05	-1.11	3.61
	snp_BGA_4772	A10	11407615	C/T	8.35e-05	2.47	3.88
	Bn-ctg7180014759380-p14755	C1	1310534	G/A	1.84e-05	-1.96	4.12
	p5_8783_snp47	C2	16695448	G/A	2.40e-05	1.88	4.21
	Bn-ctg7180014762070-p3737	C5	1112869	C/T	4.52e-05	-1.15	3.36
	Bn-ctg7180014771511-p3122	C5	40979968	C/T	3.38e-05	-1.37	3.44
	Bn-ctg7180014748477-p7622	C8	19334419	G/T	4.00e-05	-1.71	3.38
	Bn-ctg7180014710293-p19591	C8	19418083	C/T	4.89e-05	-1.83	3.43
Simultaneous fit						20.10	

For abbreviations of the traits see Tables 1 and 2.

^a^Chr. is the chromosome of the respective SNP.

^b^ *P*_V_ is the proportion of the explained phenotypic variance.

**Figure 5:**
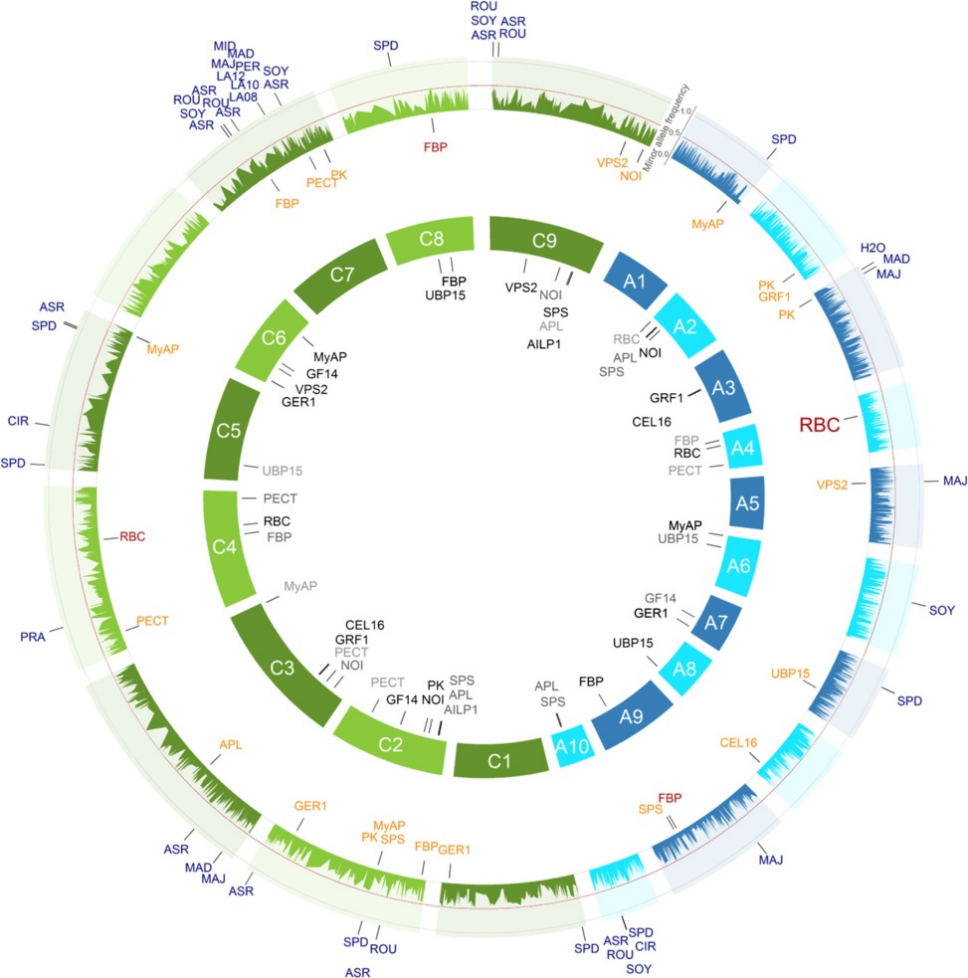
GWAS: SNP-trait/-gene associations of all 509 inbreds of the *B. napus* diversity set. All single nucleotide polymorphism (SNP)-trait/-gene associations with a *P*-value < 0.0001 identified across all 509 inbreds and their respective positions are marked on the *B. napus* genome. The 3,828 SNPs with their minor allele frequencies are given in the outer circle. The SNPs associated with the candidate gene expression based on the gene expression data are plotted in orange below the allele frequency circle and the seedling development SNP-trait associations in blue outside the allele frequency circle. The size of the letters is related to the proportion of the variance explained by the associations. In the inner circle of the 19 chromosomes, the candidate genes were plotted to their mapping position on the *B. rapa* and *B. oleracea* reference genomes. Potential *cis*-regulated candidate genes were colored red. The A genome is colored blue and the C genome green.

For the association analysis of the gene expression levels, we observed across all 509 *B. napus* inbreds 31 SNP-gene associations for 13 of the 15 examined genes with a *P*-value < 0.0001. A total of 35.5% of these SNP-gene associations were located on the A genome, whereas no clustering across the chromosomes was observed. In contrast, 64.5% were identified for the C genome and 40% of them were located on the chromosomes C2 and C8 (Figure 5 and Table 3). We identified between one and six SNPs to be associated with the gene expression variation of the individual genes. The identified SNPs explained individually from 3.0 to 13.5% of the phenotypic variance. Furthermore, between two and seven SNP-gene associations were associated with the same gene and these explained in simultaneous fits between 3.6 to 13.7% of the phenotypic variance (Table 3).

Across all 509 inbreds, the SNP-*FBP* association of the gene expression levels was identical with the SNP-*ASR* association of the seedling development traits on chromosome C7. The SNP-gene associations of *MyAP*, *PK*, and *SPS* and the SNP-trait association of *SPD* on chromosome C2 as well as the SNP-gene association of *PK* and the SNP-trait association of *H2O* on chromosome A3 were also identical for all 509 inbreds. Furthermore, for the MCLUST group 2 the SNP-gene association of *PECT* on chromosome C6 corresponded with the associations of the projected leaf area hot spot of the seedling development traits.

### Correspondence of associations across subgroups

In the *P*-value profile from the genome-wide association mapping, several SNP-*RBC* associations with a *P*-value < 0.0001 were detected on chromosome A4 for all 509 inbreds and the inbreds of the MCLUST groups 1-2 (Figure 4d-f) as well as on chromosome C4 for all 509 inbreds and the inbreds of the MCLUST group 2 (Figure 4d and f). Furthermore, mentionable SNP-*RBC* associations with a *P*-value < 0.0001 were observed on chromosome C6 for the inbreds of the MCLUST group 1 and on chromosome C2 for the inbreds of the MCLUST group 3 (Figure 4e and g).

**Figure 6:**
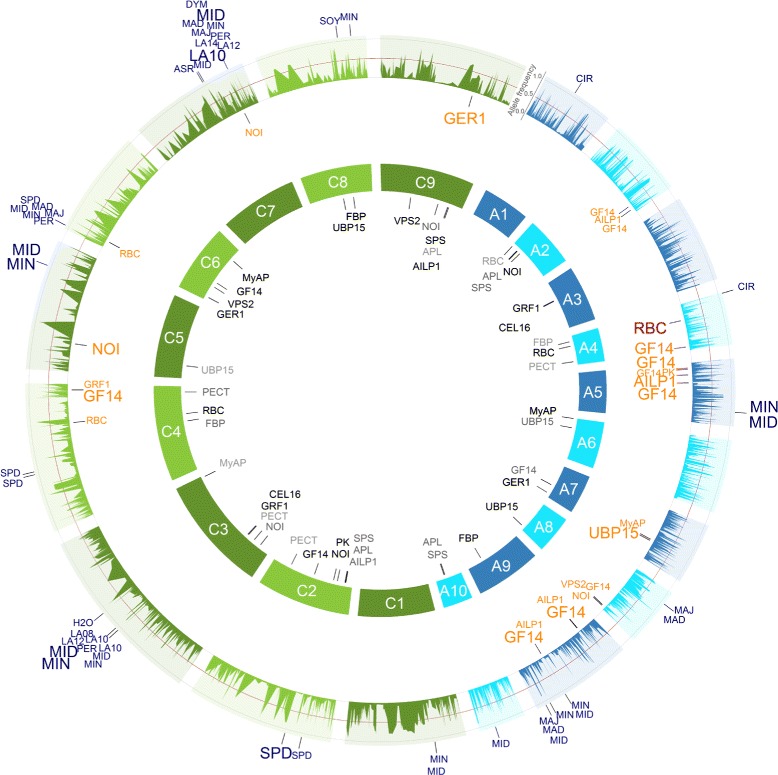
GWAS: SNP-trait/-gene associations of the inbreds of the MCLUST groups 1 of the *B. napus* diversity set. All single nucleotide polymorphism (SNP)-trait/-gene associations with a *P*-value < 0.0001 identified across the inbreds of the MCLUST groups 1 and their respective positions are marked on the *B. napus* genome. The 3,828 SNPs with their frequencies of the allele being the minor allele in the 509 inbreds are given in the outer circle. The SNPs associated with the candidate gene expression based on the gene expression data are plotted in orange below the allele frequency circle and the seedling development SNP-trait associations in blue outside the allele frequency circle. The size of the letters is related to the proportion of the variance explained by the associations. In the inner circle of the 19 chromosomes, the candidate genes were plotted to their mapping position on the *B. rapa* and *B. oleracea* reference genomes. Potential *cis*-regulated candidate genes were colored red. The A genome is colored blue and the C genome green.

**Figure 7:**
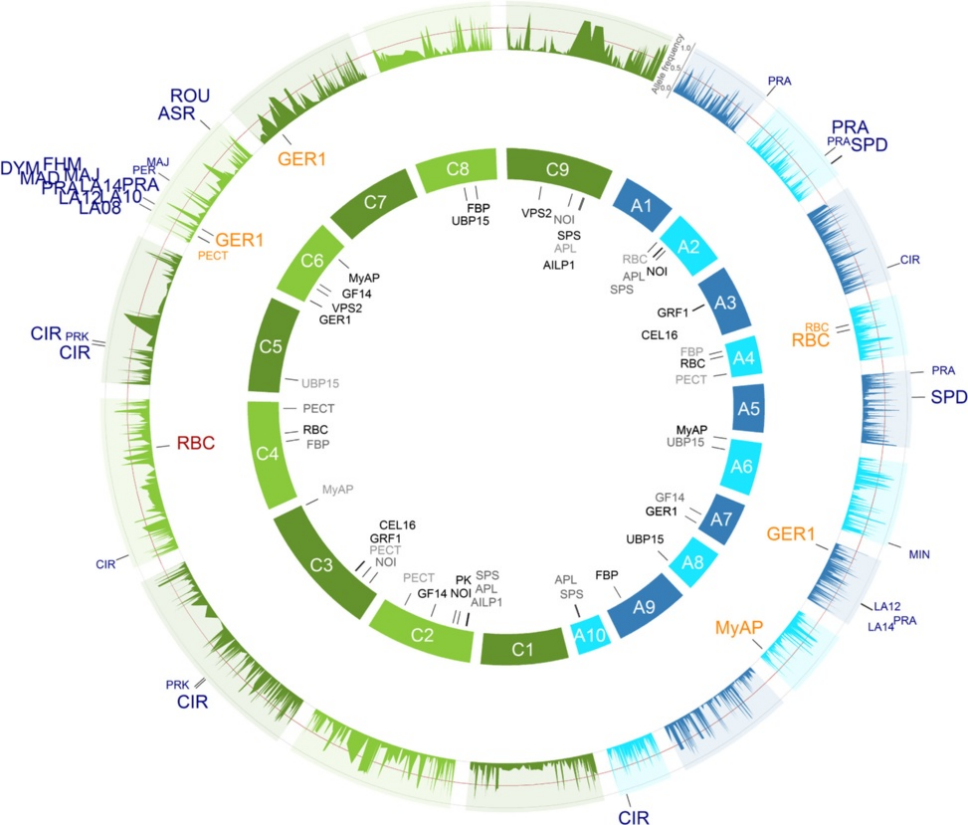
GWAS: SNP-trait/-gene associations of the inbreds of the MCLUST groups 2 of the *B. napus* diversity set. All single nucleotide polymorphism (SNP)-trait/-gene associations with a *P*-value < 0.0001 identified across the inbreds of the MCLUST groups 2 and their respective positions are marked on the *B. napus* genome. The 3,828 SNPs with their frequencies of the allele being the minor allele in the 509 inbreds are given in the outer circle. The SNPs associated with the candidate gene expression based on the gene expression data are plotted in orange below the allele frequency circle and the seedling development SNP-trait associations in blue outside the allele frequency circle. The size of the letters is related to the proportion of the variance explained by the associations. In the inner circle of the 19 chromosomes, the candidate genes were plotted to their mapping position on the *B. rapa* and *B. oleracea* reference genomes. Potential *cis*-regulated candidate genes were colored red. The A genome is colored blue and the C genome green.

The SNP-*RBC* associations detected on chromosome A4 and C4 for all 509 *B. napus* inbreds and the inbreds of the MCLUST group 2 were in accordance with their physical map position. Furthermore, for the inbreds of the MCLUST group 1 the SNP-*RBC* association on chromosome A4 was also in accordance with its physical map position, but not on chromosome C4, where the distance in between was ~1.3 Mb. In addition, the SNP-*FBP* associations were in accordance with their mapped genome positions on chromosome A9 and C8 for all inbreds (Figures 5, 6 and 7).

## Discussion

### Linkage disequilibrium and SNP density

The nonlinear trend line of LD measure *r*^2^ decayed below the significance threshold, the 95% quantile of the *r*^2^ value among unlinked loci pairs, within a distance of 677 kb (Figure 1). Bus et al. [2] estimated based on 89 SSR markers that the pairwise LD decayed within a genetic map distance of approximately 1 cM. This corresponds to about 500 kb [26] and is in good accordance to the value observed in our study. The LD observed by Ecke et al. [27] decayed within 2 cM less fast. The reason for this observation could be that the population studied by Ecke et al. [27] was less diverse than the *B. napus* diversity set examined in the current study.

In our study, 1,755 SNPs mapped to the A genome, whereas 2,073 SNPs mapped to the C genome. Furthermore, 99.4% of the adjacent SNPs on the A genome and 93.0% of the adjacent SNPs on the C genome had a distance smaller than the average range of LD (677 kb). Therefore, this GWAS is expected to result on average in 14.7% of the possible power (Figure 1). Compared to previous studies in *B. napus*, the SNP marker density of our study is expected to provide a higher power to detect SNP-trait/-gene associations in the *B. napus* diversity set.

### Genome-wide association mapping of seedling development traits

Seedling development traits are important targets for breeding because an optimal seedling development leads to a higher yield stability even under suboptimal growing conditions [1]. Up to now, however, little is known about the genetic mechanisms as well as the natural variation of seedling development in *B. napus*. Thus, we used an association mapping approach to elucidate the genetics of seedling development in *B. napus*.

We observed a total of 63 associations between SNPs and 14 of the 20 seedling development traits with a *P*-value < 0.0001 (Figure 5 and Additional file 7: Figure S15d-g - 34d-g). Furthermore, for the 14 seedling development traits we found between one and 21 SNP-trait associations for a single trait which explained in a simultaneous fit, on average, 8.5% of the phenotypic variance with a range from 3.3 to 20.3% (Table 3). The large number of associations for these 14 seedling development traits suggests that these are genetically complex inherited. In contrast to the seedling development traits examined in our study, Honsdorf et al. [7] carried out an association analysis of phenological, morphological, and quality traits in 84 canola quality winter rapeseed (*Brassica napus*) and identified 86 putative QTLs for ten of 14 traits which explained, on average, 36.2% of the phenotypic variance. These differences in the explained phenotypic variance could be due to the fact that Honsdorf et al. [7] analysed agronomic and seed quality traits instead of seedling development traits and that a lower number of genotypes were examined compared to our study. The latter leads to an overestimation of marker effects. This overestimation, however, decreases with a higher number of genotypes in a GWAS [28]. Thus, in a GWAS with 509 inbreds this overestimation is expected to be of minor importance.

**Figure 8:**
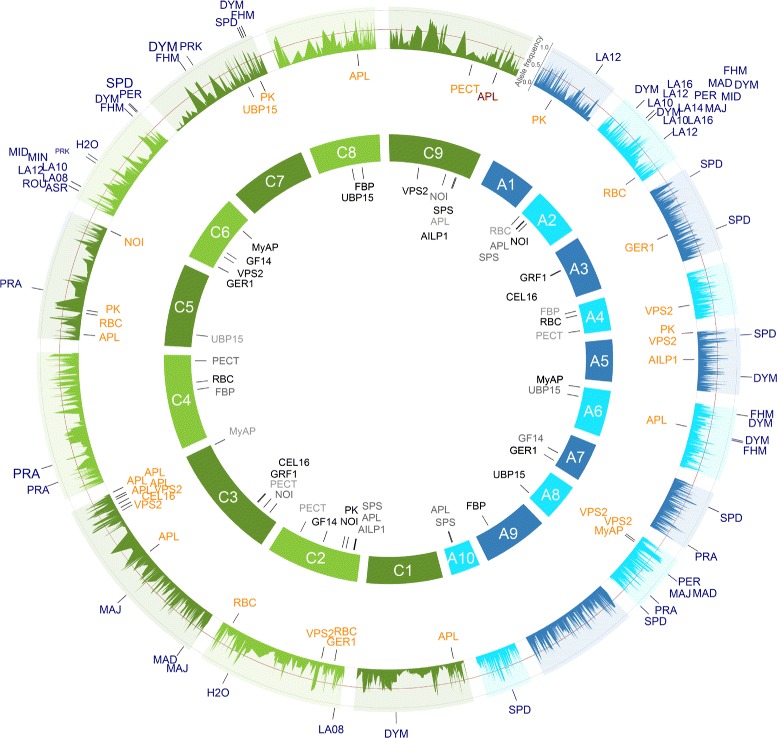
GWAS: SNP-trait/-gene associations of the inbreds of the MCLUST groups 3 of the *B. napus* diversity set. All single nucleotide polymorphism (SNP)-trait/-gene associations with a *P*-value < 0.0001 identified across the inbreds of the MCLUST groups 3 and their respective positions are marked on the *B. napus* genome. The 3,828 SNPs with their frequencies of the allele being the minor allele in the 509 inbreds are given in the outer circle. The SNPs associated with the candidate gene expression based on the gene expression data are plotted in orange below the allele frequency circle and the seedling development SNP-trait associations in blue outside the allele frequency circle. The size of the letters is related to the proportion of the variance explained by the associations. In the inner circle of the 19 chromosomes, the candidate genes were plotted to their mapping position on the *B. rapa* and *B. oleracea* reference genomes. Potential *cis*-regulated candidate genes were colored red. The A genome is colored blue and the C genome green.

For the seedling development traits projected leaf area LA08, LA10, LA12, LA14, and LA16, we identified five different hot spots (defined as associated SNPs for multiple traits) on the chromosomes A2, A7, C3, C6, and C7 for all inbreds and/or the MCLUST groups 1 to 3 (Figures 5, 6, 7 and 8). They explained in a simultaneous fit between 4.3 and 39.9% of the phenotypic variance (Table 3 and Additional file 1: Table S1, Additional file 2: Table S2, Additional file 3: Table S3). Basunanda et al. [11] found in sets of *B. napus* backcrossed test hybrids a QTL for leaf area of 28 days old seedlings in the middle of chromosome A5 at 53.5 cM which explained 3.0% of the phenotypic variance. Furthermore, Edwards and Weinig [29] measured the leaf area of one young, fully expanded leaf at bolting of 150 *B. rapa* recombinant inbred lines (RILs) across simulated seasonal settings and detected at cool temperature and short photoperiod conditions a QTL in the middle of chromosome A6 at 58.63 cM which explained 7.8% of the phenotypic variance. These discrepancies in the different studies can be explained by dissimilarities in the power to detect QTLs as well as genotype x environment and QTL x environment interactions by examining different genetic material. All three factors have the potential to lead to different QTLs in different studies [30].

The leaf area association hot spots for MCLUST group 2 and 3 on chromosome C6 were separated by ∼ 750 kb. As the average range of LD in the examined diversity set was with 677 kb close to the separation on the physical map of ∼ 750 kb, no differentiation between linkage or pleiotropy was possible in our study.

We identified association hot spots for leaf area on the bottom of chromosome A7 (MCLUST 2) and on the top of chromosome C6 (MCLUST 2 and 3) (Figures 6, 7, 8 and Additional file 7: Figure S15d-g - 19d-g). Furthermore, BLAST searches revealed that the two candidate genes *GER1* and *GF14* are located up- and downstream of these two hot spots, respectively. Thus, these two genome regions with its flanking candidate genes might be homologous regions. In contrast, the candidate gene sequence of *MyAP* was mapped by a BLAST search on the top of chromosome A6 and on the bottom of chromosome C6 (Figures 5, 6, 7 and 8). Lydiate et al. [31] and Parkin et al. [32] identified with 399 restriction fragment length polymorphism (RFLP) markers homologous genome regions between the top of chromosome A6 and the top of chromosome C6 in reverse direction as well as between the bottom of chromosome A7 and the bottom of chromosome C6. However, our result implies that these homologous genome regions might be interchanged and that the genome region on the top of chromosome A6 is homologous to the genome region on the bottom of chromosome C6 and that the genome region on the bottom of chromosome A7 is homologous to the genome region on the top of chromosome C6. This finding is in good accordance to the results of Parkin et al. [33] who analysed genome duplications within the *B. napus* genome with 455 RFLP markers and reported translocated regions which are inverted between A6 and C7 and between A6 and C6.

The MRD between the MCLUST groups 1 to 3 versus the other two MCLUST groups were, on average, 0.32, 0.34, and 0.28, respectively. Furthermore, the phenotypic variation of the examined seedling development traits which was explained by population structure, was on average 30.9% (Table 1). For some traits, the correspondence of the detected associations was low between the three subgroups. The reason for this observation can be a different genetic architecture in the three subgroups. Furthermore, different allele frequencies at the corresponding SNPs in the three different groups and the resulting differences in power to detect the associations can be the reason. It is impossible to decide based on association mapping results on one of the two reasons. This would require further analyses examining a set of bi-parental populations. Nevertheless, in this study we present in addition to the results of the subgroups also the results across all 509 inbreds to benefit from the higher power to detect SNP-trait/-gene associations.

### Variation of gene expression in seedling leaves

In the framework of our study, it was not possible to perform a genome-wide gene expression study with the available budget. Therefore, we selected seven candidate genes from main metabolic pathways to examine their correlation with seedling development traits. Furthermore, the top hub genes from a WGCNA were studied because of their potential role as high level regulators (Figure 2).

We observed a high expression of *RBC* in the seedling leaves of all 509 *B. napus* inbreds (Figure 3). This could be explained by the fact that RuBisCO (*RBC*) is the most abundant protein in plants [34]. The low expression levels of *APL* which is the predominant large subunit isoform in leaves [35] (Figure 3) were due to the fact that *APL* (E.C. 2.7.7.27) is involved in starch synthesis [36], and starch in exporting leaves represents only a transient store [37].

The expression levels of the 15 candidate genes examined in the 509 *B. napus* inbreds showed an averaged standard deviation (SD) of 5.6 across all inbreds and ranged from 3.2 to 8.8. For 16 *A. thaliana* samples, the SDs for more than 24,000 genes mostly varied between 0.5 and 5 [38]. The considerably higher SD in our study compared to that of Hruz et al. [38] might be due to the fact that our examined candidate genes were selected based on the expected different expression levels in *B. napus* seedlings. Our observation suggested that the measured gene expression differences between the 509 *B. napus* inbreds were more than adequate for the correlation with phenotypic variation of seedling development.

The candidate genes *GER1*, *AILP1*, *PECT*, and *FBP* had the highest correlations with the seedling development traits. *GER1* might play a role in plant defense, *AILP1* is involved in the response to the stimulus of auxin and aluminum ion, *PECT* is part of the phospholipid synthesis, and *FBP* is an enzyme that converts fructose-1,6-bisphosphate to fructose 6-phosphate in gluconeogenesis and the Calvin cycle and many other metabolic pathways. Thus, these genes have an essential effect on the development of rapeseed seedlings and could have great potential for breeding rapeseed varieties with improved seedling development. Therefore, not only markers associated with seedling development traits could be used for marker-assisted selection in *B. napus* to improve seedling development but also the expression of genes correlated with seedling development.

### Genome-wide associations mapping of gene expression correlated with seedling development

We mapped the gene expression levels of the 15 candidate genes in our diversity set to identify genome regions contributing to their regulation. These regions could comprise genes or specific regulators of genes influencing seedling development and might be useful for marker-assisted selection in *B. napus* to improve the seedling development.

We found across all 509 *B. napus* inbreds 31 SNP-gene associations for 13 of the 15 candidate genes with a *P*-value < 0.0001. These SNPs associated with the expression of the candidate genes explained in a simultaneous fit on average 6.9% of the phenotypic variance for a single gene (Table 3). This is in accordance with the findings of the seedling development traits which explained in a simultaneous fit, on average, 8.5% of the phenotypic variance for a single trait (Table 3). From this it follows that the expression levels of the candidate genes and the seedling development traits have a similar genetic complexity.

The SNP-gene association of *PECT* on chromosome C6 for the MCLUST group 2 is identical with the association of the projected leaf area hot spot of the seedling development traits. Mizoi et al. [39] observed that *pect1-4/pect1-6* F1 *Arabidopsis* mutants displayed severe dwarfism. *PECT* is involved as the rate-limiting step in the Kennedy pathway (phospholipid synthesis) [40] and plays a major role in the structure and function of membranes [41]. Thus, the SNP-*PECT* association on chromosome C6 may caused the differences in leaf area growth of the examined *B. napus* seedlings.

The genome positions of the SNP-gene associations of *RBC* on the chromosomes A4 and C4 and *FBP* on the chromosomes A9 and C8 were in accordance with their genome position (Figures 5, 6, 7 and 8, red colored SNP-gene associations). According to Chen et al. [42] these associations were defined as *cis*-regulated, because the SNP-gene associations were within 677 kb upstream or downstream of this gene position mapped by a BLAST search. In the neighborhood of the SNP-gene association of the candidate gene *RBC* on chromosome A4 at 6,466,112 bp the *B. napus* genes Bra028181, Bra028174, and Bra028175 were located. Their best BLASTX hits to *A. thaliana* are the genes AT5G38430 and AT5G38420 which are encoding the RuBisCO small subunit 2B or 1B of *A. thaliana*, respectively. Furthermore, the SNP-gene association of *FBP* on chromosome A9 at 27,053,307 bp is nearby the *B. napus* gene Bra007041 of which the best hit by BLASTX to *A. thaliana* is the gene AT3G54050 encoding for fructose 1,6-bisphosphate phosphatase. All the other SNP-gene associations were outside this range and therefore defined as *trans*-regulated (Figures 5, 6, 7 and 8). Thus, these *trans*-regulatory SNP-gene associations most likely encode transcriptional regulators which requires further research.

## Conclusions

In this paper we conducted the largest genome-wide association study on seedling development traits in *Brassica napus* using a diversity set comprising 509 inbreds. A total of 99.4% of the adjacent SNPs on the A genome and 93.0% of the adjacent SNPs on the C genome had a distance smaller than the average range of LD. Therefore, this genome-wide association study is expected to result on average in 14.7% of the possible power. Compared to previous studies in *B. napus*, the SNP marker density of our study is expected to provide a higher power to detect SNP-trait/-gene associations in the *B. napus* diversity set. The large number of associations detected for the examined 14 seedling development traits indicated that these are genetically complex inherited. Based on a weighted gene co-expression network analysis in a segregating population, regulatory genes were selected to analyse their gene expression in seedling leaves in the diversity set. The candidate genes *GER1*, *AILP1*, *PECT*, and *FBP* were strongly correlated with the seedling development traits. Thus, these genes might be interesting targets for breeding and have potential for breeding rapeseed varieties with improved seedling development. For the projected leaf area traits, we identified five different association hot spots on the chromosomes A2, A7, C3, C6, and C7. Further research is required to identify the causative polymorphisms in these association hot spots.

## Availability of supporting data

The data sets supporting the results of this article are included within the article and its additional files (Additional file 4: Table S4, Additional file 5: Table S5).

## Additional files

Additional file 1
**Table S1.** SNP-associations MCLUST 1. SNP associations of MCLUST group 1. Single nucleotide polymorphism (SNP)-trait/-gene associations with P < 0.0001 across the inbreds of MCLUST group 1.

Additional file 2
**Table S2.** SNP-associations MCLUST 2. SNP associations of MCLUST group 2. Single nucleotide polymorphism (SNP)-trait/-gene associations with P < 0.0001 across the inbreds of MCLUST group 2.

Additional file 3
**Table S3.** SNP-associations MCLUST 3. SNP associations of MCLUST group 3. Single nucleotide polymorphism (SNP)-trait/-gene associations with P < 0.0001 across the inbreds of MCLUST group 3.

Additional file 4
**Table S4.** Phenotypic data. Mean values of the phenotypic data of seedling development and gene expression of the 509 inbreds of the ASSYST diversity set of *B. napus*.

Additional file 5
**Table S5.** SNP data. Single nucleotide polymorphism (SNP) data of the 509 inbreds of the ASSYST diversity set of *B. napus*.

Additional file 6
**Figure S1-S14.** Distribution of the expression levels of the candidate genes and their *P*-value profile from genome-wide association mapping (GWAS).

Additional file 7
**Figure S15-S34.** Distribution of the 20 seedling development traits and their *P*-value profile from genome-wide association mapping (GWAS).

Additional file 8
**Figure S35-S38.** Correlations of the seedling development traits and the gene expression levels across all 509 inbreds as well as the inbreds of the MCLUST groups 1 to 3.

## Abbreviations

QTL: Quantitative trait loci
LD: Linkage disequilibrium
SSR: Simple sequence repeat
AFLP: Amplified fragment-length polymorphism
SNP: Single nucleotide polymorphism
GWAS: Genome-wide association study
OSR: Oilseed rape
DH: Doubled haploid
WGCNA: Weighted gene co-expression network analysis
RNA: Ribonucleic acid
mRNA: Messenger RNA
DGE-seq: Digital gene expression sequencing
RT-qPCR: Reverse transcription quantitative polymerase chain reaction
DNA: Deoxyribonucleic acid
cDNA: Complementary DNA
BLAST: Basic local alignment search tool
PCA: Principal component analysis
MRD: Modified Roger’s distance
RIL: Recombinant inbred line
RFLP: Restriction fragment length polymorphism
SD: Standard deviation
