# Supplementary material for: Seedling development traits in Brassicanapus examined by gene expression analysis and association mapping
Source: BMC Plant Biol. 2015 Jun 9;15:136. doi: 10.1186/s12870-015-0496-3 (PMC4459455; doi:10.1186/s12870-015-0496-3)
Supplement: Additional file 6 — Figure S1-S14. Distribution of the expression levels of the candidate genes and their P-value profile from genome-wide association mapping (GWAS). [file 12870_2015_496_MOESM6_ESM.pdf]

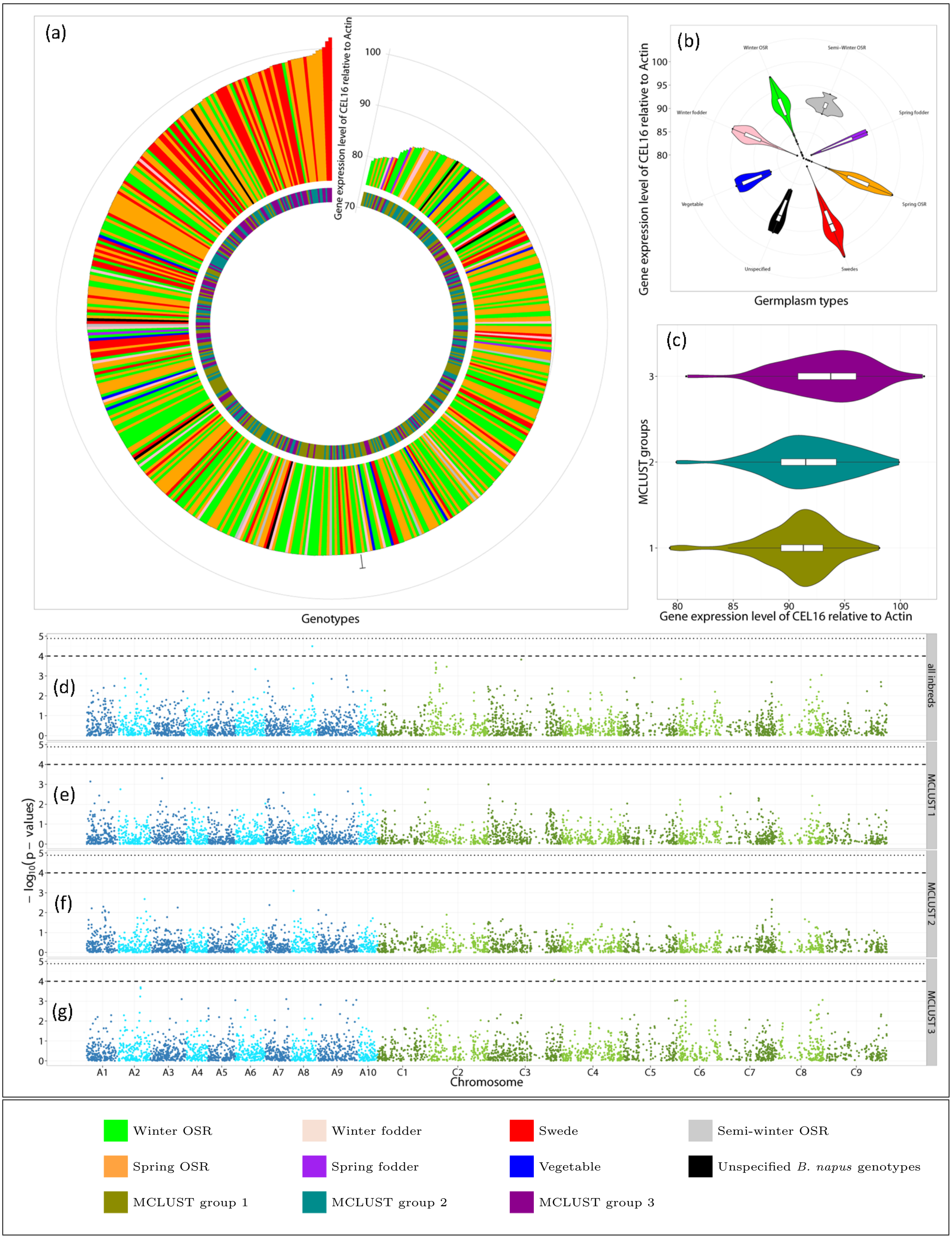

Figure S1. (a) Distribution of the expression level of the gene *CEL16* relative to the housekeeping gene *Actin* across all 509 inbreds ordered by the gene expression level. (b) Violinplot of the gene expression level of *CEL16* for the eight different germplasm types and (c) for the three MCLUST groups. (d) P-value profile from genome-wide association mapping for the gene expression level of the *CEL16* gene for all 509 inbreds, (e) for the inbreds of the MCLUST group 1, (f) for the inbreds of the MCLUST group 2, and (g) for the inbreds of the MCLUST group 3. The x-axis shows physical map positions of the SNPs along the 19 chromosomes, the y-axis gives the -$\log_{10}$ P-value of the association test. The horizontal dashed and dotted lines indicate the $\alpha = 0.0001$ threshold and the threshold after Bonferroni correction (α=0.05), respectively.

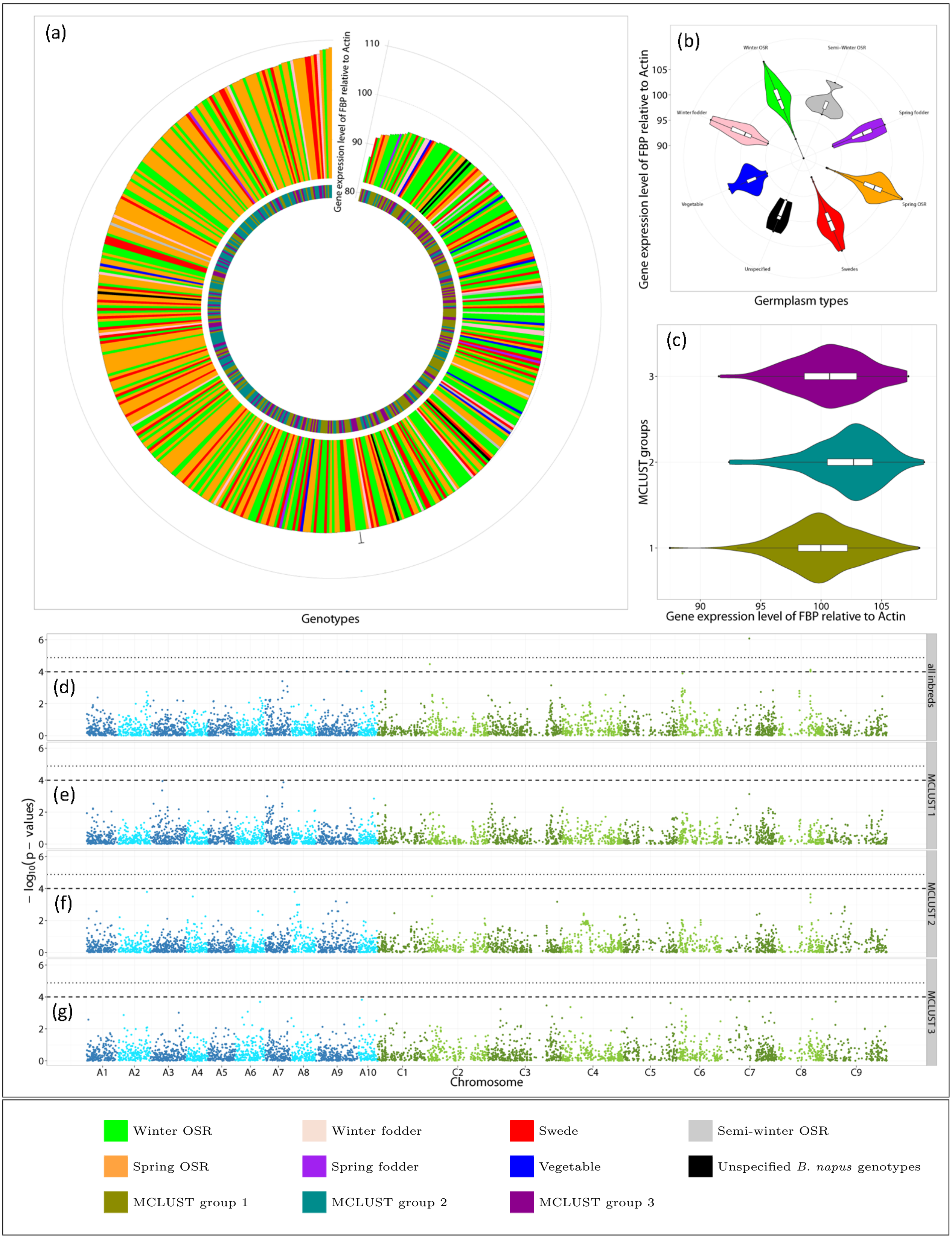

Figure S2. (a) Distribution of the expression level of the gene *FBP* relative to the housekeeping gene *Actin* across all 509 inbreds ordered by the gene expression level. (b) Violinplot of the gene expression level of *FBP* for the eight different germplasm types and (c) for the three MCLUST groups. (d) *P*-value profile from genome-wide association mapping for the gene expression level of the *FBP* gene for all 509 inbreds, (e) for the inbreds of the MCLUST group 1, (f) for the inbreds of the MCLUST group 2, and (g) for the inbreds of the MCLUST group 3. The x-axis shows physical map positions of the SNPs along the 19 chromosomes, the y-axis gives the -$\log_{10}$ *P*-value of the association test. The horizontal dashed and dotted lines indicate the $\alpha = 0.0001$ threshold and the threshold after Bonferroni correction (α=0.05), respectively.

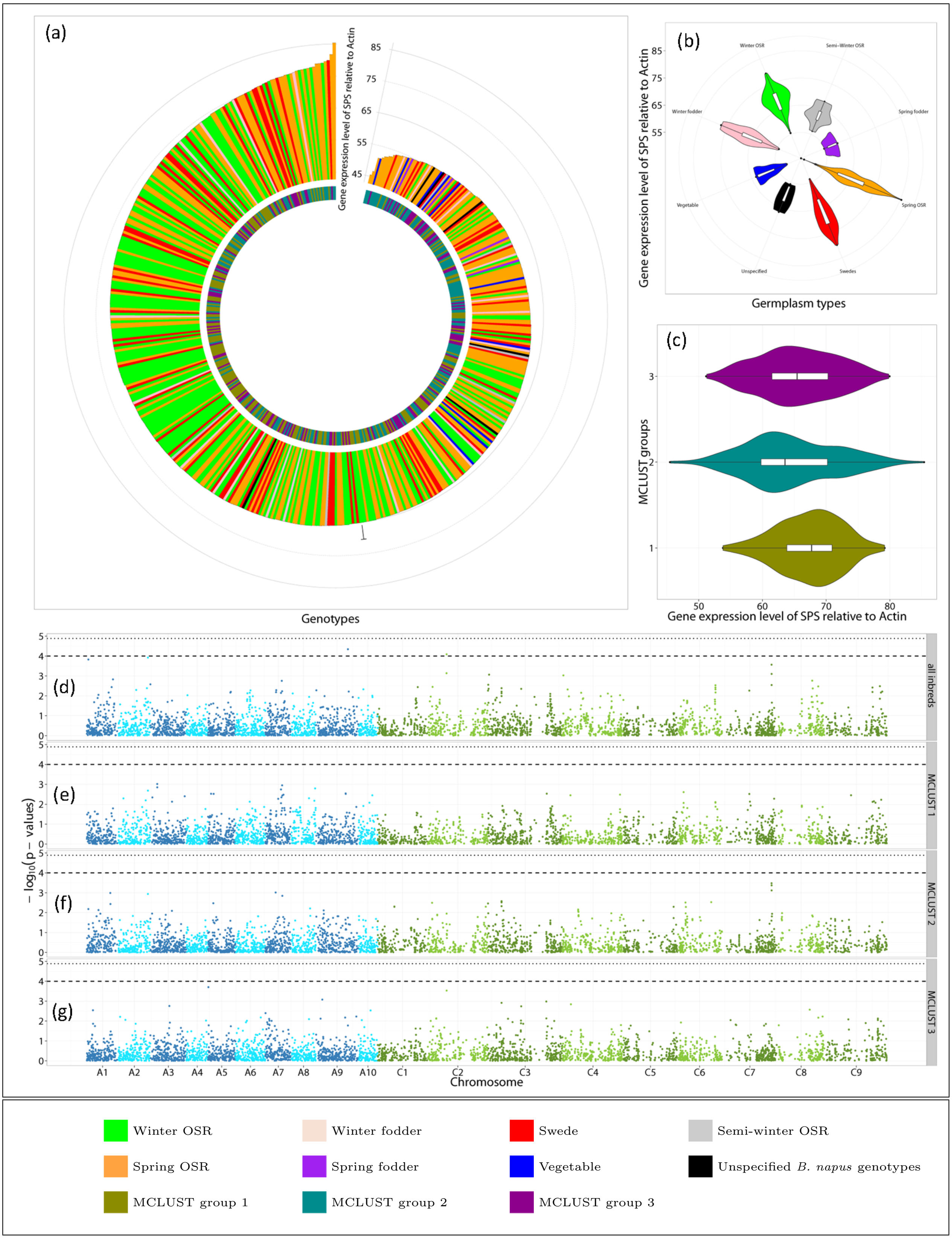

Figure S3. (a) Distribution of the expression level of the gene *SPS* relative to the housekeeping gene *Actin* across all 509 inbreds ordered by the gene expression level. (b) Violinplot of the gene expression level of *SPS* for the eight different germplasm types and (c) for the three MCLUST groups. (d) *P*-value profile from genome-wide association mapping for the gene expression level of the *SPS* gene for all 509 inbreds, (e) for the inbreds of the MCLUST group 1, (f) for the inbreds of the MCLUST group 2, and (g) for the inbreds of the MCLUST group 3. The x-axis shows physical map positions of the SNPs along the 19 chromosomes, the y-axis gives the -$\log_{10}$ *P*-value of the association test. The horizontal dashed and dotted lines indicate the $\alpha = 0.0001$ threshold and the threshold after Bonferroni correction (α=0.05), respectively.

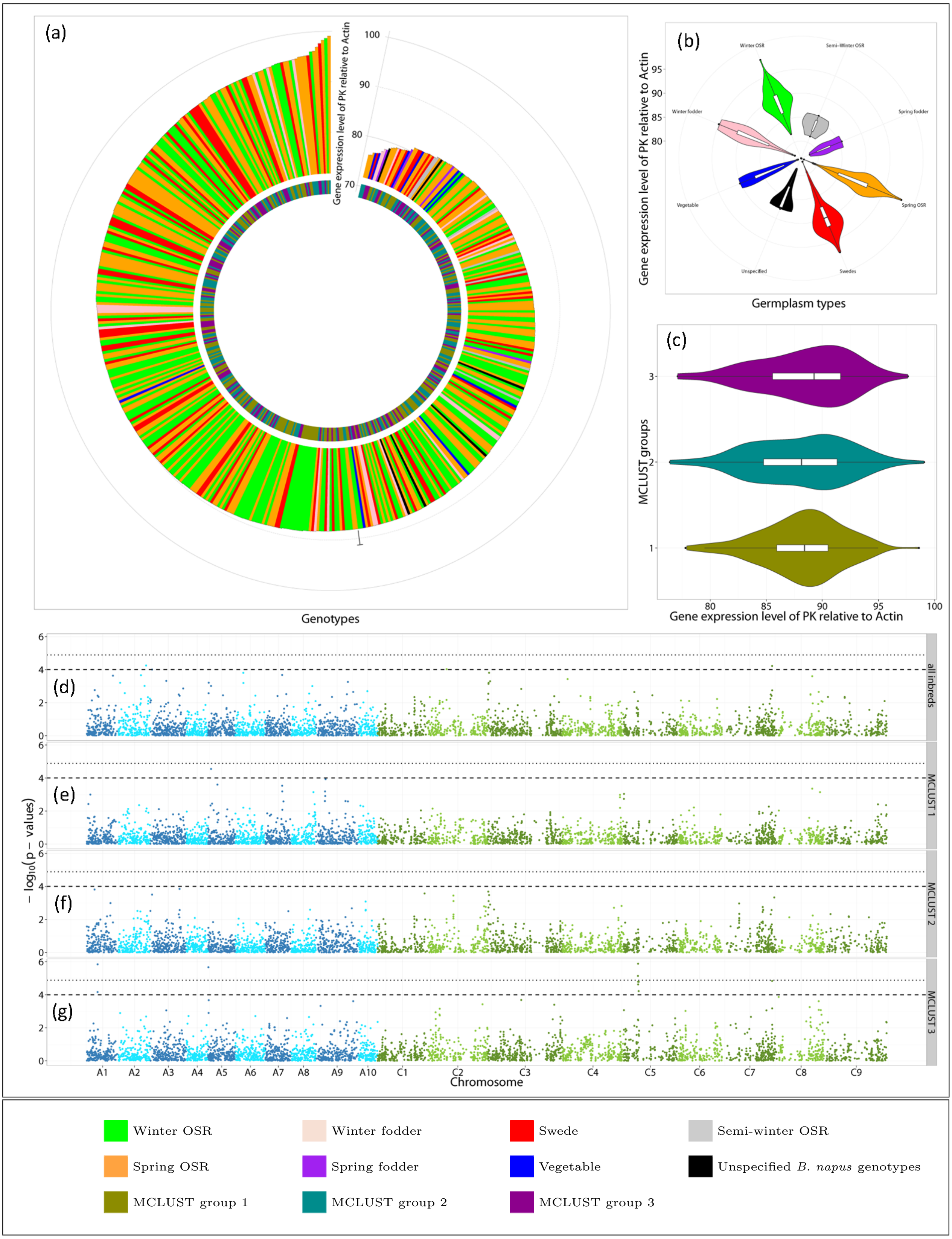

Figure S4. (a) Distribution of the expression level of the gene *PK* relative to the housekeeping gene *Actin* across all 509 inbreds ordered by the gene expression level. (b) Violinplot of the gene expression level of *PK* for the eight different germplasm types and (c) for the three MCLUST groups. (d) *P*-value profile from genome-wide association mapping for the gene expression level of the *PK* gene for all 509 inbreds, (e) for the inbreds of the MCLUST group 1, (f) for the inbreds of the MCLUST group 2, and (g) for the inbreds of the MCLUST group 3. The x-axis shows physical map positions of the SNPs along the 19 chromosomes, the y-axis gives the -$\log_{10}$ *P*-value of the association test. The horizontal dashed and dotted lines indicate the $\alpha = 0.0001$ threshold and the threshold after Bonferroni correction (α=0.05), respectively.

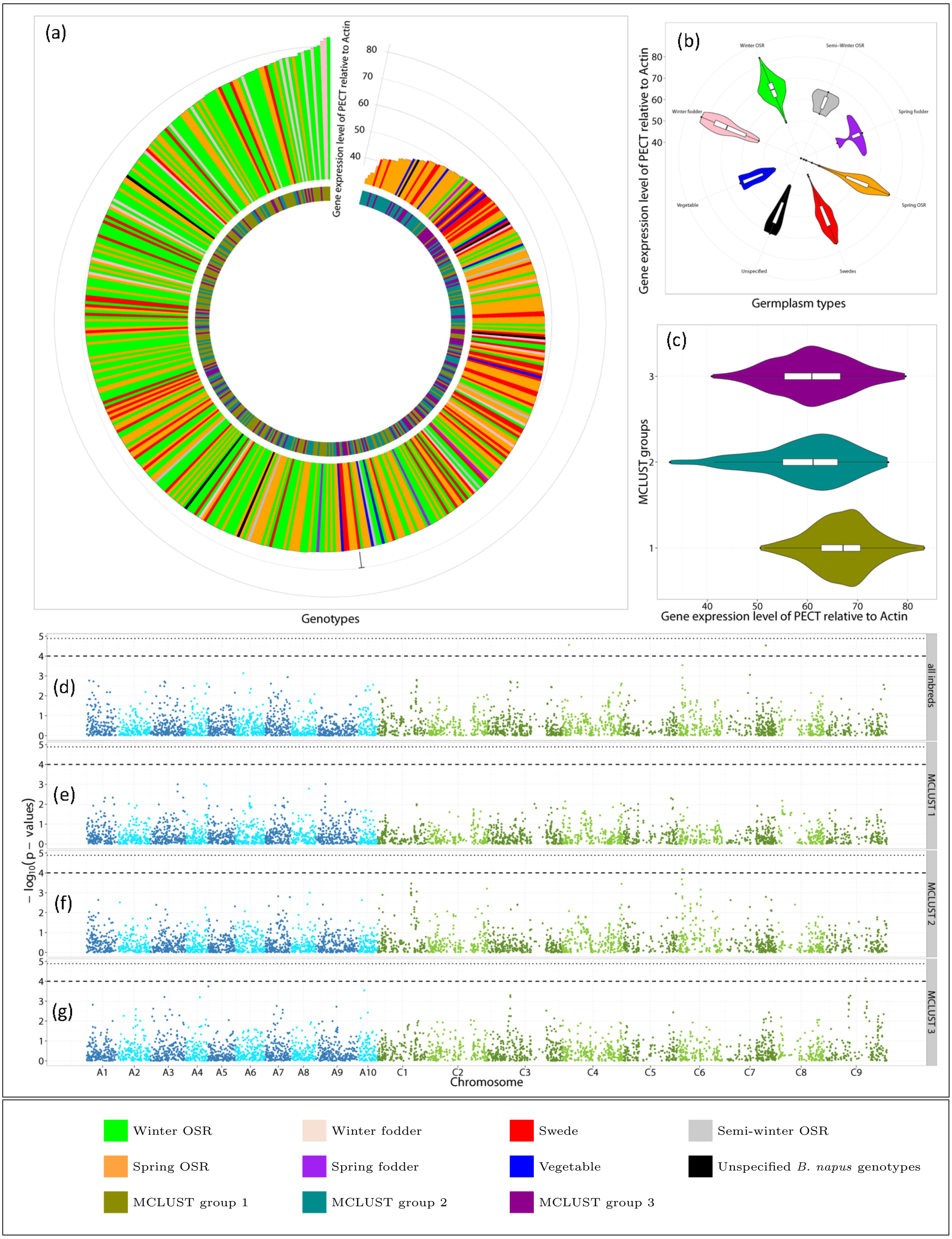

Figure S5. (a) Distribution of the expression level of the gene *PECT* relative to the housekeeping gene *Actin* across all 509 inbreds ordered by the gene expression level. (b) Violinplot of the gene expression level of *PECT* for the eight different germplasm types and (c) for the three MCLUST groups. (d) P-value profile from genome-wide association mapping for the gene expression level of the *PECT* gene for all 509 inbreds, (e) for the inbreds of the MCLUST group 1, (f) for the inbreds of the MCLUST group 2, and (g) for the inbreds of the MCLUST group 3. The x-axis shows physical map positions of the SNPs along the 19 chromosomes, the y-axis gives the -$\log_{10}$ P-value of the association test. The horizontal dashed and dotted lines indicate the $\alpha = 0.0001$ threshold and the threshold after Bonferroni correction (α=0.05), respectively.

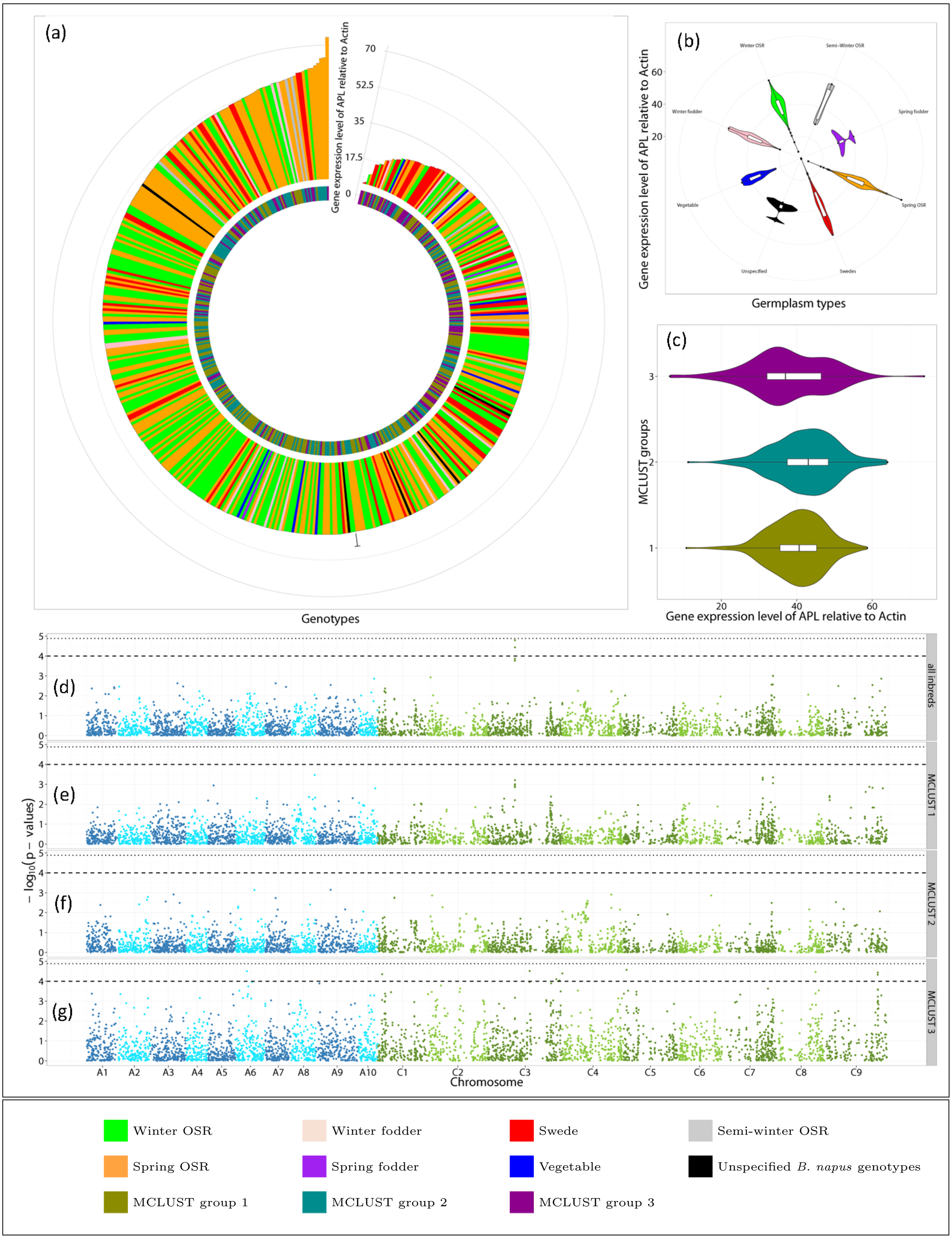

Figure S6. (a) Distribution of the expression level of the gene *APL* relative to the housekeeping gene *Actin* across all 509 inbreds ordered by the gene expression level. (b) Violinplot of the gene expression level of *APL* for the eight different germplasm types and (c) for the three MCLUST groups. (d) *P*-value profile from genome-wide association mapping for the gene expression level of the *APL* gene for all 509 inbreds, (e) for the inbreds of the MCLUST group 1, (f) for the inbreds of the MCLUST group 2, and (g) for the inbreds of the MCLUST group 3. The x-axis shows physical map positions of the SNPs along the 19 chromosomes, the y-axis gives the -$\log_{10}$ *P*-value of the association test. The horizontal dashed and dotted lines indicate the $\alpha = 0.0001$ threshold and the threshold after Bonferroni correction (α=0.05), respectively.

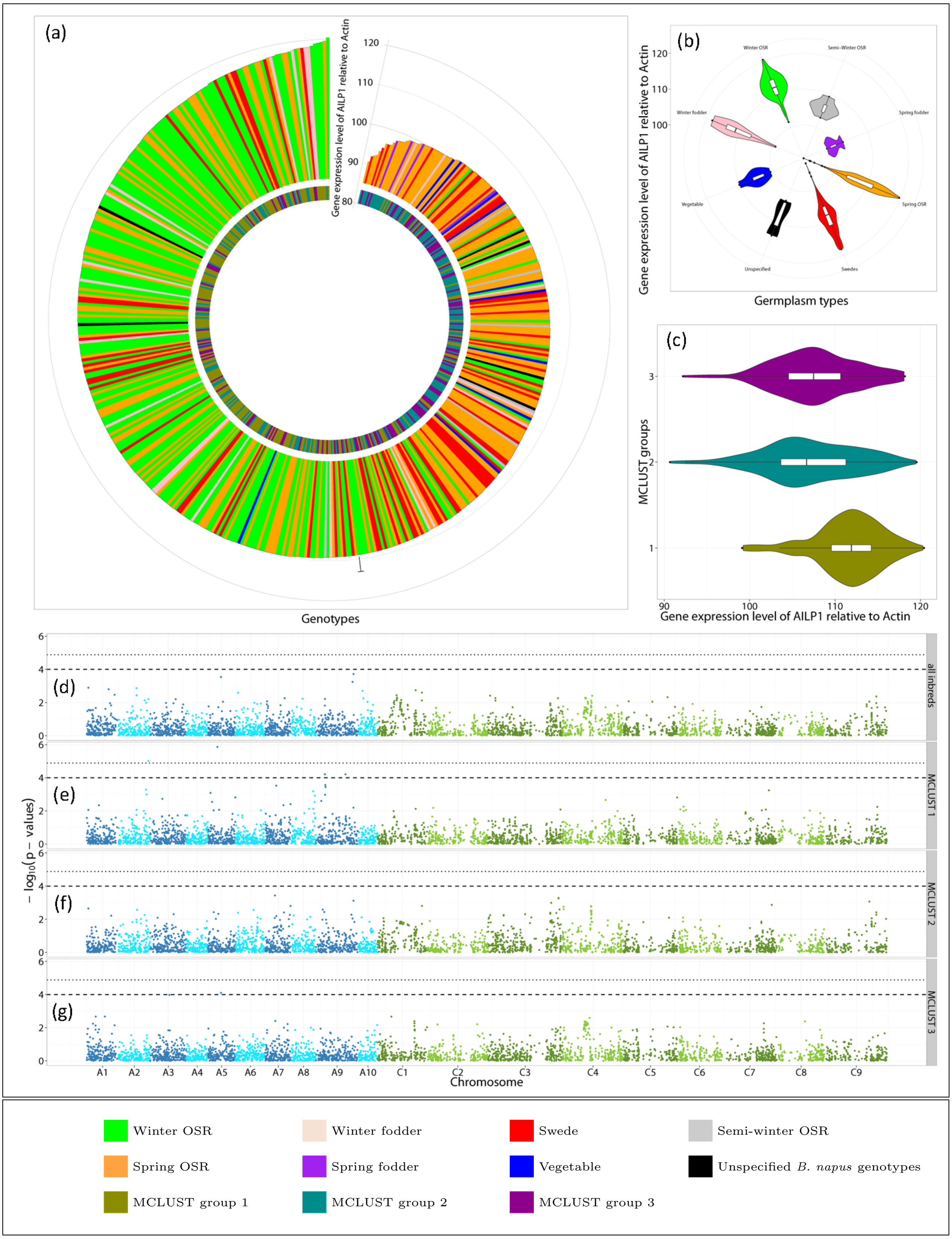

Figure S7. (a) Distribution of the expression level of the gene *AILP1* relative to the housekeeping gene *Actin* across all 509 inbreds ordered by the gene expression level. (b) Violinplot of the gene expression level of *AILP1* for the eight different germplasm types and (c) for the three MCLUST groups. (d) *P*-value profile from genome-wide association mapping for the gene expression level of the *AILP1* gene for all 509 inbreds, (e) for the inbreds of the MCLUST group 1, (f) for the inbreds of the MCLUST group 2, and (g) for the inbreds of the MCLUST group 3. The x-axis shows physical map positions of the SNPs along the 19 chromosomes, the y-axis gives the -$\log_{10}$ *P*-value of the association test. The horizontal dashed and dotted lines indicate the $\alpha = 0.0001$ threshold and the threshold after Bonferroni correction (α=0.05), respectively.

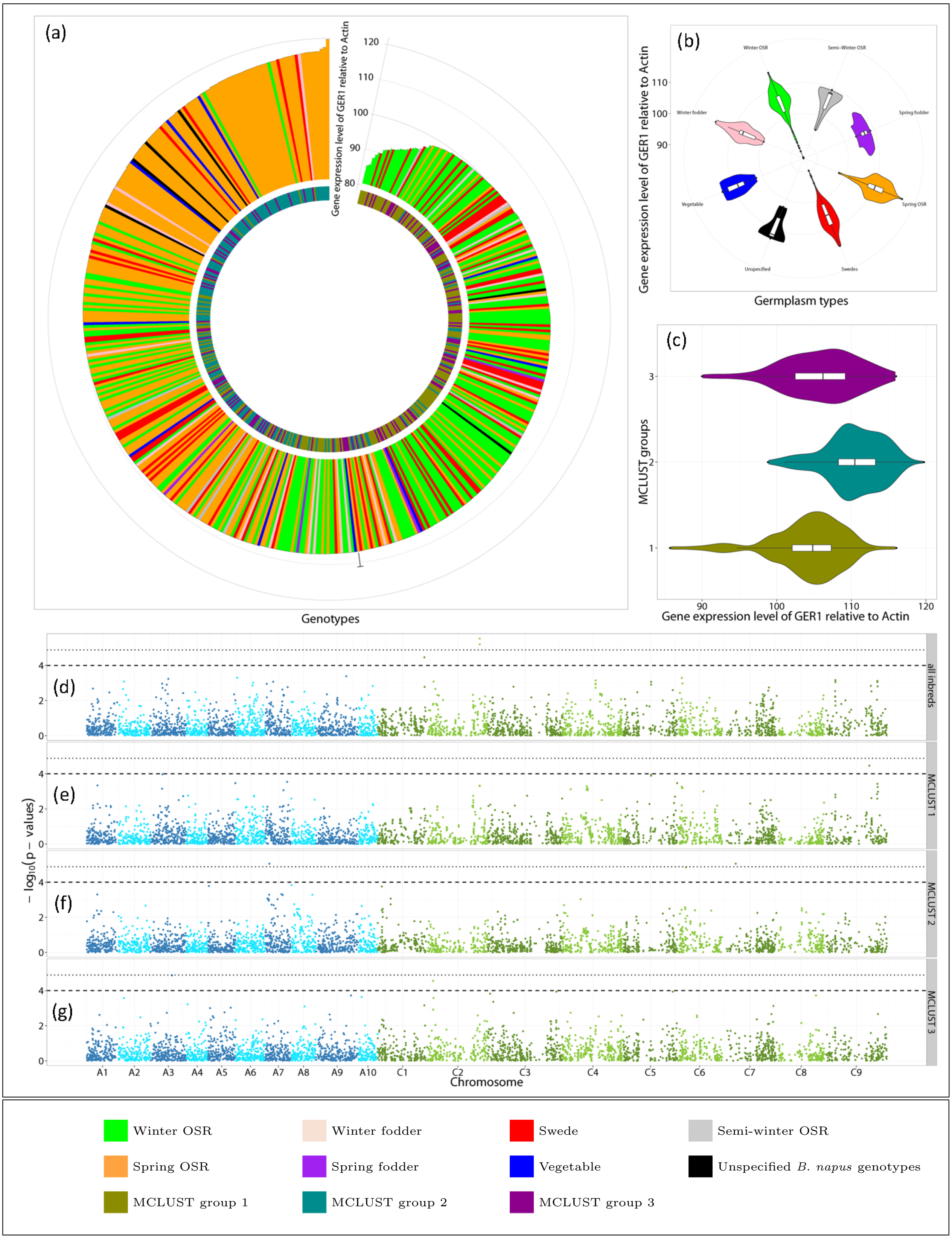

Figure S8. (a) Distribution of the expression level of the gene *GER1* relative to the housekeeping gene *Actin* across all 509 inbreds ordered by the gene expression level. (b) Violinplot of the gene expression level of *GER1* for the eight different germplasm types and (c) for the three MCLUST groups. (d) *P*-value profile from genome-wide association mapping for the gene expression level of the *GER1* gene for all 509 inbreds, (e) for the inbreds of the MCLUST group 1, (f) for the inbreds of the MCLUST group 2, and (g) for the inbreds of the MCLUST group 3. The x-axis shows physical map positions of the SNPs along the 19 chromosomes, the y-axis gives the -$\log_{10}$ *P*-value of the association test. The horizontal dashed and dotted lines indicate the $\alpha = 0.0001$ threshold and the threshold after Bonferroni correction (α=0.05), respectively.

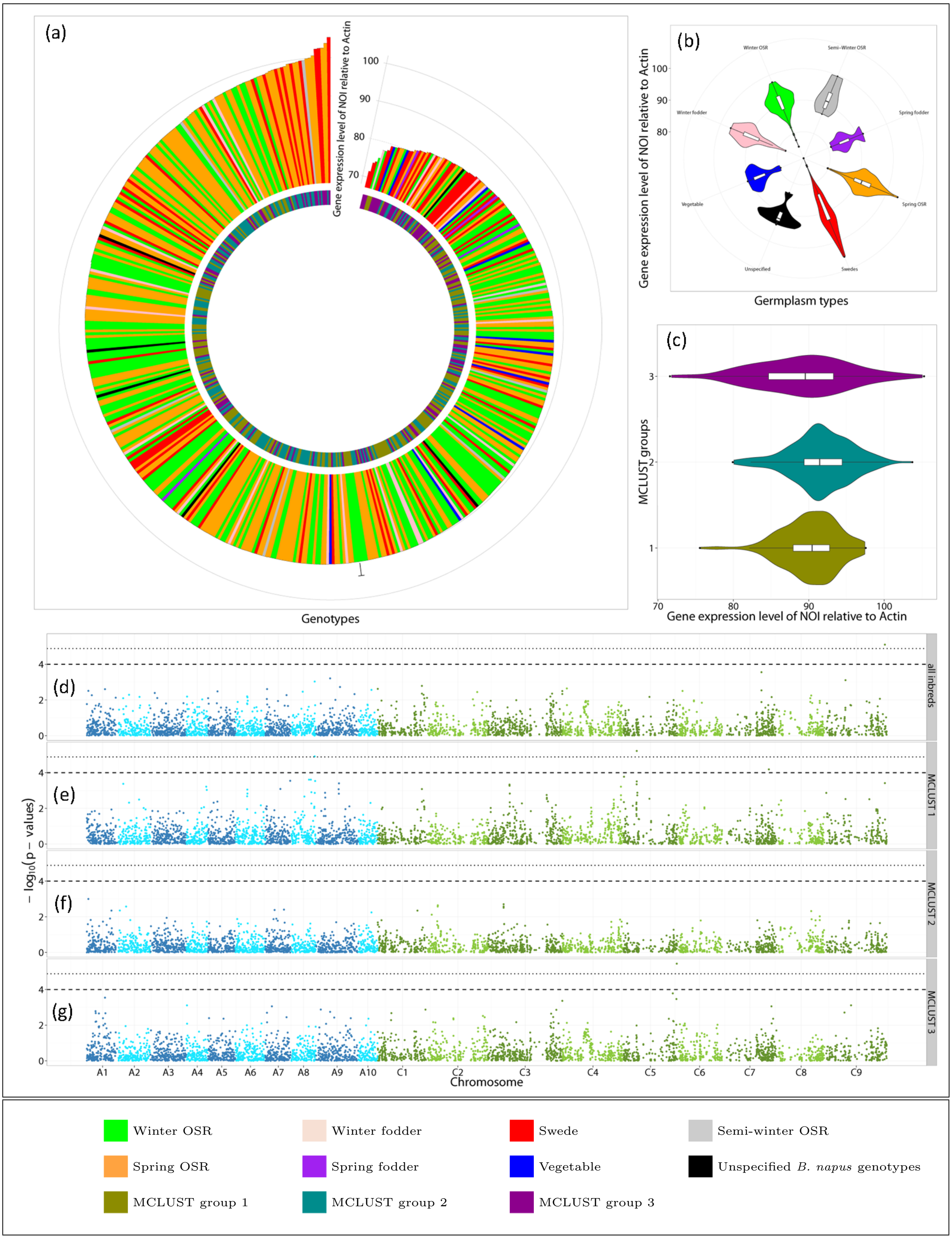

Figure S9. (a) Distribution of the expression level of the gene *NOI* relative to the housekeeping gene *Actin* across all 509 inbreds ordered by the gene expression level. (b) Violinplot of the gene expression level of *NOI* for the eight different germplasm types and (c) for the three MCLUST groups. (d) P-value profile from genome-wide association mapping for the gene expression level of the *NOI* gene for all 509 inbreds, (e) for the inbreds of the MCLUST group 1, (f) for the inbreds of the MCLUST group 2, and (g) for the inbreds of the MCLUST group 3. The x-axis shows physical map positions of the SNPs along the 19 chromosomes, the y-axis gives the -$\log_{10}$ P-value of the association test. The horizontal dashed and dotted lines indicate the $\alpha = 0.0001$ threshold and the threshold after Bonferroni correction (α=0.05), respectively.

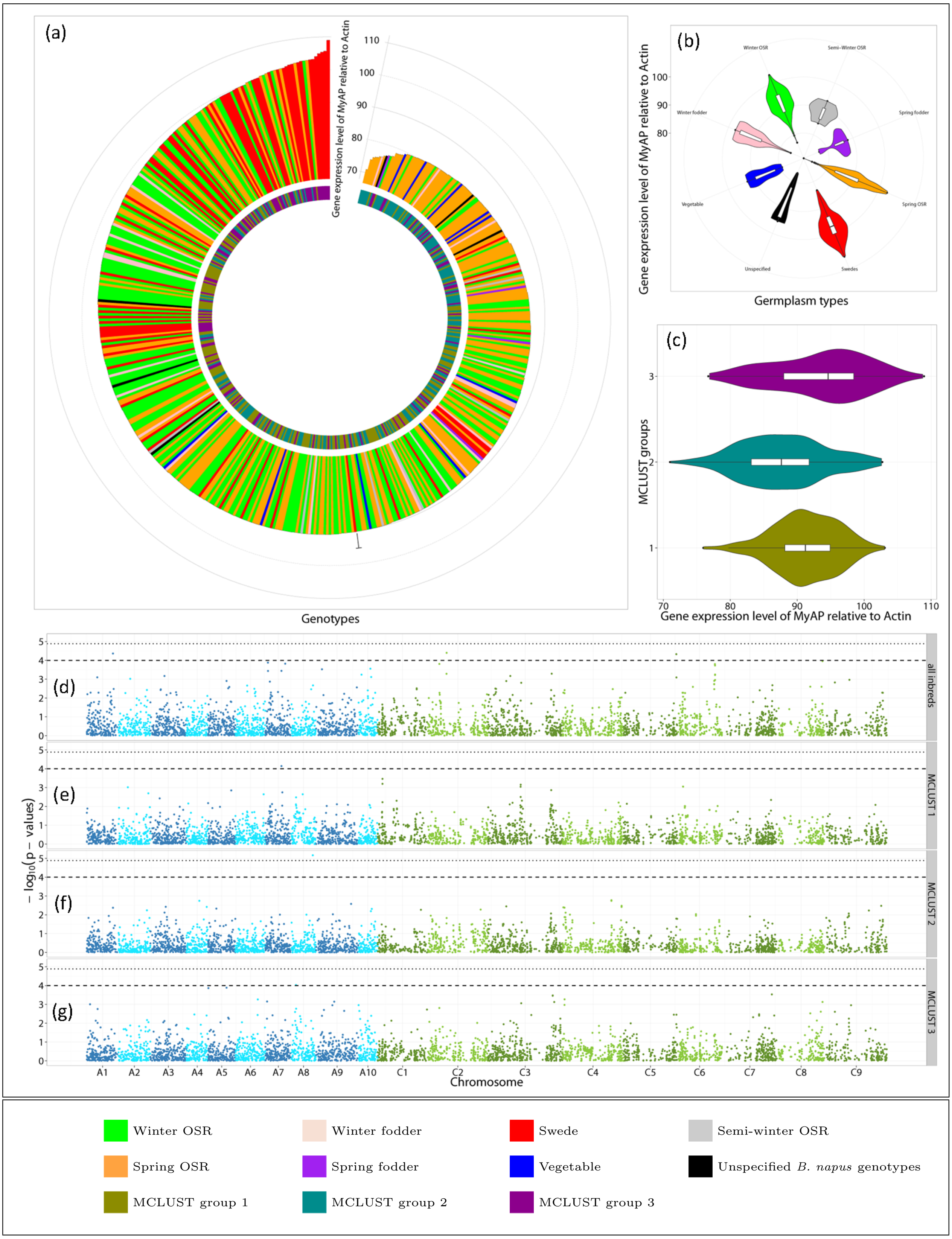

Figure S10. (a) Distribution of the expression level of the gene *MyAP* relative to the housekeeping gene *Actin* across all 509 inbreds ordered by the gene expression level. (b) Violinplot of the gene expression level of *MyAP* for the eight different germplasm types and (c) for the three MCLUST groups. (d) *P*-value profile from genome-wide association mapping for the gene expression level of the *MyAP* gene for all 509 inbreds, (e) for the inbreds of the MCLUST group 1, (f) for the inbreds of the MCLUST group 2, and (g) for the inbreds of the MCLUST group 3. The x-axis shows physical map positions of the SNPs along the 19 chromosomes, the y-axis gives the -$\log_{10}$ *P*-value of the association test. The horizontal dashed and dotted lines indicate the $\alpha = 0.0001$ threshold and the threshold after Bonferroni correction (α=0.05), respectively.

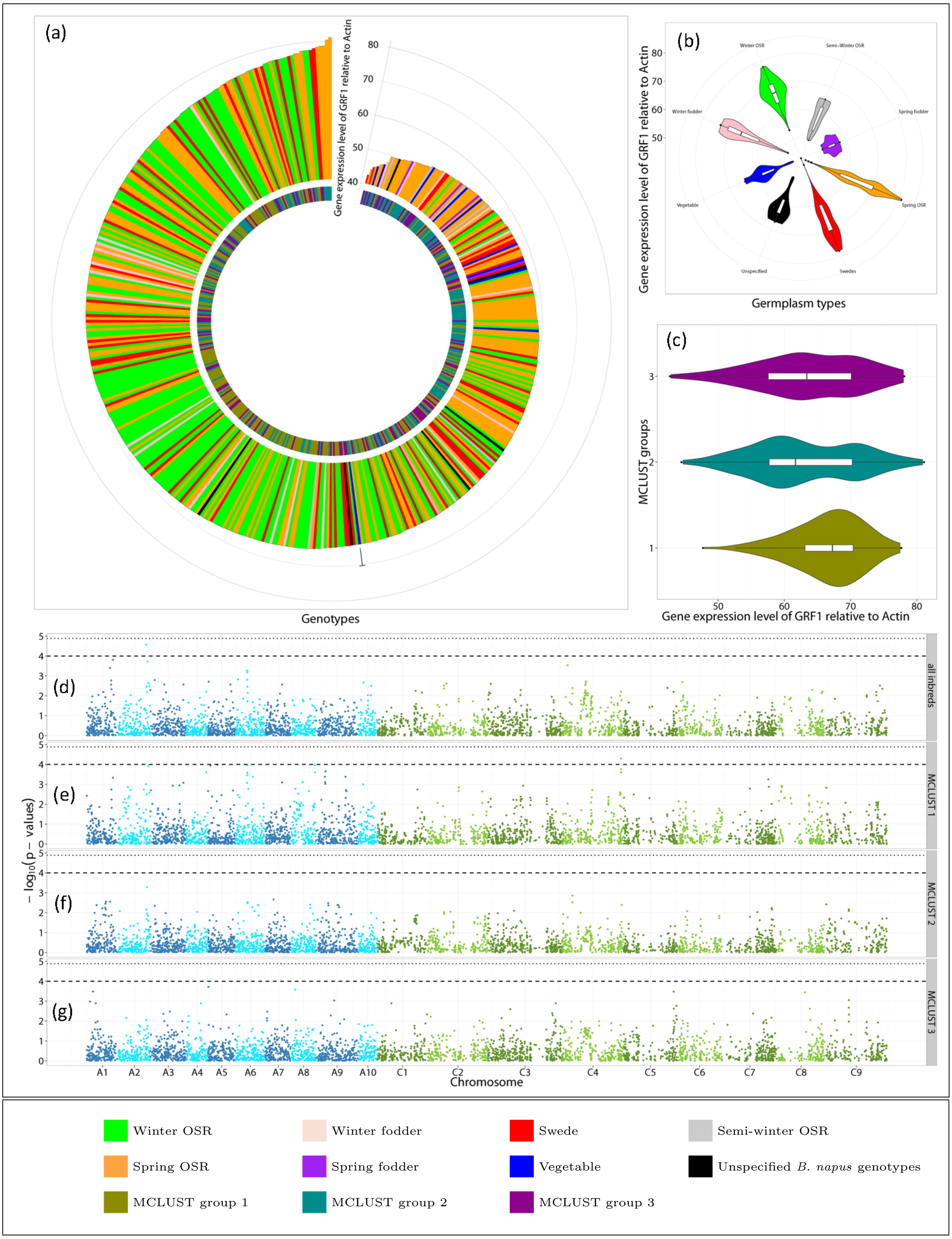

Figure S11. (a) Distribution of the expression level of the gene *GRF1* relative to the housekeeping gene *Actin* across all 509 inbreds ordered by the gene expression level. (b) Violinplot of the gene expression level of *GRF1* for the eight different germplasm types and (c) for the three MCLUST groups. (d) *P*-value profile from genome-wide association mapping for the gene expression level of the *GRF1* gene for all 509 inbreds, (e) for the inbreds of the MCLUST group 1, (f) for the inbreds of the MCLUST group 2, and (g) for the inbreds of the MCLUST group 3. The x-axis shows physical map positions of the SNPs along the 19 chromosomes, the y-axis gives the -log$_{10}$ *P*-value of the association test. The horizontal dashed and dotted lines indicate the $\alpha = 0.0001$ threshold and the threshold after Bonferroni correction (α=0.05), respectively.

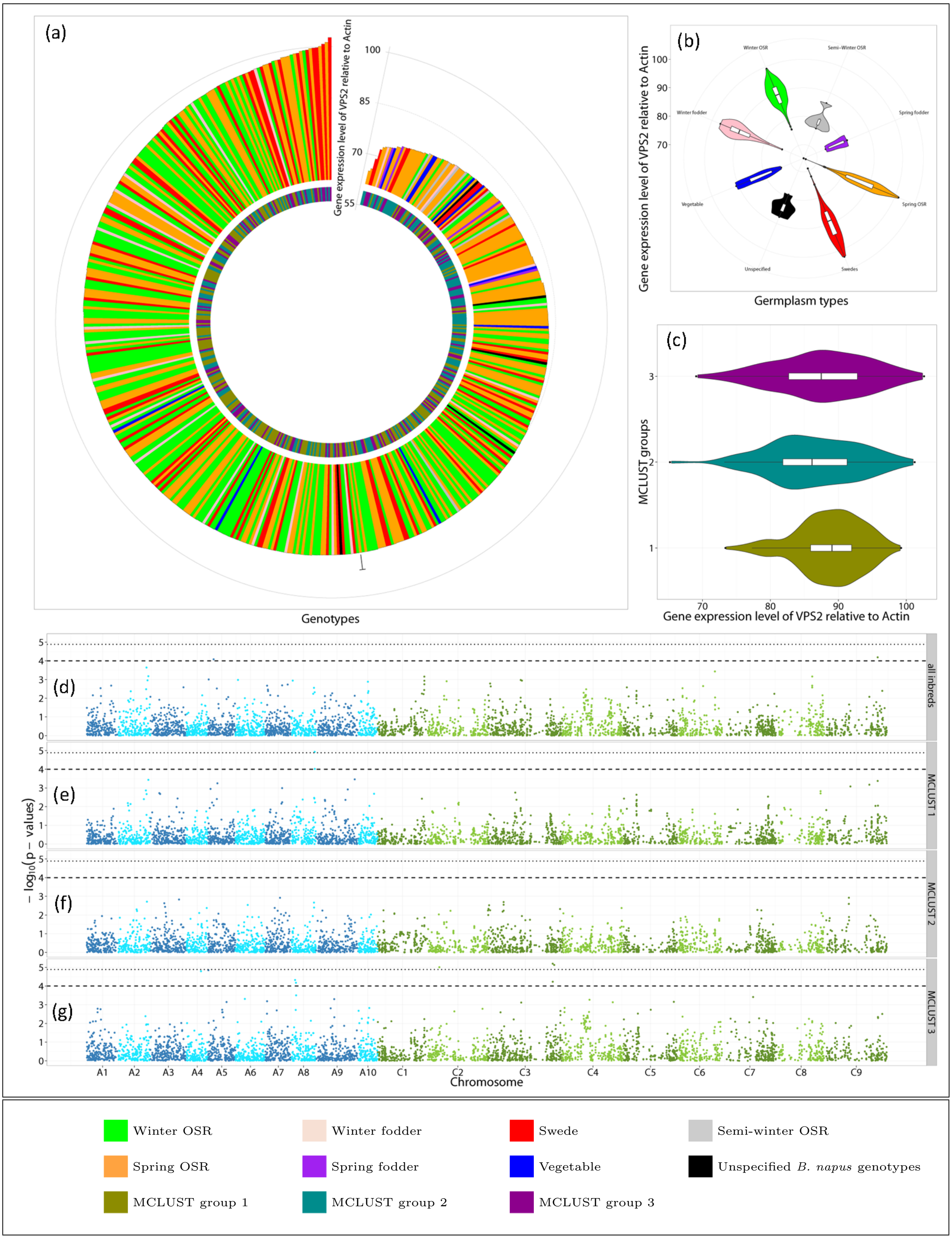

Figure S12. (a) Distribution of the expression level of the gene *VPS2* relative to the housekeeping gene *Actin* across all 509 inbreds ordered by the gene expression level. (b) Violinplot of the gene expression level of *VPS2* for the eight different germplasm types and (c) for the three MCLUST groups. (d) *P*-value profile from genome-wide association mapping for the gene expression level of the *VPS2* gene for all 509 inbreds, (e) for the inbreds of the MCLUST group 1, (f) for the inbreds of the MCLUST group 2, and (g) for the inbreds of the MCLUST group 3. The x-axis shows physical map positions of the SNPs along the 19 chromosomes, the y-axis gives the -$\log_{10}$ *P*-value of the association test. The horizontal dashed and dotted lines indicate the $\alpha = 0.0001$ threshold and the threshold after Bonferroni correction (α=0.05), respectively.

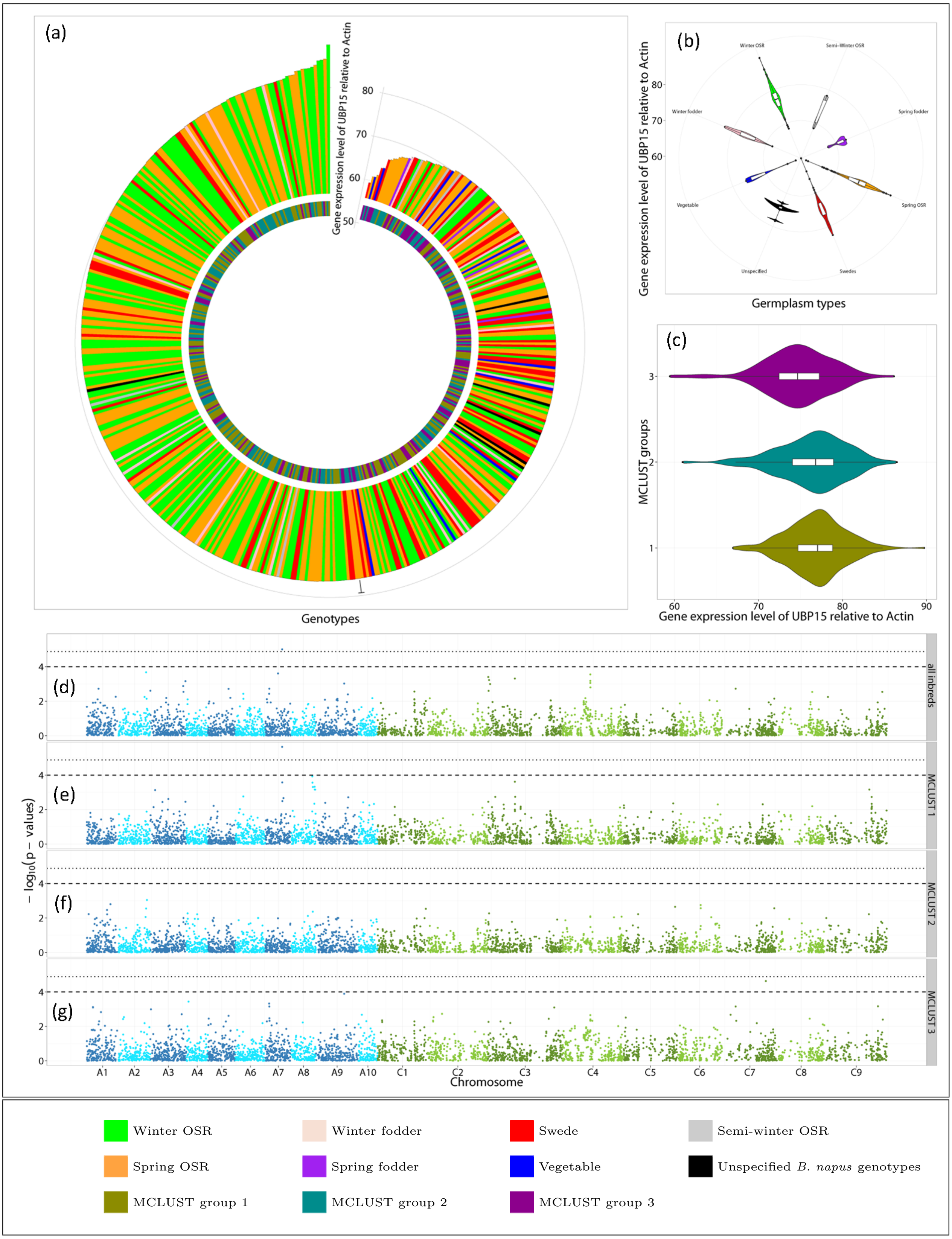

Figure S13. (a) Distribution of the expression level of the gene *UBP15* relative to the housekeeping gene *Actin* across all 509 inbreds ordered by the gene expression level. (b) Violinplot of the gene expression level of *UBP15* for the eight different germplasm types and (c) for the three MCLUST groups. (d) *P*-value profile from genome-wide association mapping for the gene expression level of the *UBP15* gene for all 509 inbreds, (e) for the inbreds of the MCLUST group 1, (f) for the inbreds of the MCLUST group 2, and (g) for the inbreds of the MCLUST group 3. The x-axis shows physical map positions of the SNPs along the 19 chromosomes, the y-axis gives the -$\log_{10}$ *P*-value of the association test. The horizontal dashed and dotted lines indicate the $\alpha = 0.0001$ threshold and the threshold after Bonferroni correction (α=0.05), respectively.

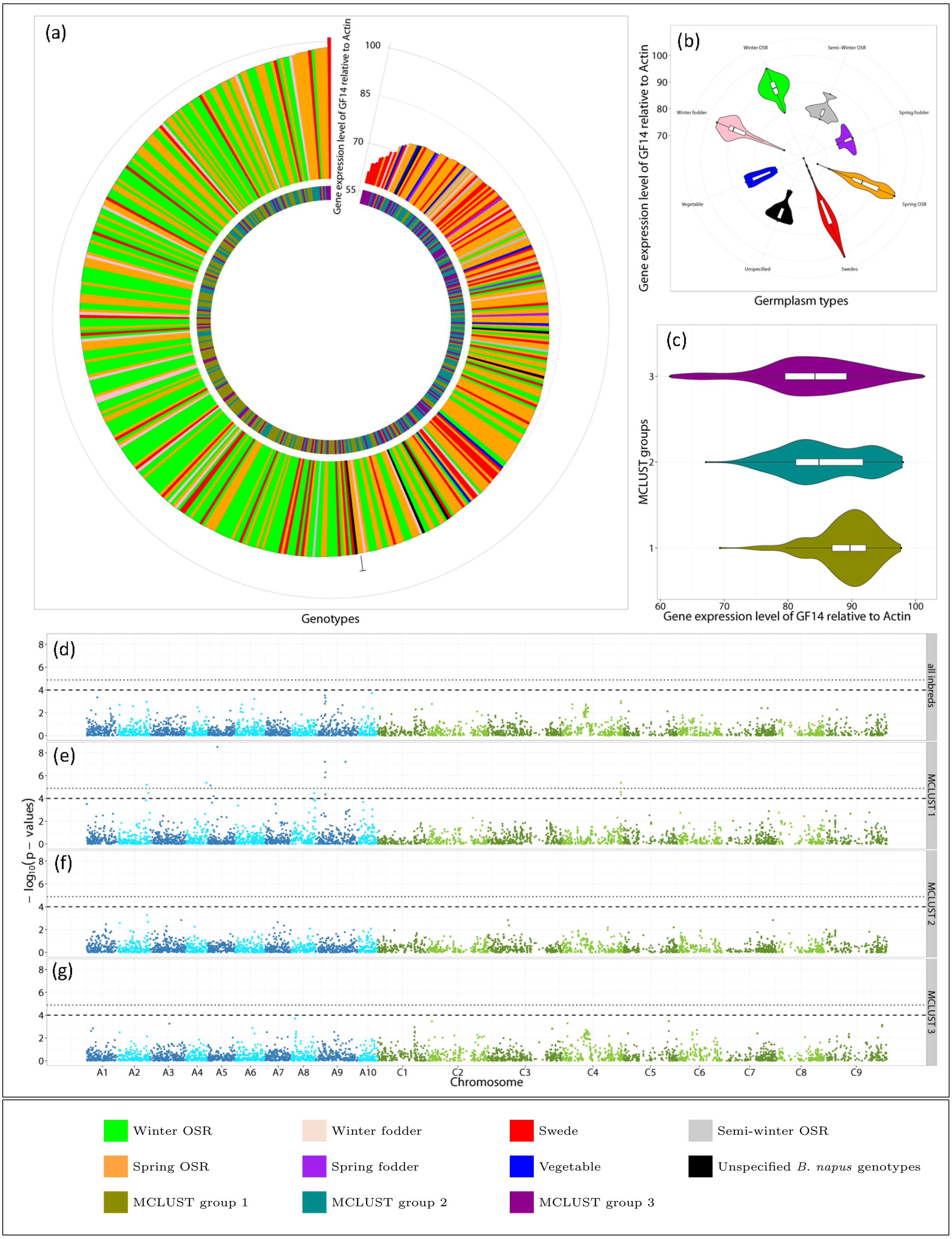

Figure S14. (a) Distribution of the expression level of the gene *GF14* relative to the housekeeping gene *Actin* across all 509 inbreds ordered by the gene expression level. (b) Violinplot of the gene expression level of *GF14* for the eight different germplasm types and (c) for the three MCLUST groups. (d) *P*-value profile from genome-wide association mapping for the gene expression level of the *GF14* gene for all 509 inbreds, (e) for the inbreds of the MCLUST group 1, (f) for the inbreds of the MCLUST group 2, and (g) for the inbreds of the MCLUST group 3. The x-axis shows physical map positions of the SNPs along the 19 chromosomes, the y-axis gives the -$\log_{10}$ *P*-value of the association test. The horizontal dashed and dotted lines indicate the $\alpha = 0.0001$ threshold and the threshold after Bonferroni correction (α=0.05), respectively.
