# Supplementary material for: Seedling development traits in Brassicanapus examined by gene expression analysis and association mapping
Source: BMC Plant Biol. 2015 Jun 9;15:136. doi: 10.1186/s12870-015-0496-3 (PMC4459455; doi:10.1186/s12870-015-0496-3)
Supplement: Additional file 7 — Figure S15-S34. Distribution of the 20 seedling development traits and their P-value profile from genome-wide association mapping (GWAS). [file 12870_2015_496_MOESM7_ESM.pdf]

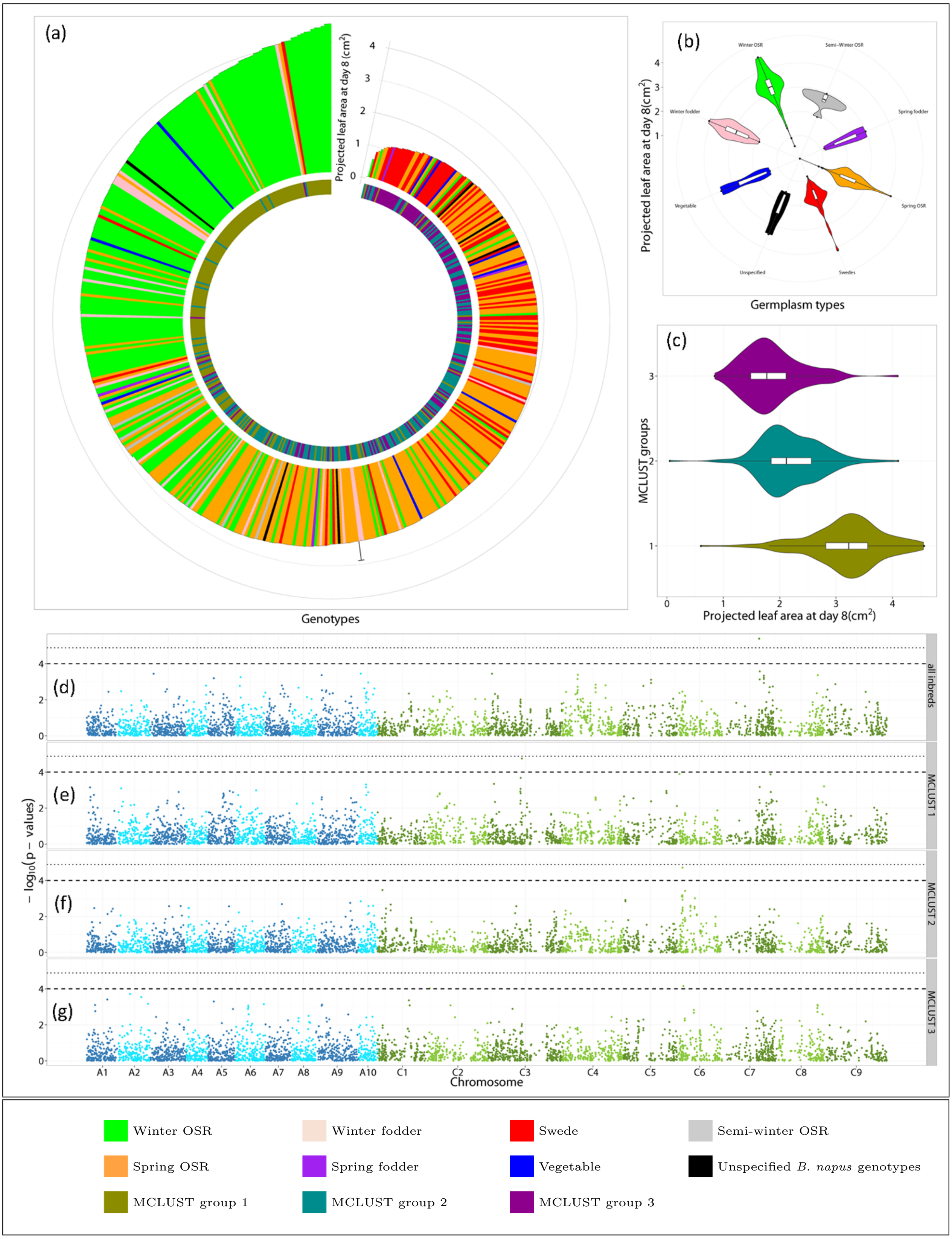

Figure S15. (a) Distribution of the seedling development trait *LA08* across all 509 inbreds ordered by the projected leaf area at day 8 (cm^2). (b) Violinplot of the projected leaf area at day 8 of *LA08* for the eight different germplasm types and (c) for the three MCLUST groups. (d) *P*-value profile from genome-wide association mapping for the seedling development trait *LA08* for all 509 inbreds, (e) for the inbreds of the MCLUST group 1, (f) for the inbreds of the MCLUST group 2, and (g) for the inbreds of the MCLUST group 3. The x-axis shows physical map positions of the SNPs along the 19 chromosomes, the y-axis gives the -$\log_{10}$ *P*-value of the association test. The horizontal dashed and dotted lines indicate the *P*-value = 0.0001 threshold and the threshold after Bonferroni correction (*P*-value=0.05), respectively.

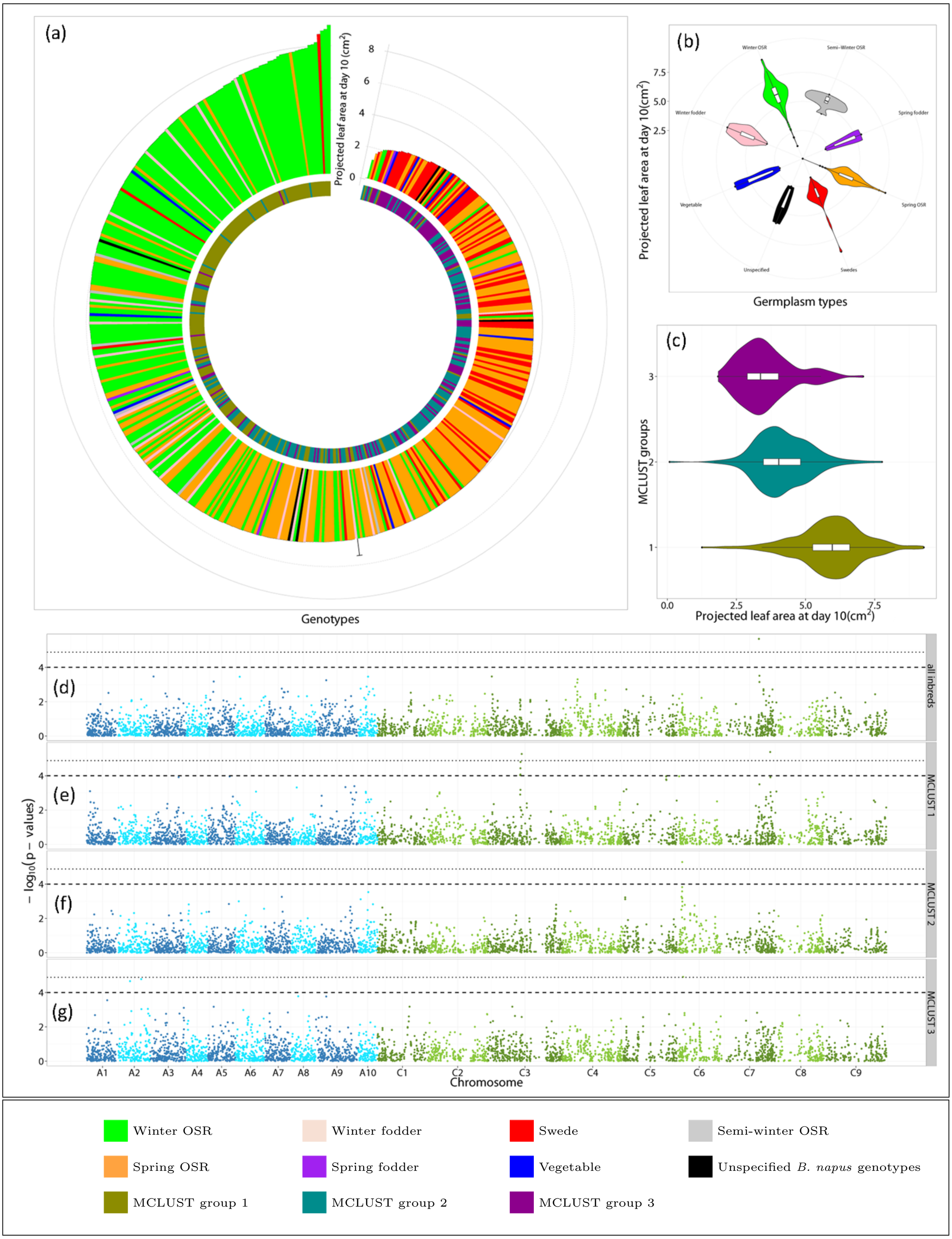

Figure S16. (a) Distribution of the seedling development trait *LA10* across all 509 inbreds ordered by the projected leaf area at day 10 (cm^2). (b) Violinplot of the projected leaf area at day 10 of *LA10* for the eight different germplasm types and (c) for the three MCLUST groups. (d) *P*-value profile from genome-wide association mapping for the seedling development trait *LA10* for all 509 inbreds, (e) for the inbreds of the MCLUST group 1, (f) for the inbreds of the MCLUST group 2, and (g) for the inbreds of the MCLUST group 3. The x-axis shows physical map positions of the SNPs along the 19 chromosomes, the y-axis gives the -$\log_{10}$ *P*-value of the association test. The horizontal dashed and dotted lines indicate the *P*-value = 0.0001 threshold and the threshold after Bonferroni correction (*P*-value=0.05), respectively.

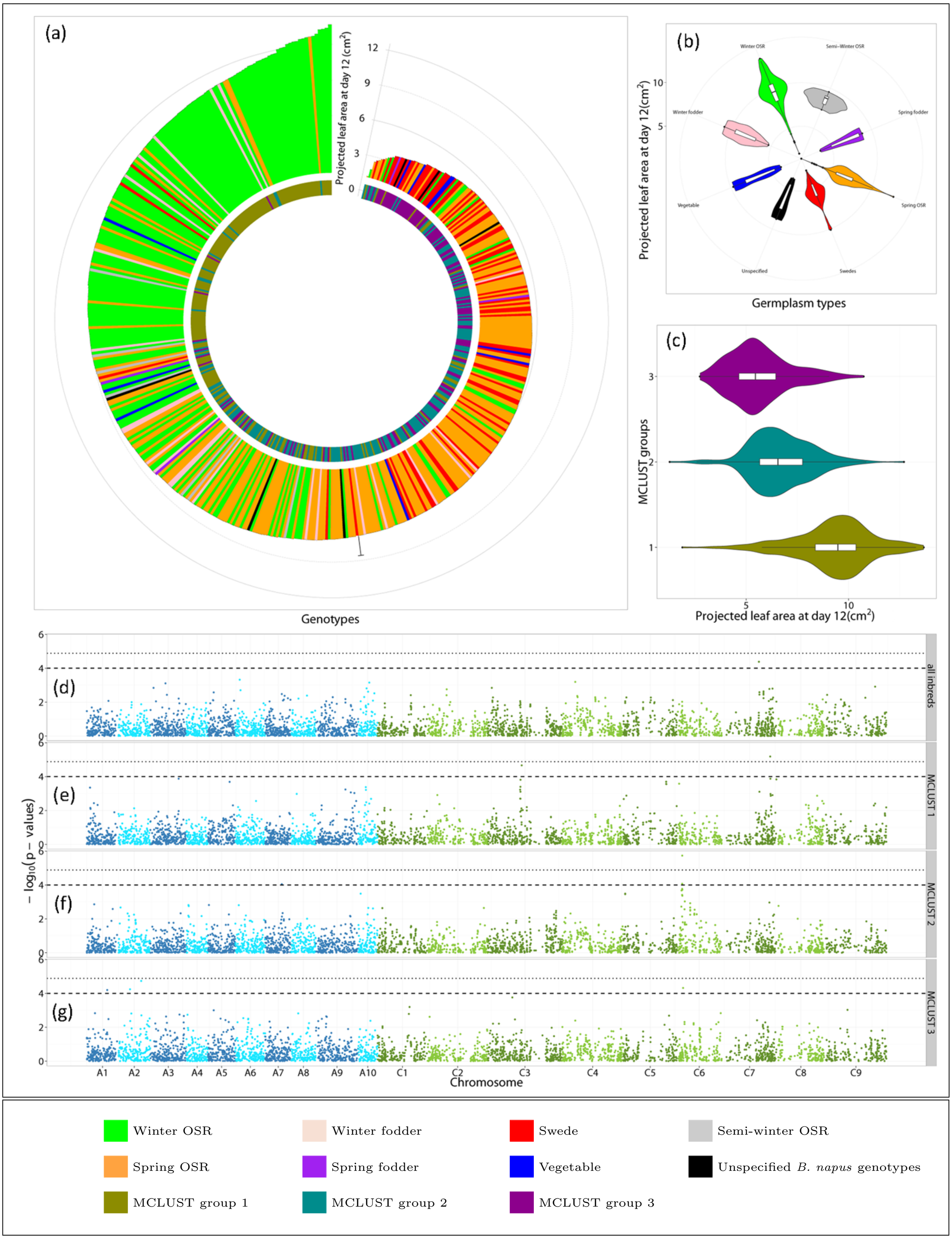

Figure S17. (a) Distribution of the seedling development trait *LA12* across all 509 inbreds ordered by the projected leaf area at day 12 (cm^2). (b) Violinplot of the projected leaf area at day 12 of *LA12* for the eight different germplasm types and (c) for the three MCLUST groups. (d) *P*-value profile from genome-wide association mapping for the seedling development trait *LA12* for all 509 inbreds, (e) for the inbreds of the MCLUST group 1, (f) for the inbreds of the MCLUST group 2, and (g) for the inbreds of the MCLUST group 3. The x-axis shows physical map positions of the SNPs along the 19 chromosomes, the y-axis gives the -$\log_{10}$ *P*-value of the association test. The horizontal dashed and dotted lines indicate the *P*-value = 0.0001 threshold and the threshold after Bonferroni correction (*P*-value=0.05), respectively.

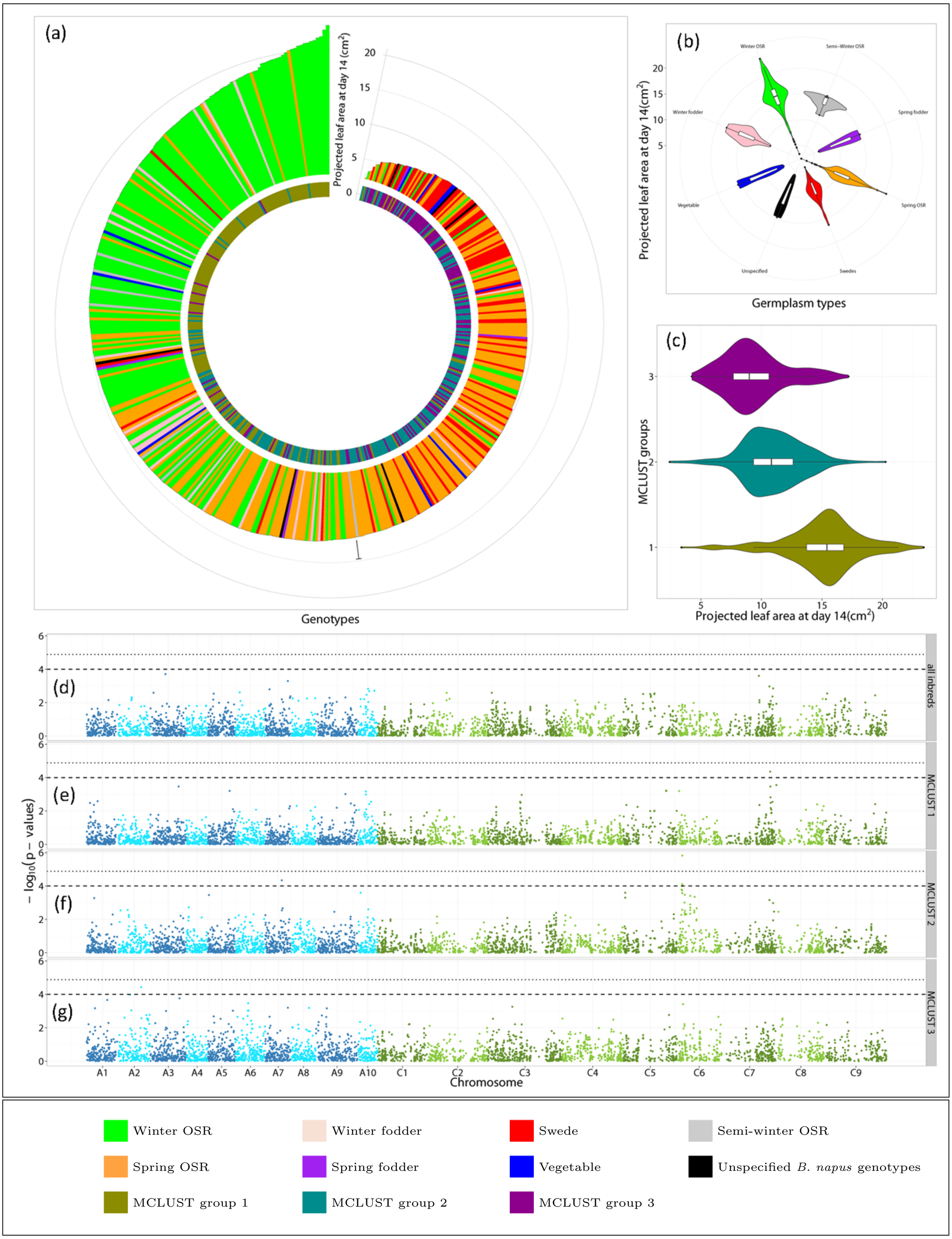

Figure S18. (a) Distribution of the seedling development trait *LA14* across all 509 inbreds ordered by the projected leaf area at day 14 (cm^2). (b) Violinplot of the projected leaf area at day 14 of *LA14* for the eight different germplasm types and (c) for the three MCLUST groups. (d) *P*-value profile from genome-wide association mapping for the seedling development trait *LA14* for all 509 inbreds, (e) for the inbreds of the MCLUST group 1, (f) for the inbreds of the MCLUST group 2, and (g) for the inbreds of the MCLUST group 3. The x-axis shows physical map positions of the SNPs along the 19 chromosomes, the y-axis gives the -$\log_{10}$ *P*-value of the association test. The horizontal dashed and dotted lines indicate the *P*-value = 0.0001 threshold and the threshold after Bonferroni correction (*P*-value=0.05), respectively.

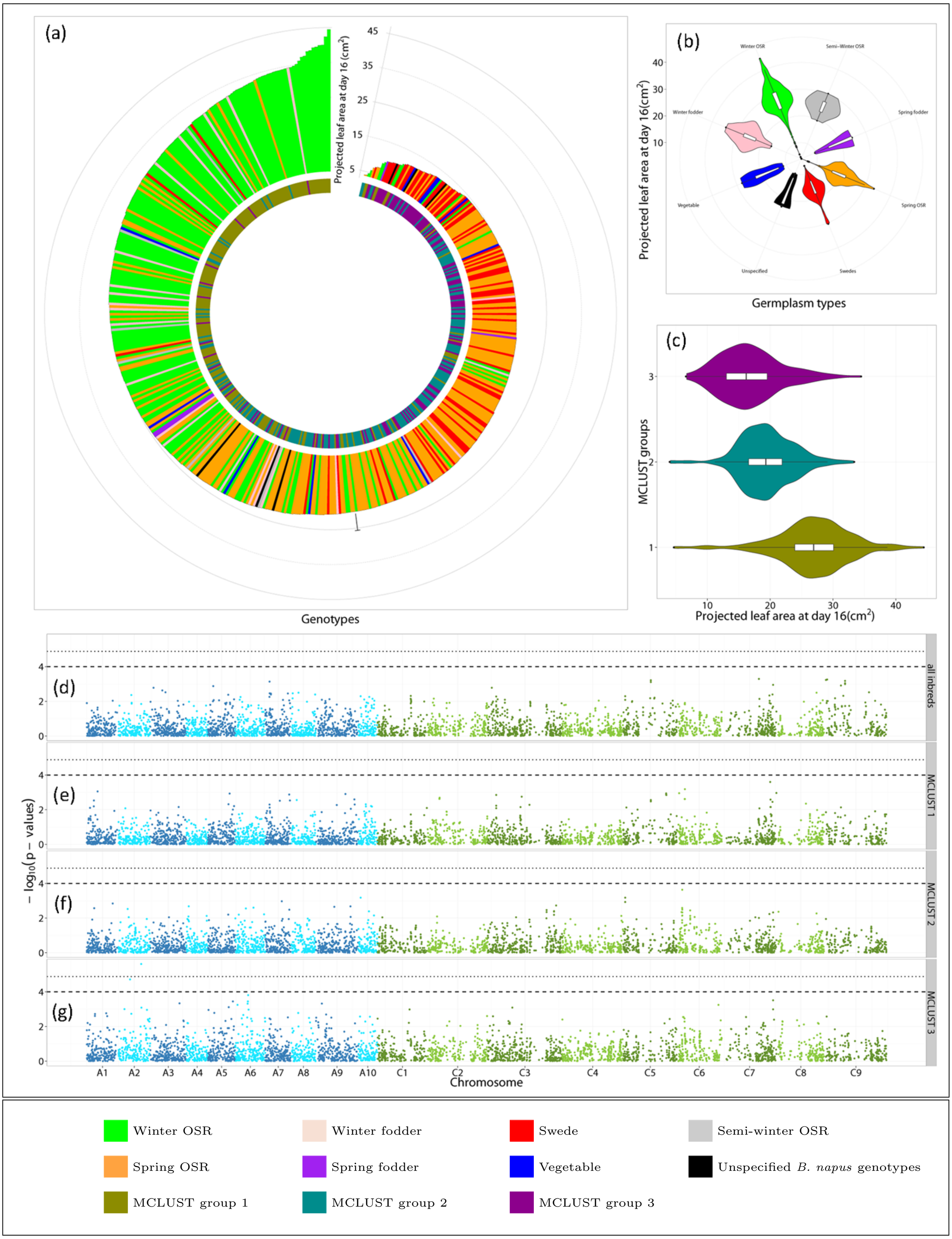

Figure S19. (a) Distribution of the seedling development trait *LA16* across all 509 inbreds ordered by the projected leaf area at day 16 (cm^2). (b) Violinplot of the projected leaf area at day 16 of *LA16* for the eight different germplasm types and (c) for the three MCLUST groups. (d) P-value profile from genome-wide association mapping for the seedling development trait *LA16* for all 509 inbreds, (e) for the inbreds of the MCLUST group 1, (f) for the inbreds of the MCLUST group 2, and (g) for the inbreds of the MCLUST group 3. The x-axis shows physical map positions of the SNPs along the 19 chromosomes, the y-axis gives the -$\log_{10}$ P-value of the association test. The horizontal dashed and dotted lines indicate the P-value = 0.0001 threshold and the threshold after Bonferroni correction (P-value=0.05), respectively.

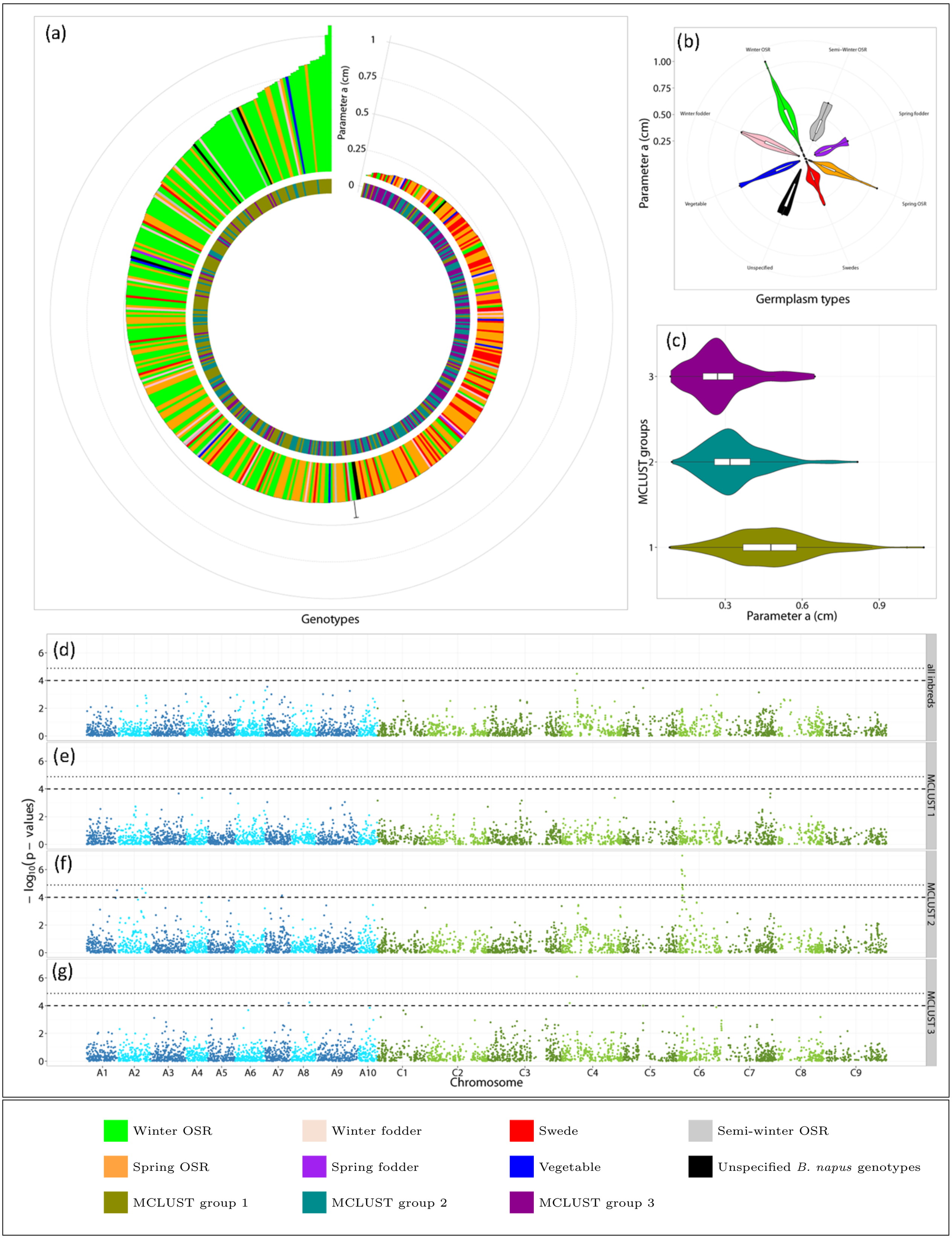

Figure S20. (a) Distribution of the seedling development trait *PRA* across all 509 inbreds ordered by the parameter a (cm^2). (b) Violinplot of the parameter a of *PRA* for the eight different germplasm types and (c) for the three MCLUST groups. (d) *P*-value profile from genome-wide association mapping for the seedling development trait *PRA* for all 509 inbreds, (e) for the inbreds of the MCLUST group 1, (f) for the inbreds of the MCLUST group 2, and (g) for the inbreds of the MCLUST group 3. The x-axis shows physical map positions of the SNPs along the 19 chromosomes, the y-axis gives the -$\log_{10}$ *P*-value of the association test. The horizontal dashed and dotted lines indicate the *P*-value = 0.0001 threshold and the threshold after Bonferroni correction (*P*-value=0.05), respectively.

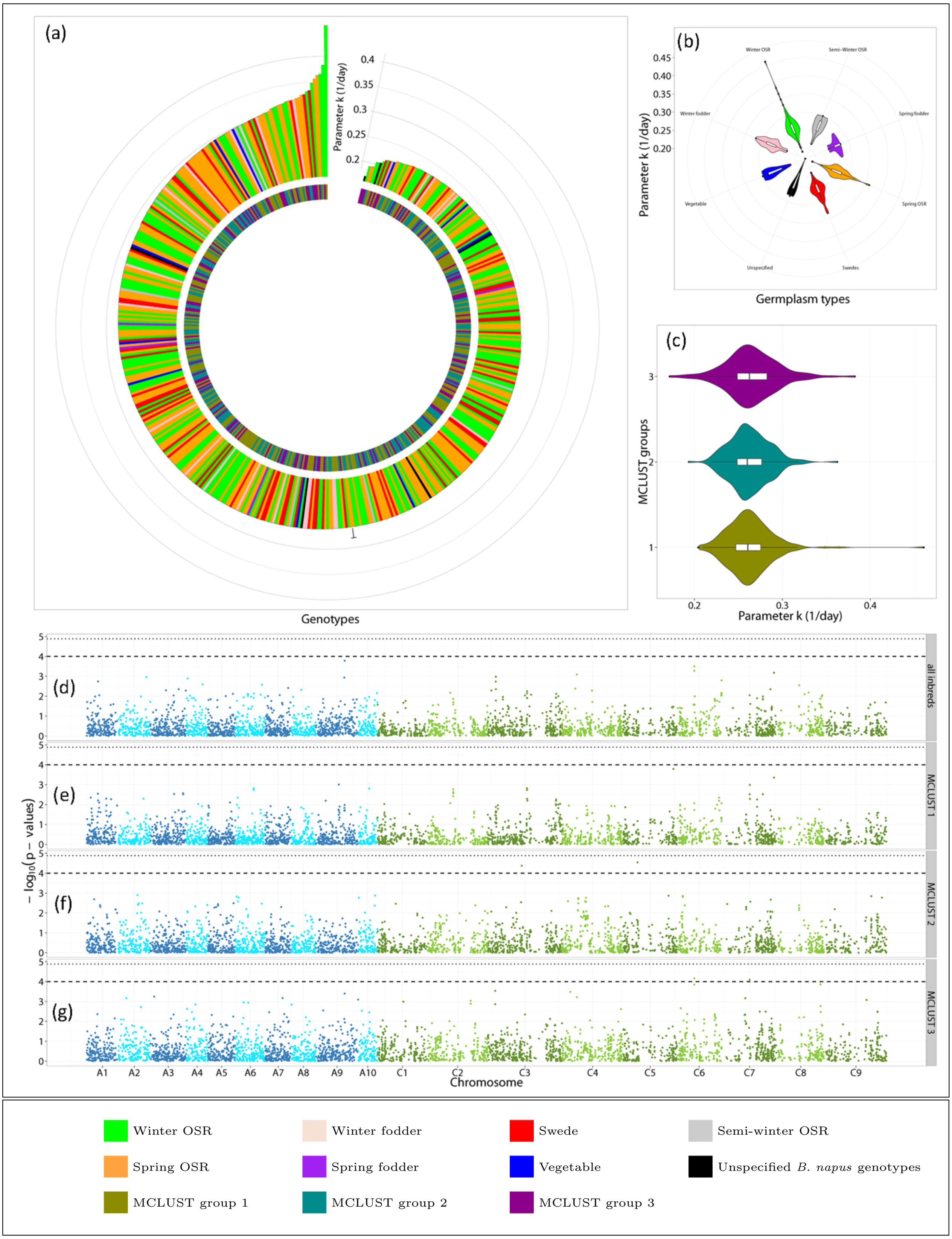

Figure S21. (a) Distribution of the seedling development trait *PRK* across all 509 inbreds ordered by the parameter k (1/day). (b) Violinplot of the parameter k of *PRK* for the eight different germplasm types and (c) for the three MCLUST groups. (d) *P*-value profile from genome-wide association mapping for the seedling development trait *PRK* for all 509 inbreds, (e) for the inbreds of the MCLUST group 1, (f) for the inbreds of the MCLUST group 2, and (g) for the inbreds of the MCLUST group 3. The x-axis shows physical map positions of the SNPs along the 19 chromosomes, the y-axis gives the -$\log_{10}$ *P*-value of the association test. The horizontal dashed and dotted lines indicate the *P*-value = 0.0001 threshold and the threshold after Bonferroni correction (*P*-value=0.05), respectively.

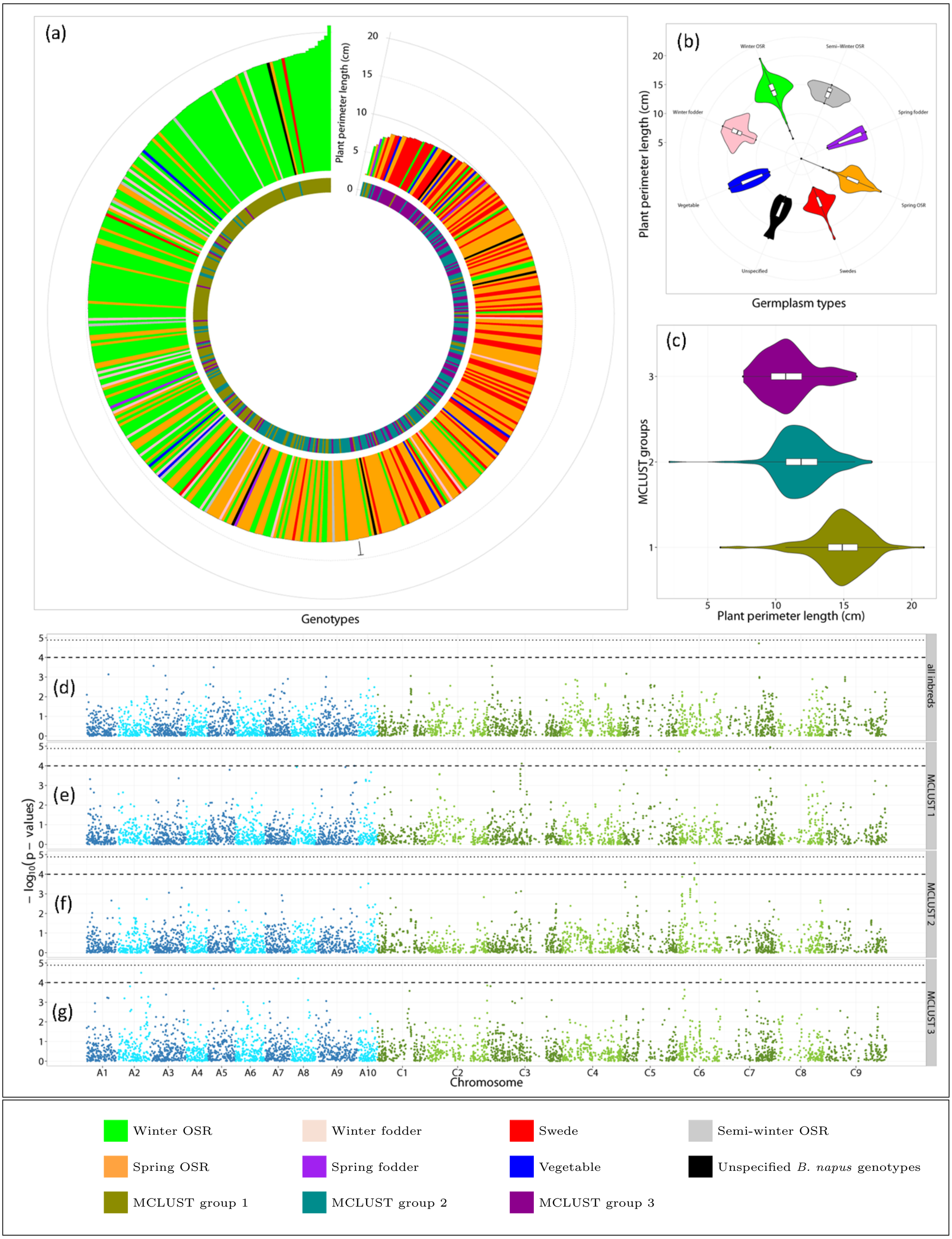

Figure S22. (a) Distribution of the seedling development trait *PER* across all 509 inbreds ordered by the plant perimeter length (cm). (b) Violinplot of the plant perimeter length of *PER* for the eight different germplasm types and (c) for the three MCLUST groups. (d) *P*-value profile from genome-wide association mapping for the seedling development trait *PER* for all 509 inbreds, (e) for the inbreds of the MCLUST group 1, (f) for the inbreds of the MCLUST group 2, and (g) for the inbreds of the MCLUST group 3. The x-axis shows physical map positions of the SNPs along the 19 chromosomes, the y-axis gives the -$\log_{10}$ *P*-value of the association test. The horizontal dashed and dotted lines indicate the *P*-value = 0.0001 threshold and the threshold after Bonferroni correction (*P*-value=0.05), respectively.

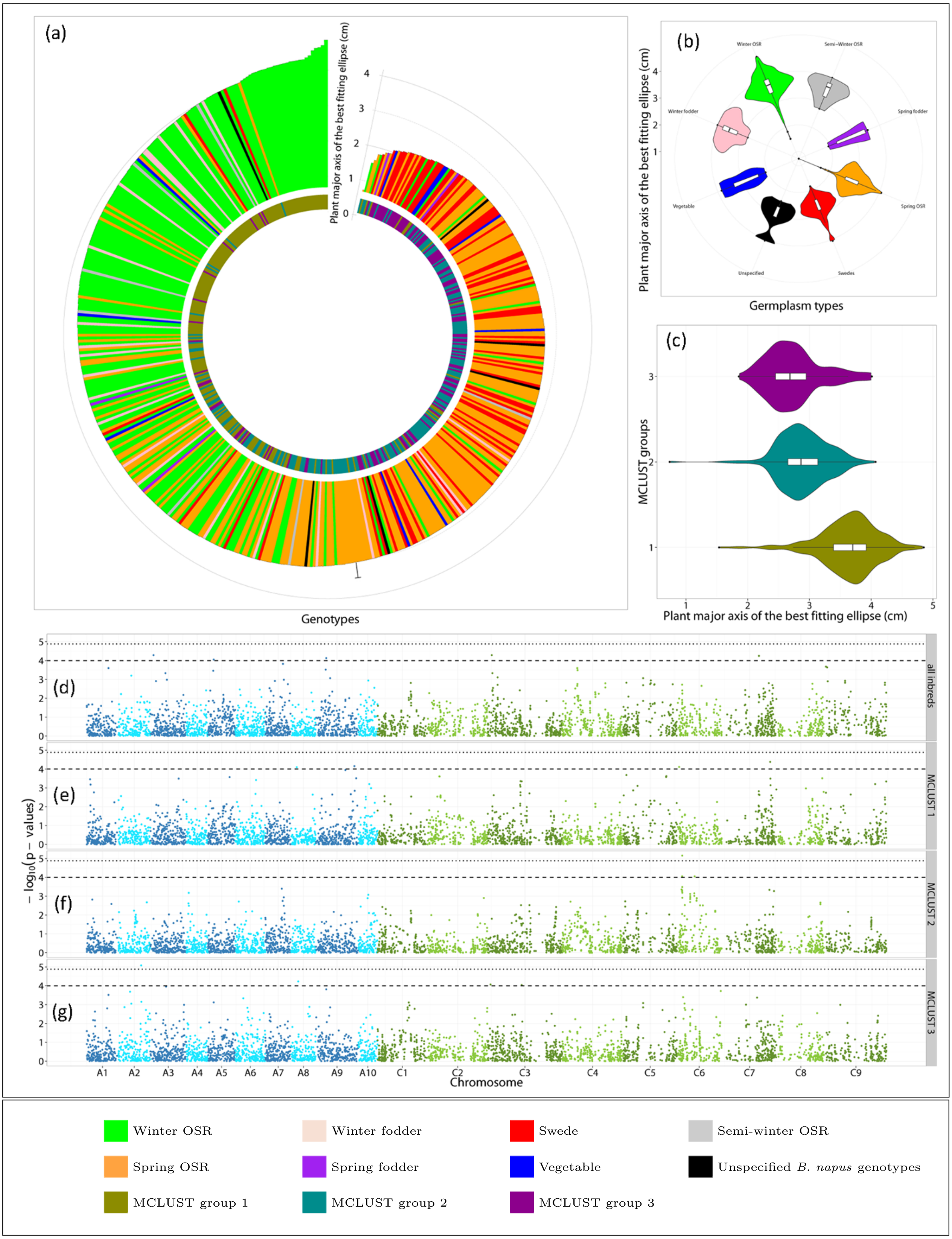

Figure S23. (a) Distribution of the seedling development trait *MAJ* across all 509 inbreds ordered by the plant major axis of the best fitting ellipse (cm). (b) Violinplot of the plant major axis of the best fitting ellipse of *MAJ* for the eight different germplasm types and (c) for the three MCLUST groups. (d) P-value profile from genome-wide association mapping for the seedling development trait *MAJ* for all 509 inbreds, (e) for the inbreds of the MCLUST group 1, (f) for the inbreds of the MCLUST group 2, and (g) for the inbreds of the MCLUST group 3. The x-axis shows physical map positions of the SNPs along the 19 chromosomes, the y-axis gives the -$\log_{10}$ P-value of the association test. The horizontal dashed and dotted lines indicate the P-value = 0.0001 threshold and the threshold after Bonferroni correction (P-value=0.05), respectively.

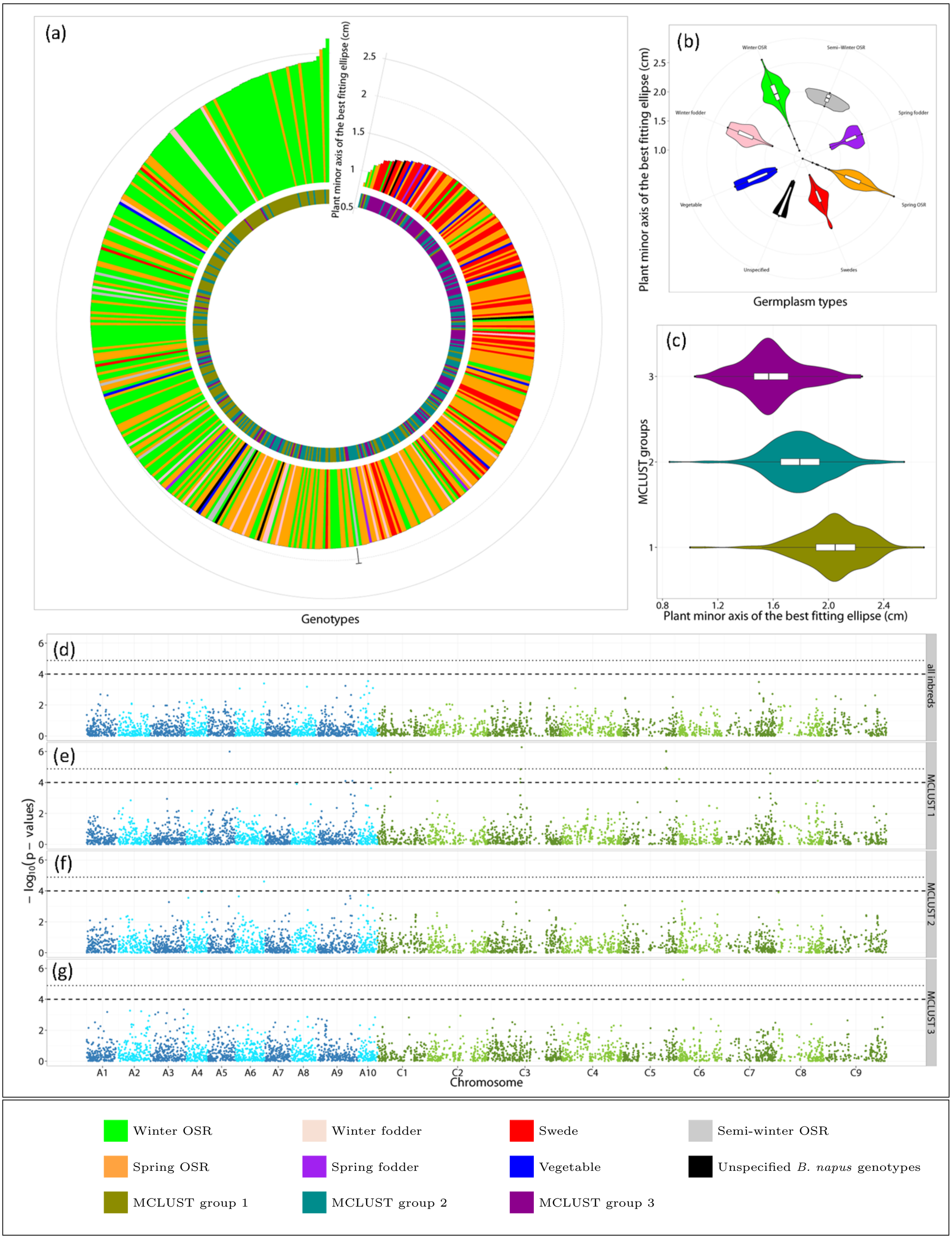

Figure S24. (a) Distribution of the seedling development trait *MIN* across all 509 inbreds ordered by the plant minor axis of the best fitting ellipse (cm). (b) Violinplot of the plant minor axis of the best fitting ellipse of *MIN* for the eight different germplasm types and (c) for the three MCLUST groups. (d) *P*-value profile from genome-wide association mapping for the seedling development trait *MIN* for all 509 inbreds, (e) for the inbreds of the MCLUST group 1, (f) for the inbreds of the MCLUST group 2, and (g) for the inbreds of the MCLUST group 3. The x-axis shows physical map positions of the SNPs along the 19 chromosomes, the y-axis gives the -$\log_{10}$ *P*-value of the association test. The horizontal dashed and dotted lines indicate the *P*-value = 0.0001 threshold and the threshold after Bonferroni correction (*P*-value=0.05), respectively.

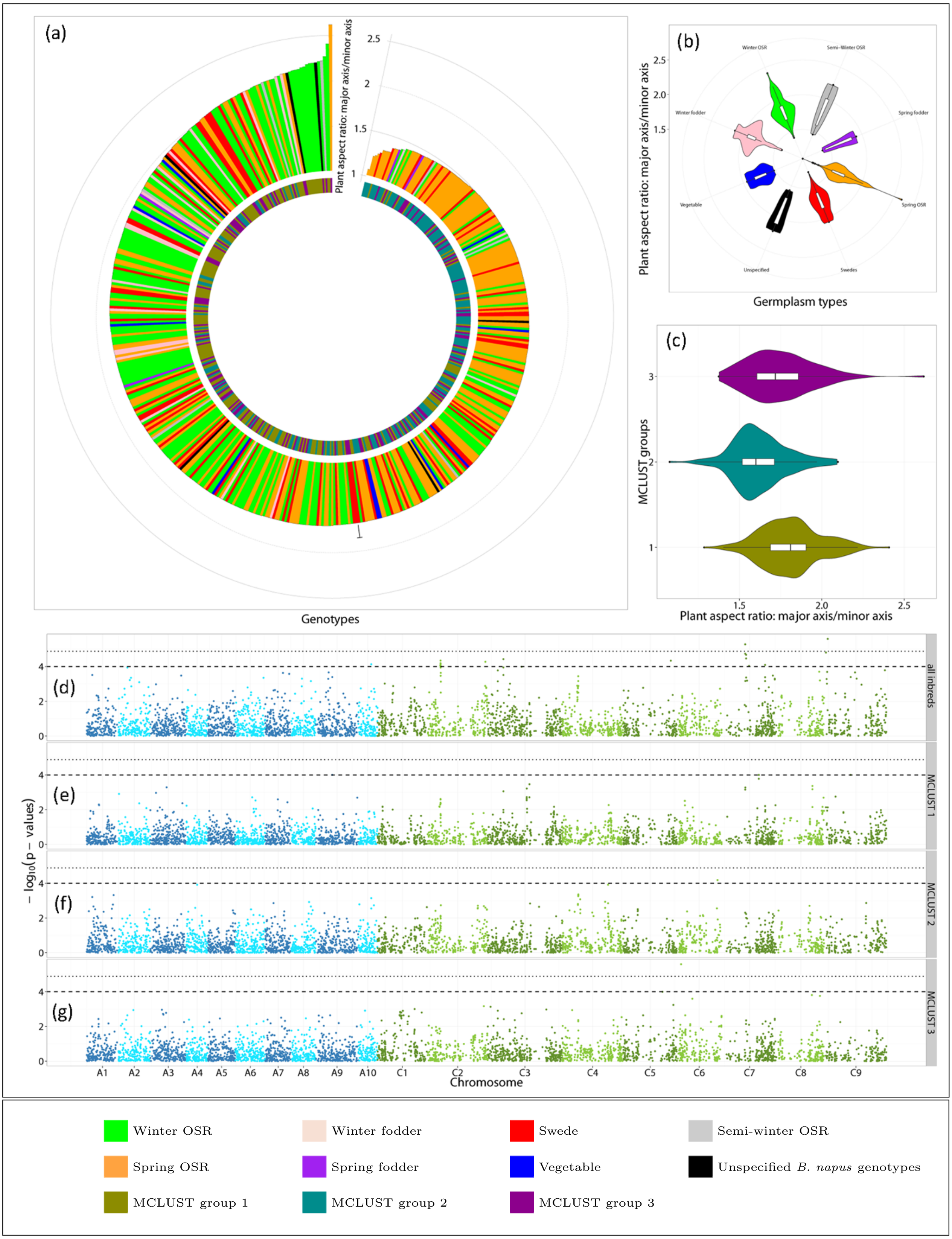

Figure S25. (a) Distribution of the seedling development trait *ASR* across all 509 inbreds ordered by the plant aspect ratio: major axis/minor axis. (b) Violinplot of the plant aspect ratio: major axis/minor axis of *ASR* for the eight different germplasm types and (c) for the three MCLUST groups. (d) P-value profile from genome-wide association mapping for the seedling development trait *ASR* for all 509 inbreds, (e) for the inbreds of the MCLUST group 1, (f) for the inbreds of the MCLUST group 2, and (g) for the inbreds of the MCLUST group 3. The x-axis shows physical map positions of the SNPs along the 19 chromosomes, the y-axis gives the -$\log_{10}$ P-value of the association test. The horizontal dashed and dotted lines indicate the P-value = 0.0001 threshold and the threshold after Bonferroni correction (P-value=0.05), respectively.

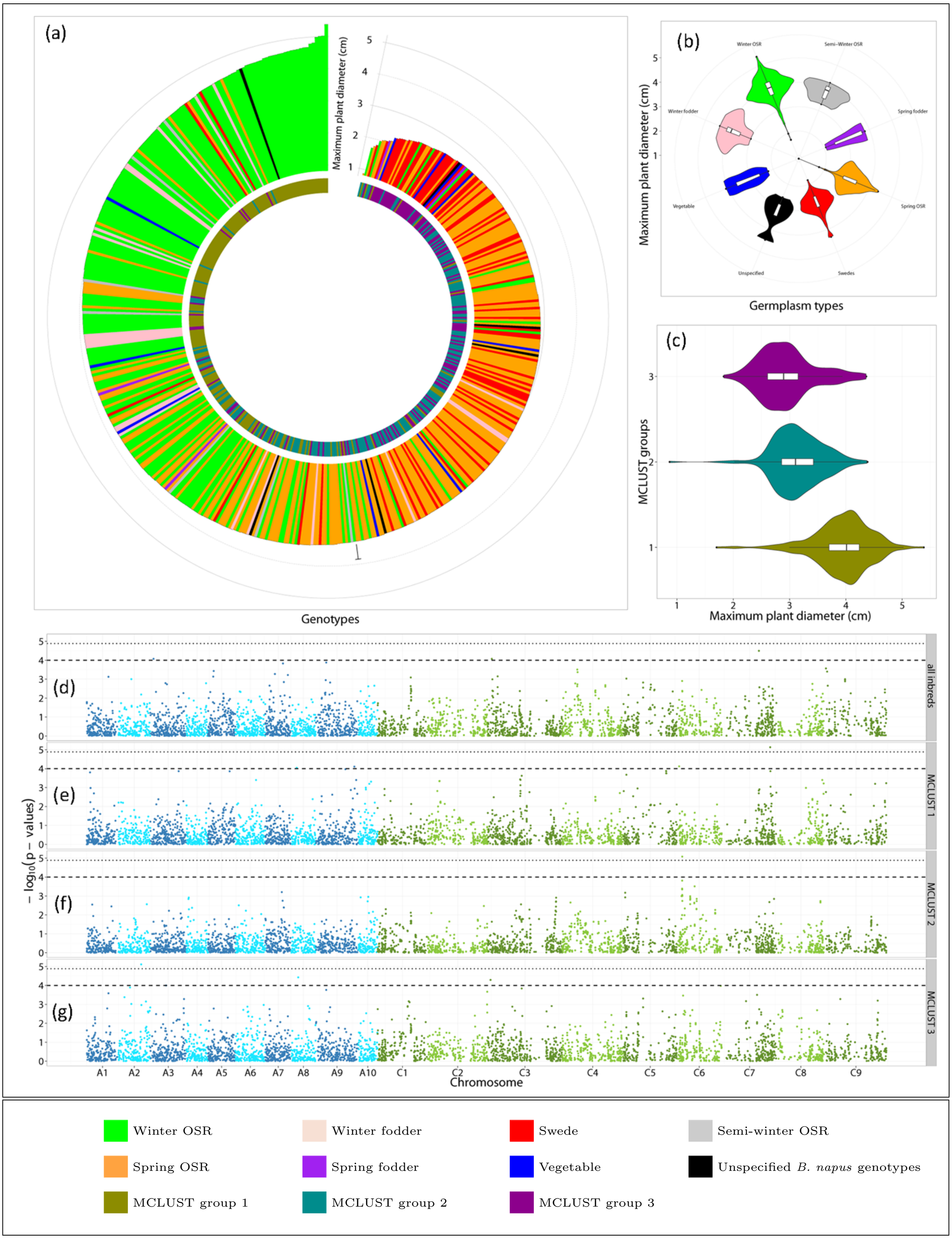

Figure S26. (a) Distribution of the seedling development trait *MAD* across all 509 inbreds ordered by the maximum plant diameter (cm). (b) Violinplot of the maximum plant diameter of *MAD* for the eight different germplasm types and (c) for the three MCLUST groups. (d) *P*-value profile from genome-wide association mapping for the seedling development trait *MAD* for all 509 inbreds, (e) for the inbreds of the MCLUST group 1, (f) for the inbreds of the MCLUST group 2, and (g) for the inbreds of the MCLUST group 3. The x-axis shows physical map positions of the SNPs along the 19 chromosomes, the y-axis gives the -$\log_{10}$ *P*-value of the association test. The horizontal dashed and dotted lines indicate the *P*-value = 0.0001 threshold and the threshold after Bonferroni correction (*P*-value=0.05), respectively.

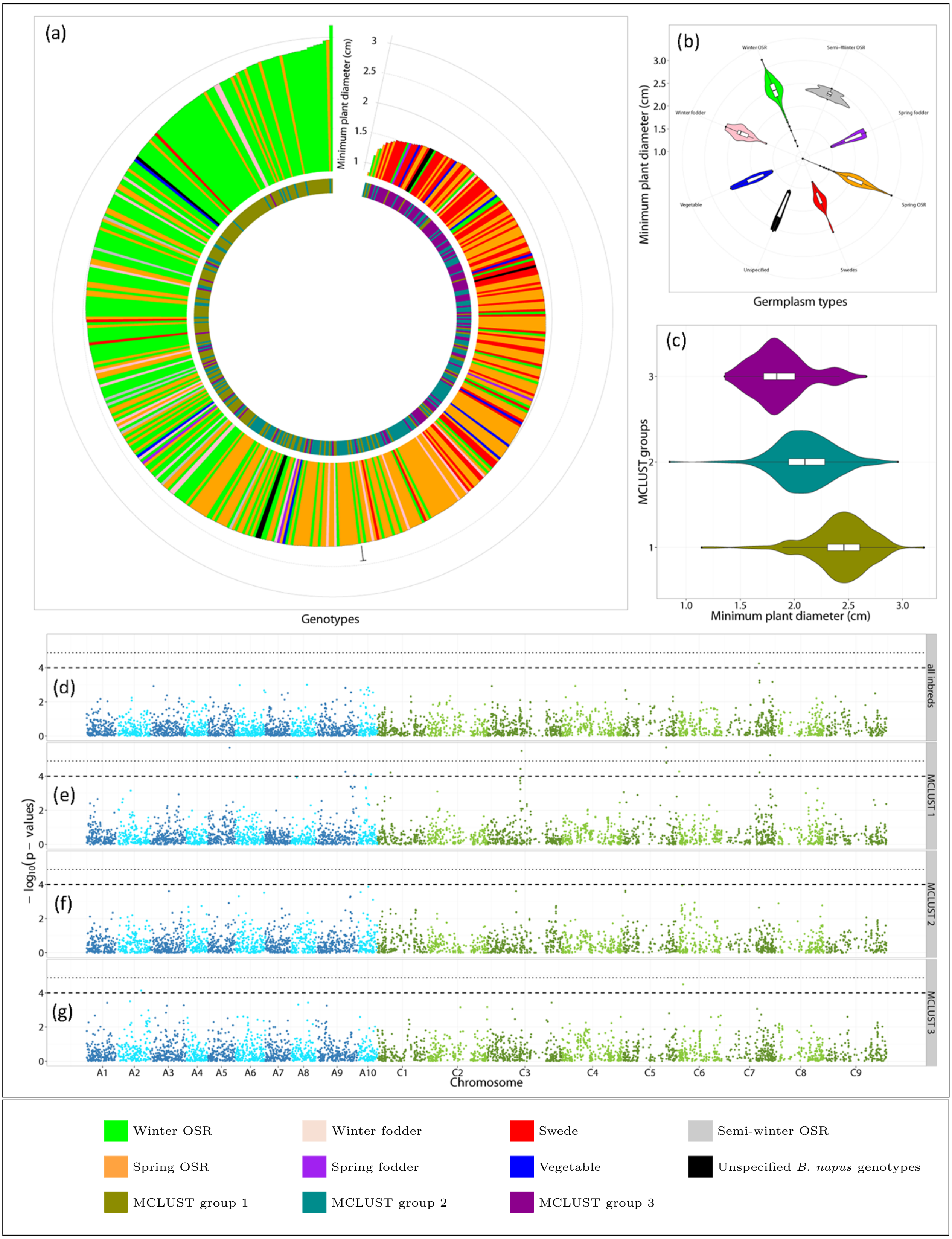

Figure S27. (a) Distribution of the seedling development trait *MID* across all 509 inbreds ordered by the minimum plant diameter (cm). (b) Violinplot of the minimum plant diameter of *MID* for the eight different germplasm types and (c) for the three MCLUST groups. (d) *P*-value profile from genome-wide association mapping for the seedling development trait *MID* for all 509 inbreds, (e) for the inbreds of the MCLUST group 1, (f) for the inbreds of the MCLUST group 2, and (g) for the inbreds of the MCLUST group 3. The x-axis shows physical map positions of the SNPs along the 19 chromosomes, the y-axis gives the -log$_{10}$ *P*-value of the association test. The horizontal dashed and dotted lines indicate the *P*-value = 0.0001 threshold and the threshold after Bonferroni correction (*P*-value=0.05), respectively.

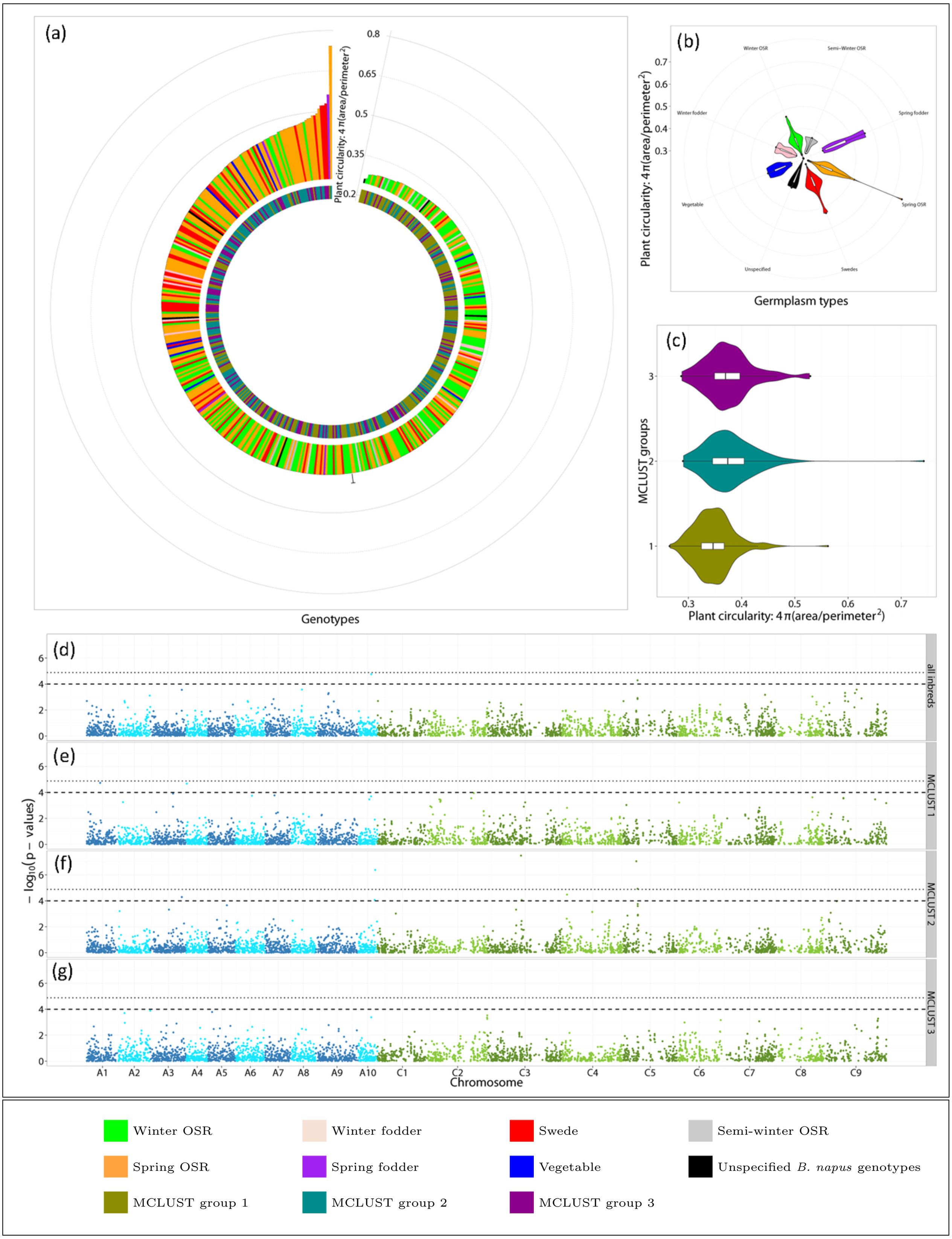

Figure S28. (a) Distribution of the seedling development trait *CIR* across all 509 inbreds ordered by the plant circularity ($4\pi(\text{area}/\text{perimeter}^2)$). (b) Violinplot of the plant circularity of *CIR* for the eight different germplasm types and (c) for the three MCLUST groups. (d) *P*-value profile from genome-wide association mapping for the seedling development trait *CIR* for all 509 inbreds, (e) for the inbreds of the MCLUST group 1, (f) for the inbreds of the MCLUST group 2, and (g) for the inbreds of the MCLUST group 3. The x-axis shows physical map positions of the SNPs along the 19 chromosomes, the y-axis gives the -$\log_{10}$ *P*-value of the association test. The horizontal dashed and dotted lines indicate the *P*-value = 0.0001 threshold and the threshold after Bonferroni correction (*P*-value=0.05), respectively.

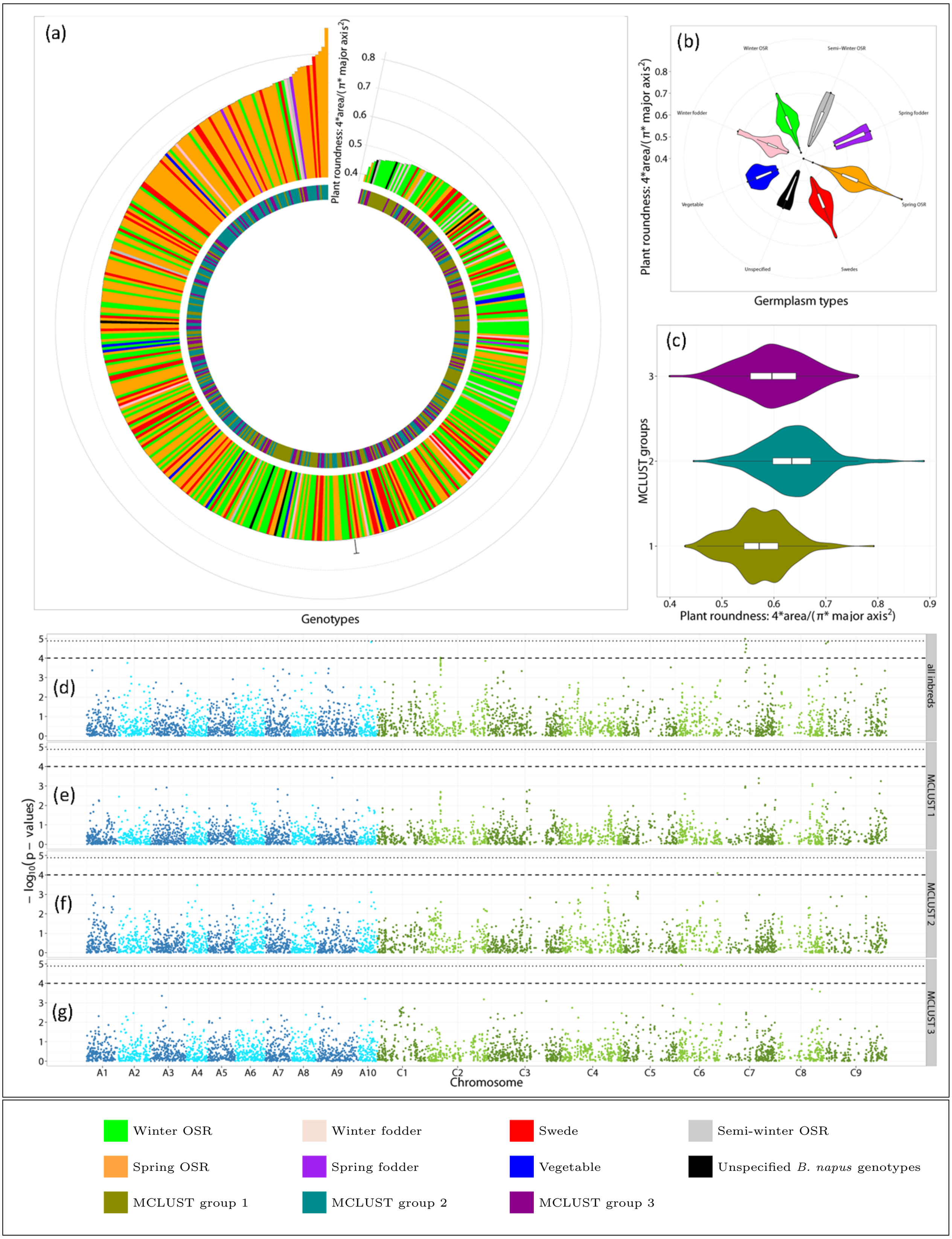

Figure S29. (a) Distribution of the seedling development trait *ROU* across all 509 inbreds ordered by the plant roundness (4*area/(π*major axis^2)). (b) Violinplot of the plant roundness of *ROU* for the eight different germplasm types and (c) for the three MCLUST groups. (d) *P*-value profile from genome-wide association mapping for the seedling development trait *ROU* for all 509 inbreds, (e) for the inbreds of the MCLUST group 1, (f) for the inbreds of the MCLUST group 2, and (g) for the inbreds of the MCLUST group 3. The x-axis shows physical map positions of the SNPs along the 19 chromosomes, the y-axis gives the -$\log_{10}$ *P*-value of the association test. The horizontal dashed and dotted lines indicate the *P*-value = 0.0001 threshold and the threshold after Bonferroni correction (*P*-value=0.05), respectively.

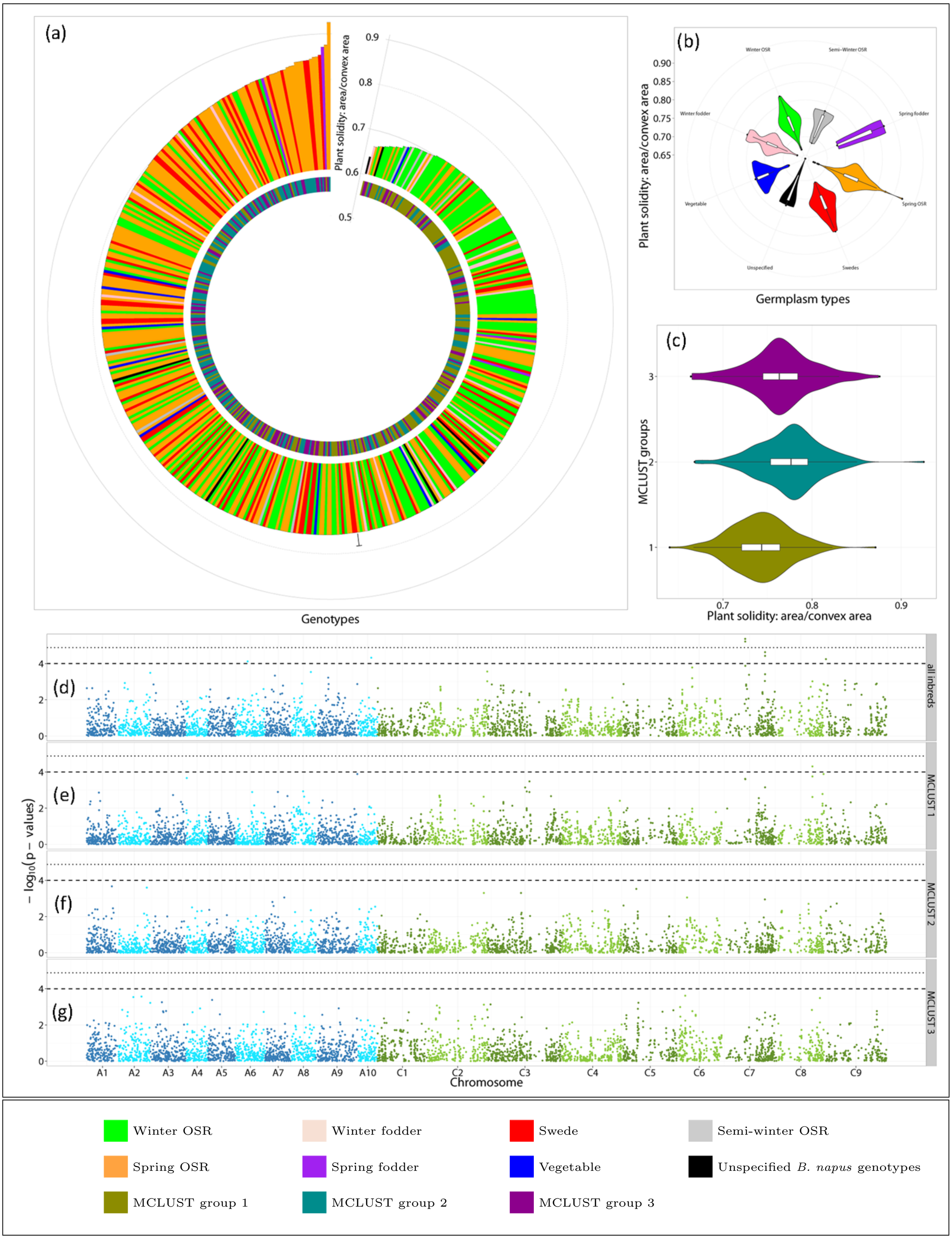

Figure S30. (a) Distribution of the seedling development trait *SOY* across all 509 inbreds ordered by the plant solidity (area/convex area). (b) Violinplot of the plant solidity of *SOY* for the eight different germplasm types and (c) for the three MCLUST groups. (d) *P*-value profile from genome-wide association mapping for the seedling development trait *SOY* for all 509 inbreds, (e) for the inbreds of the MCLUST group 1, (f) for the inbreds of the MCLUST group 2, and (g) for the inbreds of the MCLUST group 3. The x-axis shows physical map positions of the SNPs along the 19 chromosomes, the y-axis gives the -$\log_{10}$ *P*-value of the association test. The horizontal dashed and dotted lines indicate the *P*-value = 0.0001 threshold and the threshold after Bonferroni correction (*P*-value=0.05), respectively.

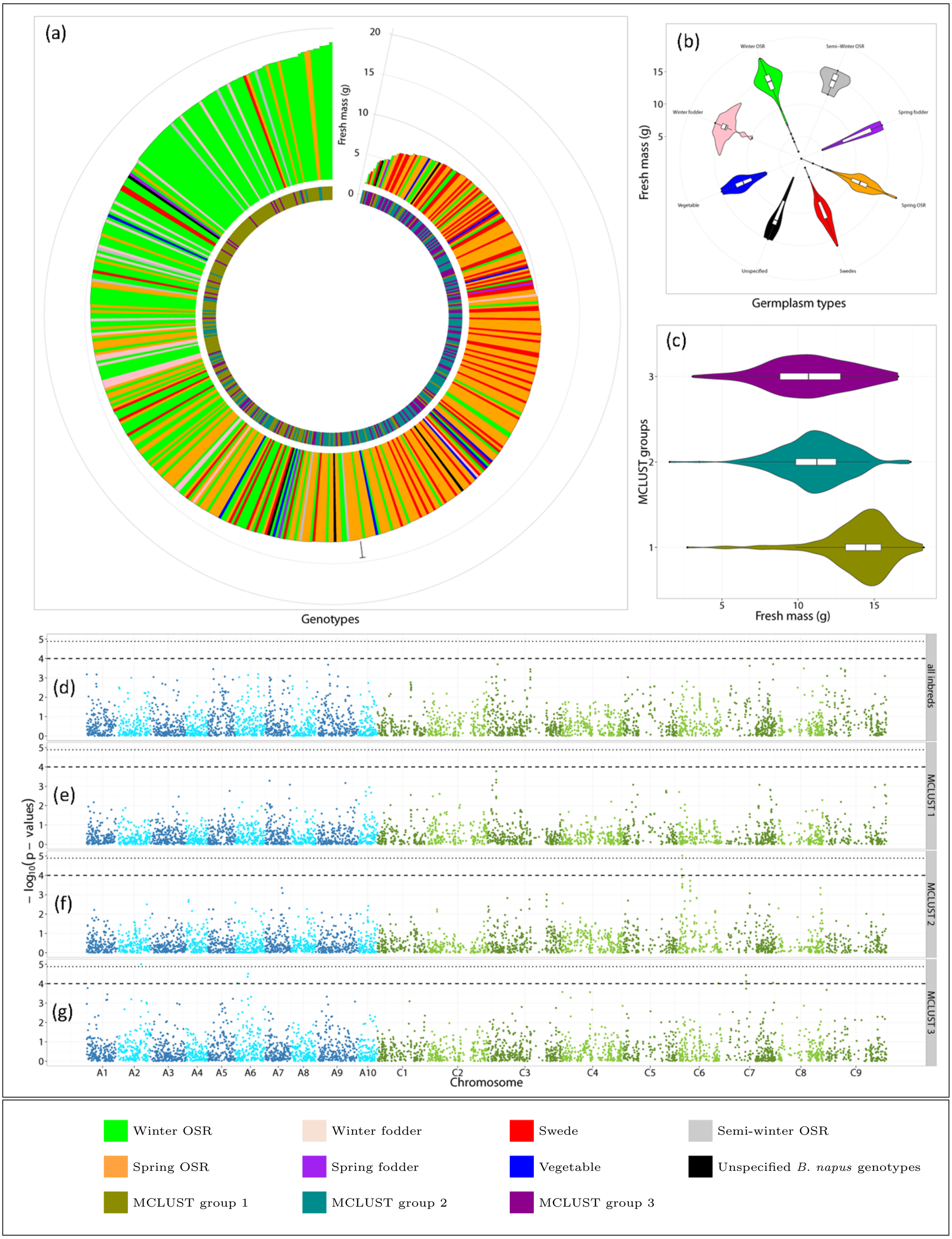

Figure S31. (a) Distribution of the seedling development trait *FHM* across all 509 inbreds ordered by the fresh mass (g). (b) Violinplot of the fresh mass of *FHM* for the eight different germplasm types and (c) for the three MCLUST groups. (d) *P*-value profile from genome-wide association mapping for the seedling development trait *FHM* for all 509 inbreds, (e) for the inbreds of the MCLUST group 1, (f) for the inbreds of the MCLUST group 2, and (g) for the inbreds of the MCLUST group 3. The x-axis shows physical map positions of the SNPs along the 19 chromosomes, the y-axis gives the -$\log_{10}$ *P*-value of the association test. The horizontal dashed and dotted lines indicate the *P*-value = 0.0001 threshold and the threshold after Bonferroni correction (*P*-value=0.05), respectively.

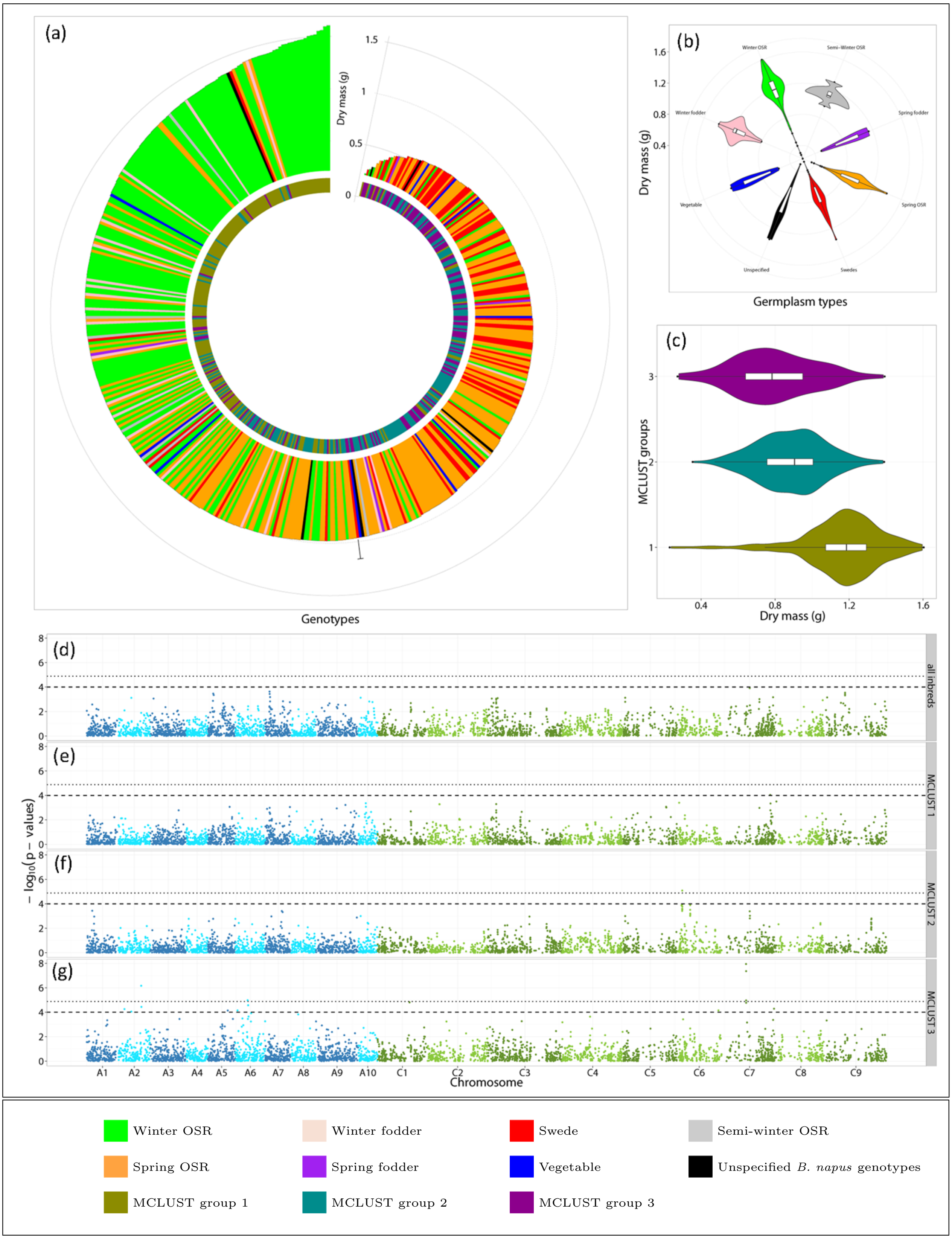

Figure S32. (a) Distribution of the seedling development trait *DYM* across all 509 inbreds ordered by the dry mass (g). (b) Violinplot of the dry mass of *DYM* for the eight different germplasm types and (c) for the three MCLUST groups. (d) P-value profile from genome-wide association mapping for the seedling development trait *DYM* for all 509 inbreds, (e) for the inbreds of the MCLUST group 1, (f) for the inbreds of the MCLUST group 2, and (g) for the inbreds of the MCLUST group 3. The x-axis shows physical map positions of the SNPs along the 19 chromosomes, the y-axis gives the -$\log_{10}$ P-value of the association test. The horizontal dashed and dotted lines indicate the P-value $= 0.0001$ threshold and the threshold after Bonferroni correction (P-value=0.05), respectively.

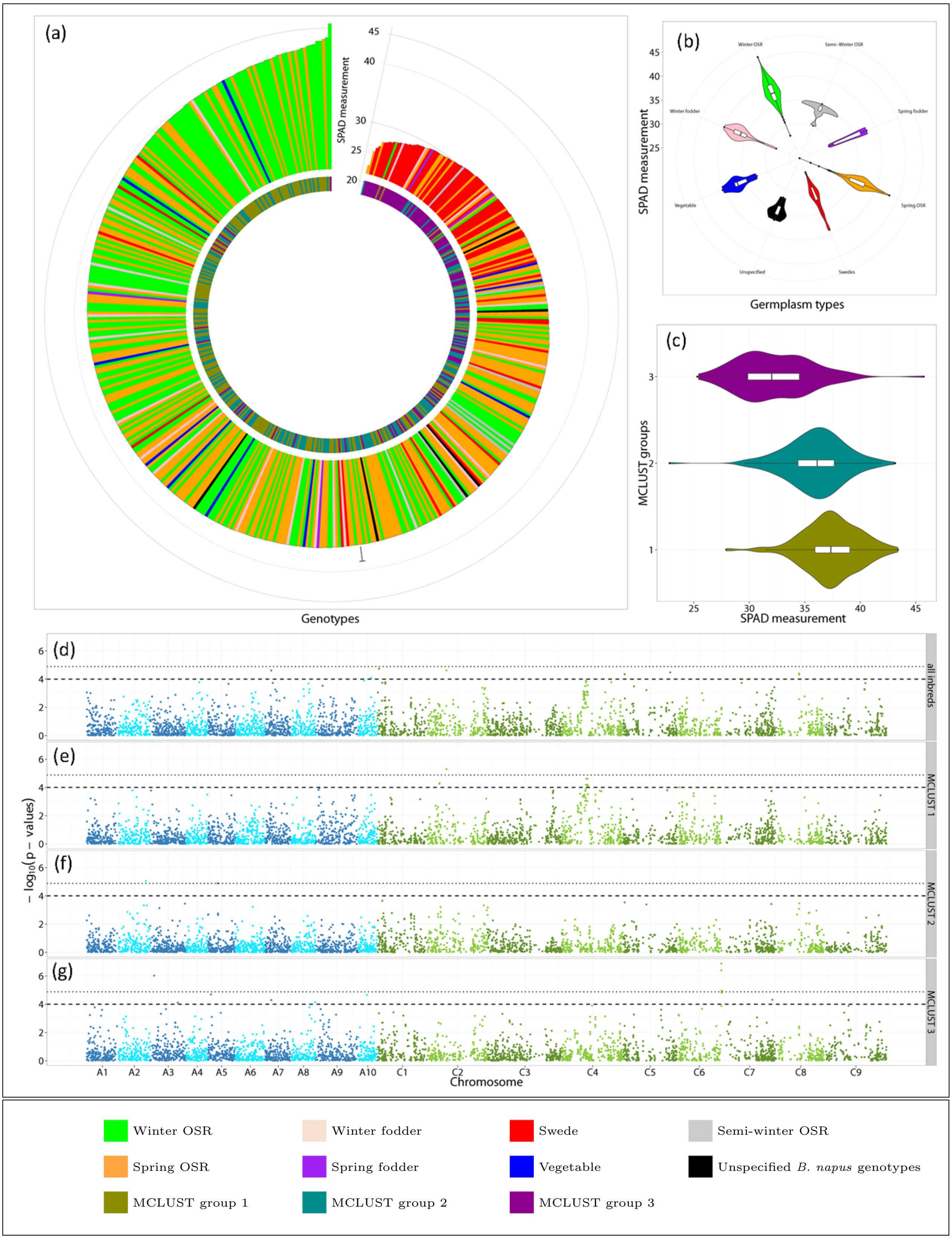

Figure S33. (a) Distribution of the seedling development trait *SPD* across all 509 inbreds ordered by the SPAD measurement value. (b) Violinplot of the SPAD measurement value of *SPD* for the eight different germplasm types and (c) for the three MCLUST groups. (d) *P*-value profile from genome-wide association mapping for the seedling development trait *SPD* for all 509 inbreds, (e) for the inbreds of the MCLUST group 1, (f) for the inbreds of the MCLUST group 2, and (g) for the inbreds of the MCLUST group 3. The x-axis shows physical map positions of the SNPs along the 19 chromosomes, the y-axis gives the -$\log_{10}$ *P*-value of the association test. The horizontal dashed and dotted lines indicate the *P*-value = 0.0001 threshold and the threshold after Bonferroni correction (*P*-value=0.05), respectively.

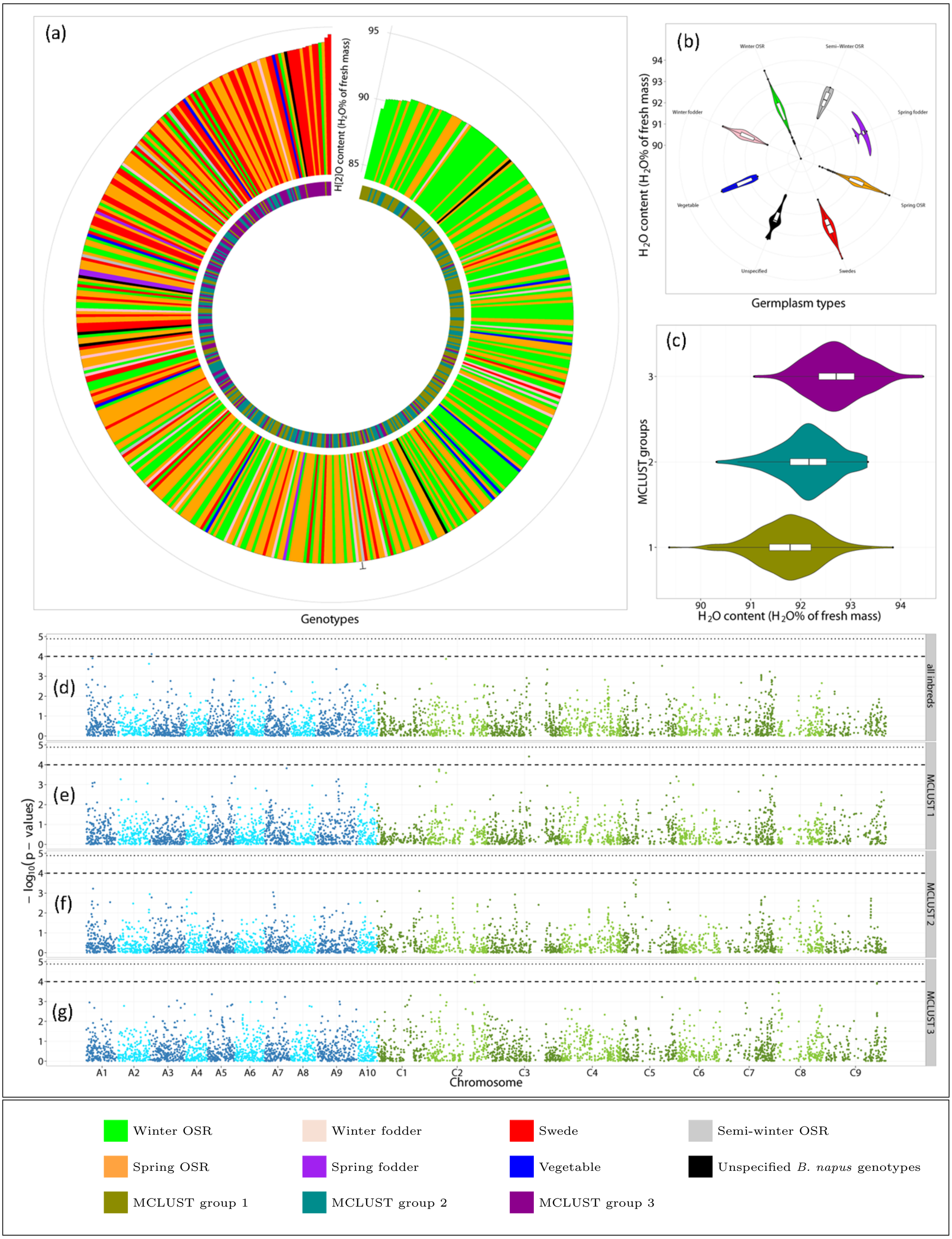

Figure S34. (a) Distribution of the seedling development trait *H2O* across all 509 inbreds ordered by the H_2O content (% of fresh mass). (b) Violinplot of the H_2O content of *H2O* for the eight different germplasm types and (c) for the three MCLUST groups. (d) *P*-value profile from genome-wide association mapping for the seedling development trait *H2O* for all 509 inbreds, (e) for the inbreds of the MCLUST group 1, (f) for the inbreds of the MCLUST group 2, and (g) for the inbreds of the MCLUST group 3. The x-axis shows physical map positions of the SNPs along the 19 chromosomes, the y-axis gives the -$\log_{10}$ *P*-value of the association test. The horizontal dashed and dotted lines indicate the *P*-value = 0.0001 threshold and the threshold after Bonferroni correction (*P*-value=0.05), respectively.
