# Supplementary material for: Seedling development traits in Brassicanapus examined by gene expression analysis and association mapping
Source: BMC Plant Biol. 2015 Jun 9;15:136. doi: 10.1186/s12870-015-0496-3 (PMC4459455; doi:10.1186/s12870-015-0496-3)
Supplement: Additional file 8 — Figure S35-S38. Correlations of the seedling development traits and the gene expression levels across all 509 inbreds as well as the inbreds of the MCLUST groups 1 to 3. [file 12870_2015_496_MOESM8_ESM.pdf]

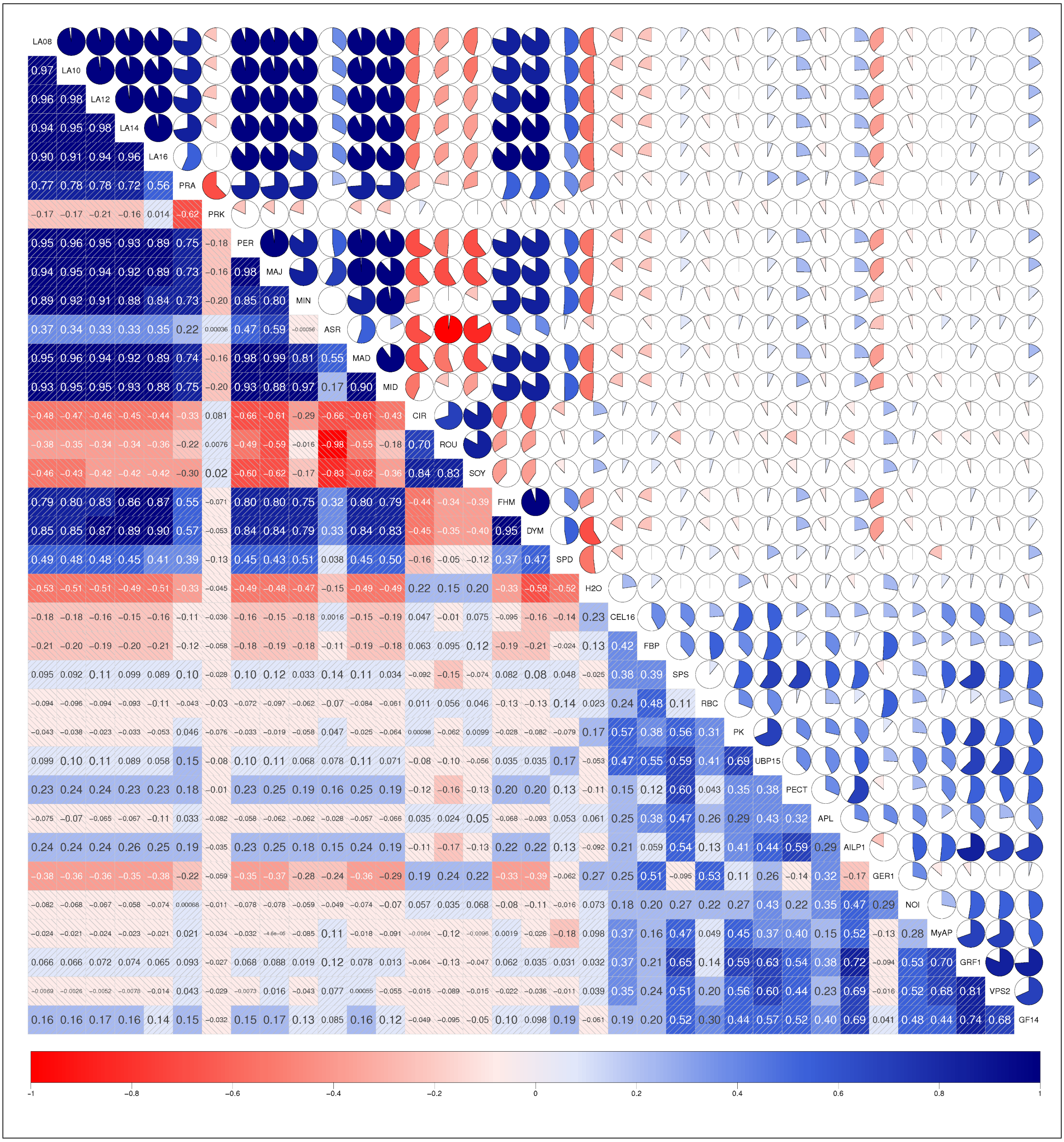

Figure S35. Correlations of the seedling development traits and the gene expression levels across all 509 inbreds. In the diagonal panel the traits and candidate genes are listed. In the upper panel the filled portion of the pie and in the lower panel the depth of the shading indicated the magnitude of the correlations. Negative correlations are colored red and positive correlations blue.

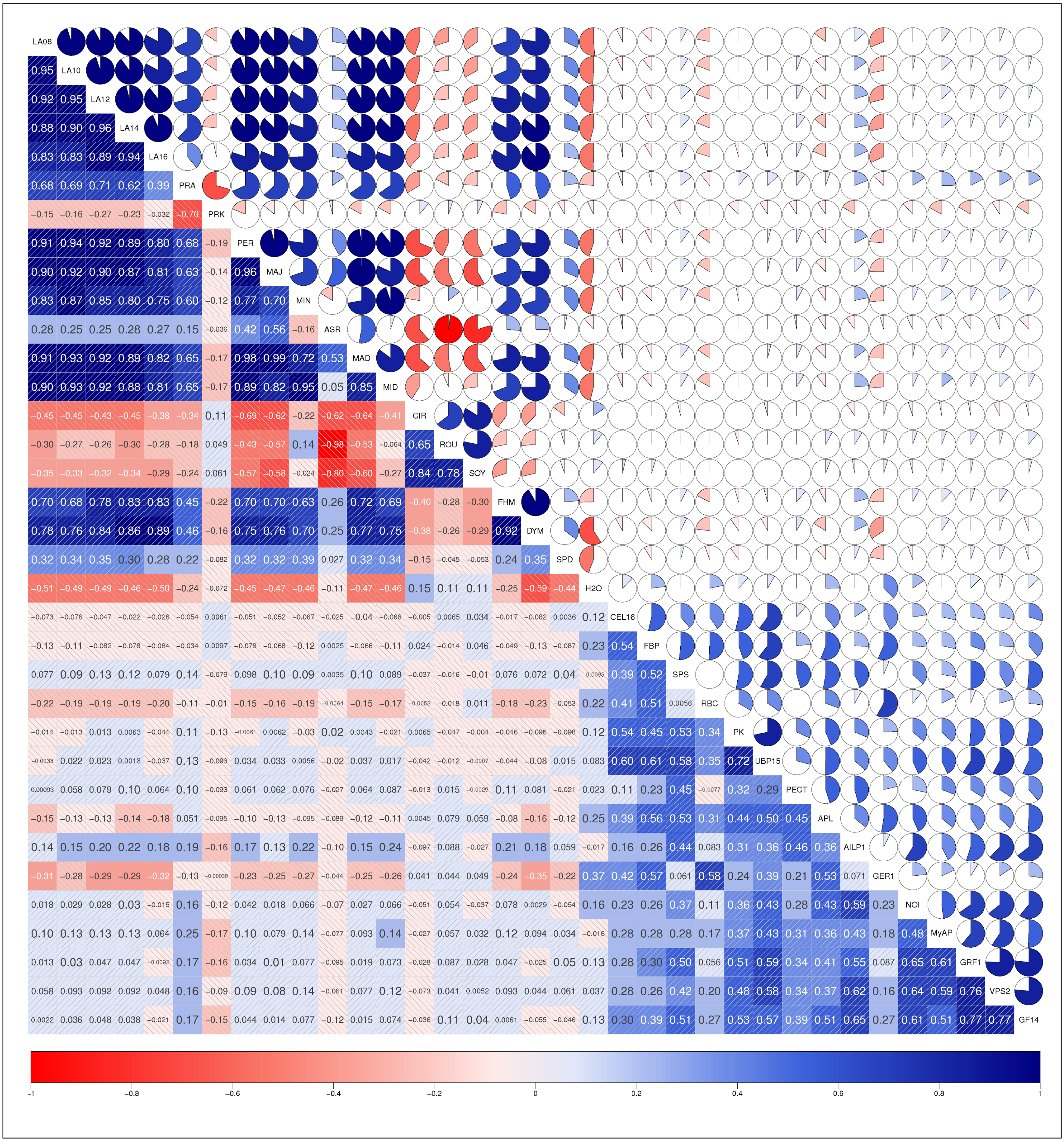

Figure S36. Correlations of the seedling development traits and the gene expression levels across the inbreds of the MCLUST group 1. In the diagonal panel the traits and candidate genes are listed. In the upper panel the filled portion of the pie and in the lower panel the depth of the shading indicated the magnitude of the correlations. Negative correlations are colored red and positive correlations blue.

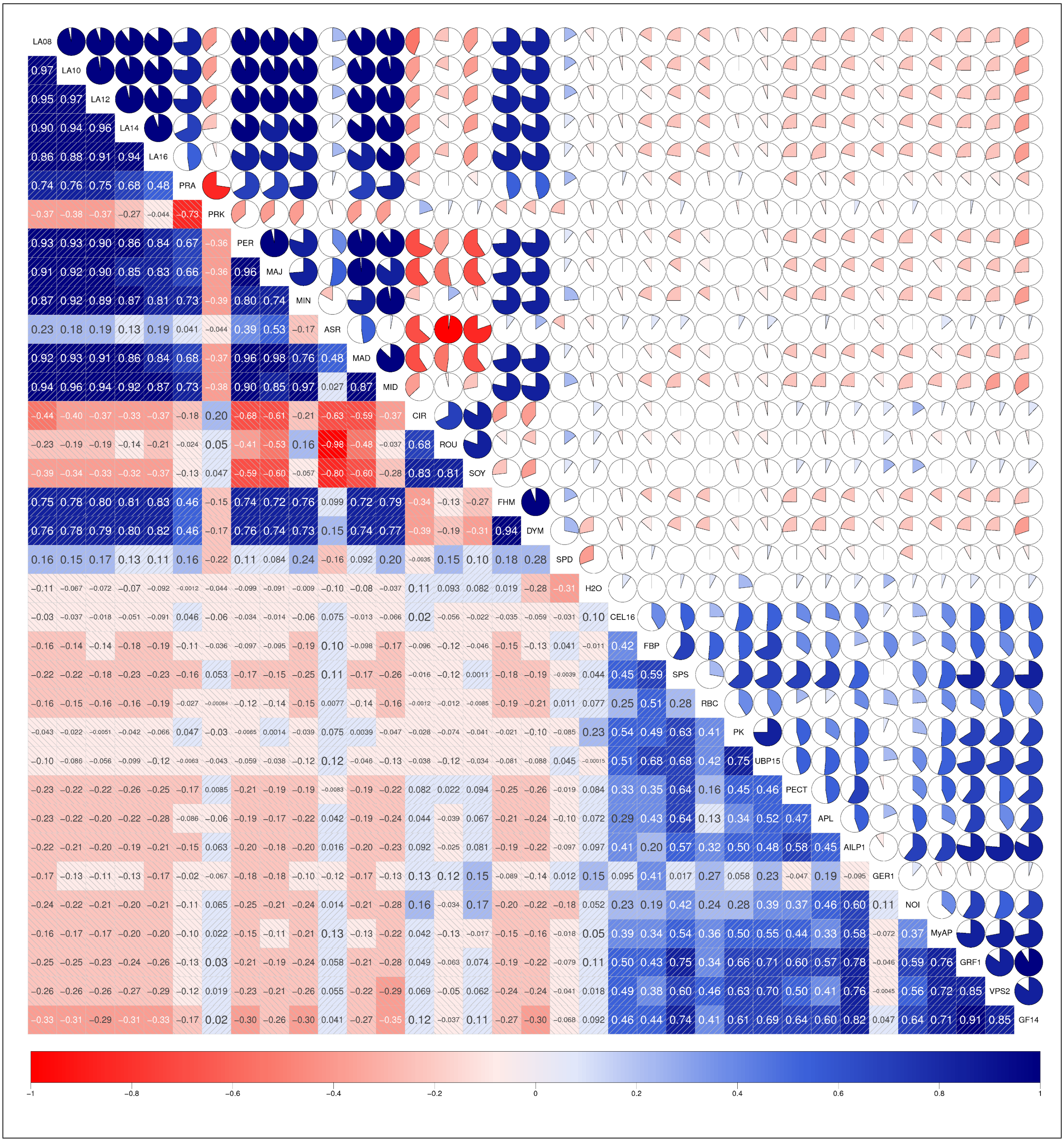

Figure S37. Correlations of the seedling development traits and the gene expression levels across the inbreds of the MCLUST group 2. In the diagonal panel the traits and candidate genes are listed. In the upper panel the filled portion of the pie and in the lower panel the depth of the shading indicated the magnitude of the correlations. Negative correlations are colored red and positive correlations blue.

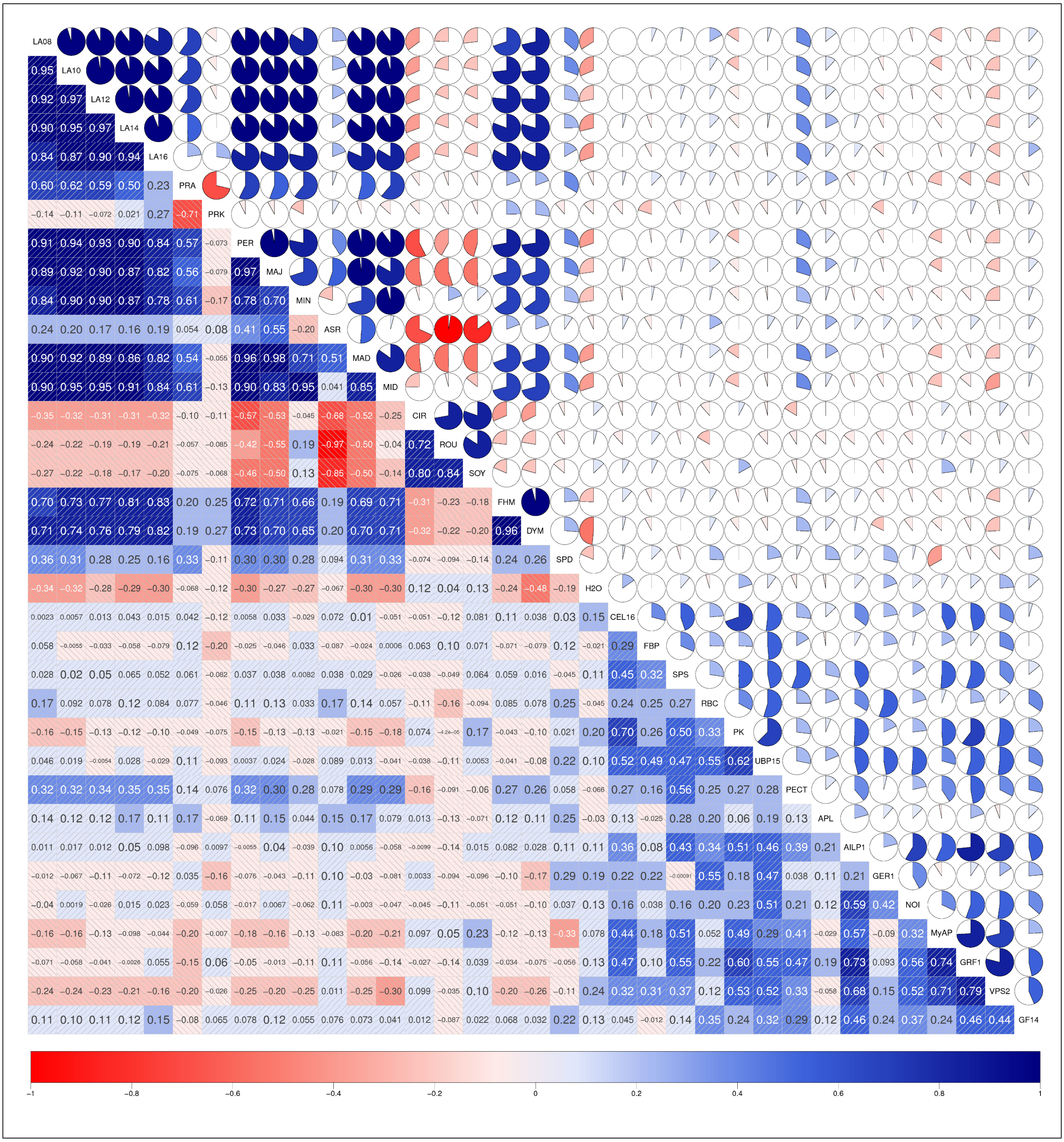

Figure S38. Correlations of the seedling development traits and the gene expression levels across the inbreds of the MCLUST group 3. In the diagonal panel the traits and candidate genes are listed. In the upper panel the filled portion of the pie and in the lower panel the depth of the shading indicated the magnitude of the correlations. Negative correlations are colored red and positive correlations blue.
